# A Genome-Wide Analysis of Serine Protease Inhibitors in *Cydia pomonella* Provides Insights into Their Evolution and Expression Pattern

**DOI:** 10.3390/ijms242216349

**Published:** 2023-11-15

**Authors:** Qiang Wu, Longsheng Xing, Min Du, Cong Huang, Bo Liu, Hongxu Zhou, Wanxue Liu, Fanghao Wan, Wanqiang Qian

**Affiliations:** 1 Shenzhen Branch, Guangdong Laboratory of Lingnan Modern Agriculture, Genome Analysis Laboratory of the Ministry of Agriculture and Rural Affairs, Agricultural Genomics Institute at Shenzhen, Chinese Academy of Agricultural Sciences, Shenzhen 518120, China; wuqiang@caas.cn (Q.W.); huangcong@caas.cn (C.H.); liubo03@caas.cn (B.L.); 2 College of Life Sciences, Hebei Basic Science Center for Biotic Interactions, Institute of Life Sciences and Green Development, Hebei University, Baoding 071000, China; xinglongsheng@caas.cn; 3 Shandong Province Key Laboratory for Integrated Control of Plant Diseases and Insect Pests, Sino-Australian Joint Research Institute of Agriculture and Environmental Health, College of Plant Health & Medicine, Qingdao Agricultural University, Qingdao 266109, China; charisdumin@163.com (M.D.); hxzhou@qau.edu.cn (H.Z.); 4 State Key Laboratory for Biology of Plant Diseases and Insect Pests, Institute of Plant Protection, Chinese Academy of Agricultural Sciences, Beijing 100193, China; liuwanxue@caas.cn

**Keywords:** serine protease inhibitor, melanization, *Cydia pomonella*, innate immunity, alternative splicing

## Abstract

Serine protease inhibitors (serpins) appear to be ubiquitous in almost all living organisms, with a conserved structure and varying functions. Serpins can modulate immune responses by negatively regulating serine protease activities strictly and precisely. The codling moth, *Cydia pomonella* (L.), a major invasive pest in China, can cause serious economic losses. However, knowledge of serpin genes in this insect remain largely unknown. In this study, we performed a systematic analysis of the serpin genes in *C. pomonella*, obtaining 26 serpins from the *C. pomonella* genome. Subsequently, their sequence features, evolutionary relationship, and expression pattern were characterized. Comparative analysis revealed the evolution of a number of serpin genes in Lepidoptera. Importantly, the evolutionary relationship and putative roles of serpin genes in *C. pomonella* were revealed. Additionally, selective pressure analysis found amino acid sites with strong evidence of positive selection. Interestingly, the *serpin1* gene possessed at least six splicing isoforms with distinct reactive-center loops, and these isoforms were experimentally validated. Furthermore, we observed a subclade expansion of serpins, and these genes showed high expression in multiple tissues, suggesting their important roles in *C. pomonella*. Overall, this study will enrich our knowledge of the immunity of *C. pomonella* and help to elucidate the role of serpins in the immune response.

## 1. Introduction

Serine protease inhibitors (serpins), which are evolutionarily ancient and show a conserved structure but functional diversity, are a superfamily distributed among almost all kingdoms of life [1,2,3]. Serpins are a relatively large group of proteins, typically composed of approximately 350–500 amino acids that fold into a conserved and metastable tertiary structure [4,5,6,7,8]. Over 80 serpin proteins have been structurally determined, and typical serpins share a conserved structure that contains 8–9 α-helices and three β-sheets [9,10] and possess a reaction-center loop (RCL) near the carboxyl-terminal end, which acts as “bait” and interacts with the active site of the target enzyme [7,11]. The selective specificity of a serpin is determined by the P1-P1′ cleavage sites on the RCL [2,10,12]. It is well known that the serpin has a unique suicide mechanism. The active site is disabled and the serpin is permanently inactivated by conformational changes after interaction with the target protease [1,12,13].

In insects, serpins play a critical role in physiological processes and immune responses by regulating serine protease activities [14,15,16]. With the completion of insect genome sequencing projects, more and more serpin genes have been identified in insects. So far, 29 serpins have been reported in *Drosophila* from the order Diptera [17] and 31 serpins in *Tribolium castaneum* from the order Coleoptera [18], while only five serpins were found in *Apis mellifera* from the order Hymenoptera [19]. A large number of serpin genes have been reported in Lepidoptera insects, ranging from 22 in *Helicoverpa armigera* [20] to 34 in *Bombyx mori* [21].

Recently, the functional roles of some insect serpins have been characterized; however, the functions of many serpins in insects remain to be investigated. It has been generally believed that serpin negatively regulates the prophenoloxidase (PPO) activation cascade [2,22,23] and other defense pathways (e.g., the Toll pathway) [24,25]. Previous studies demonstrated that serpins function mainly by inhibiting the activity of serine proteases, thereby maintaining homeostasis in the host and preventing harm from an excessive immune response [16,26,27,28,29]. Multiple studies have reported the role of serpins in the innate immune response of insects. In *D. melanogaster*, the serpin 43Ac (necrotic) regulates the activation of the Toll pathway after a fungal challenge [24,30]. In *Manduca sexta*, genetic and biochemical studies indicate that serpin1–7 are inhibitory, with the exception of serpin2 [31,32], and that they are involved in the regulation of the PPO activation cascade [32,33,34,35,36,37,38,39,40,41]. In *B. mori*, serpin5 can activate necrotic expression and inhibit the Toll pathway [42]. Additionally, serpin2 and serpin5 have been shown to participate in the silkworm anti-viral response [43]. Interestingly, serpin5 and serpin9 were found to promote baculovirus infection in *H. armigera* [44]. In addition to their roles in immunity, serpins can function in development, physiology, ecdysis, and metamorphosis. For instance, BmSPN2 is associated with digestion and nutrient absorption [45,46]. Spn88Ea is necessary for wing development in fruit flies [47]. Serpin27a regulates the formation of the dorsal–ventral axis of *Drosophila* embryos [48,49]. Serpin7 can affect energy metabolism and the diapause of migratory locust embryos by regulating polyphenoloxidase [8].

At present, the relevant research on serpins mainly focus on some model insects. The codling moth (*Cydia pomonella*) is a major invasive pest of pome fruits [50]. However, little is known about the biochemical and functional properties of serpins in this insect. Thus, the systematic identification of serpin genes is necessary for further functional analyses in the codling moth. To this end, the available codling moth genome was employed to annotate serpin genes and to analyze their phylogenetic relationships across multiple insects. Additionally, the characteristics and structure–function relationship of *C. pomonella* serpins were predicted. Moreover, the expression profile of serpin genes was revealed by transcriptome data. Overall, our study provides a dataset that will be a useful resource for further functional investigations of serpins in invasive and non-model insects.

## 2. Results

### 2.1. Overview of C. pomonella Serpins

To obtain a comprehensive set of serpin genes in the codling moth, similarity-search and homology-based annotation strategies were combined to identify serpin genes in the *C. pomonella* genome. The result showed that a total of 26 serpins were identified in the *C. pomonella* genome (Table 1). The coding sequences and deduced amino acid sequences of serpin genes in the codling moth are presented in Table S1.

A typical mature serpin protein consists of 300–400 amino acids, and almost all *C. pomonella* serpins were in accordance with the typical serpin size (345~570 residues) (Table 1), while CpSPN25 and CpSPN26 showed extreme sequence lengths, which might be partially due to the existence of other domains (Figure S1). In addition, CpSPN22 showed a shorter amino acid sequence than typical serpins. In *C. pomonella*, most serpin proteins have a molecular weight of 38.9~63.3 kDa. Most of the *C. pomonella* serpins had pI values between 4.88 and 6.98, while CpSPN12, 14, 23, and 25 showed higher pI values, ranging from 7.72 to 9.11. Furthermore, the conserved motifs and domain structure of serpin genes in the codling moth were also predicted (Figure S2). In total, fourteen serpins have signal peptides (Table 1; Figure S2C), indicating that they are potentially secreted proteins. Taken together, the general features of *C. pomonella* serpins are similar to those of serpins in *M. sexta* and *B. mori* [21,29].

It has been demonstrated that multiple regions play an important role in controlling and modulating the conformational changes in serpin, including the hinge, breach, shutter, and gate, which permit efficient and rapid insertion of the RCL into beta-sheet A [5,7,51]. The *C. pomonella* serpins were aligned with the structurally well-defined *M. sexta* serpin 1K [9]. Multiple-sequence alignment indicated that most of the *C. pomonella* serpins possess a consensus structure composed of three beta-sheets (sA-C), nine alpha-helices (hA-I) and an RCL (Figure S1). The results reveal that most residues in the important structural regions are conserved in most of the *C. pomonella* serpins.

### 2.2. Gain-and-Loss Analysis of the Serpin Gene Family across Lepidoptera Insects

**Figure 1:**
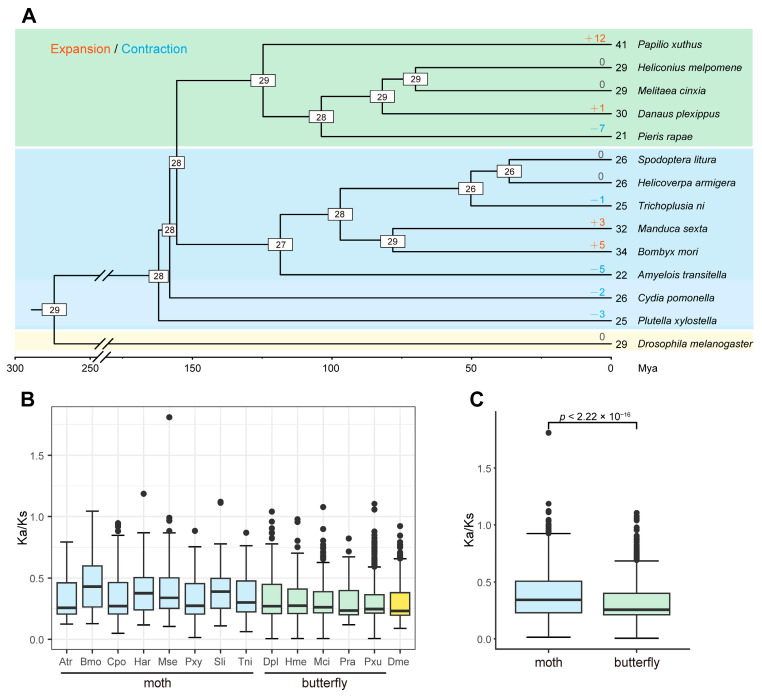
Estimation of serpin gene gains and losses and evolutionary rate analysis across thirteen lepidopteran species. (**A**) Gain-and-loss analysis of the serpin gene family. The divergence times in the species tree were estimated by r8s. The number of serpin genes in each species is indicated at the leaf nodes. The species whose serpin genes were collected from the literature are as follows: *M. sexta*, *B. mori*, and *P. xylostella*. In addition to *C. pomonella*, serpin genes were identified from nine other lepidopteran species and an outgroup species, *D. melanogaster*. Additionally, the number of serpin genes in *H. armigera* was corrected in this study. The numbers at the internal nodes represent gene numbers in the corresponding ancestors. The numbers of expanded and contracted genes are shown above the branches, with “+” representing gene gains while “−” denotes gene losses. Mya is the abbreviation for million years ago. (**B**) Boxplot showing the distribution of Ka/Ks ratios of paralog pairs in each species. Serpin genes within each species were paired, and the Ka/Ks ratios were estimated. (**C**) Comparison of Ka/Ks ratios of paralog pairs in two groups of lepidopteran insects. Statistical significance was determined using the Wilcoxon rank–sum test.

To estimate the gain and loss of serpin family members in Lepidoptera, CAFÉ analysis was performed based on thirteen Lepidoptera insects, including eight moths and five butterflies, and on an outgroup species, *D. melanogaster*. The results showed that twenty-eight serpin genes were estimated at the common ancestor node of Lepidoptera insects (Figure 1A). Compared with their most recent ancestors, the extant Lepidoptera insects have undergone a variety of gene gains or losses (ranging from 0 to 12). For example, seven serpin genes were lost in *Pieris rapae* (−7), and twelve serpin genes were gained in *Papilio xuthus* (+12), while no change was detected in *Heliconius melpomene* and *Melitaea cinxia*. As with moths, three and five genes were gained in *M. sexta* (+3) and *B. mori* (+5), respectively. Additionally, the losses of five, two, and three serpin genes occurred in *Amyelois transitella* (−5), *C. pomonella* (−2), and *Plutella xylostella* (−3) compared with the common ancestor.

To compare the evolutionary rate of serpin genes within each species, we calculated the Ka/Ks ratios of the serpin paralogs within each species. Generally, the Ka/Ks ratios of most paralog pairs were less than 1 in all species, suggesting that most serpin genes were subjected to purifying selection within the species. However, the Ka/Ks ratios also showed a difference between two groups of Lepidoptera species. Interestingly, the median ratios were stable in the butterflies, ranging from 0.2335 in *P. rapae* to 0.2724 in *H. melpomene*. By contrast, the ratios varied greatly in the moths (median: 0.2557–0.4306) (Figure 1B). Additionally, the moths showed significantly higher Ka/Ks ratios than did the butterflies (Figure 1C), suggesting that the serpin gene family might have evolved more rapidly in moths than in butterflies.

### 2.3. The RCL Region of C. pomonella Serpins

The RCL provides an ideal bait for the target proteinase, and its amino acid sequence determines the specificity of the serpin; specifically, the peptide chain is cleaved between the P1 and P1’ sites [10,17]. For the inhibitory serpins, it has been reported that there is a conserved consensus pattern, P_17_[E]-P_16_[E/K/R]-P_15_[G]-P_14_[T/S]-P_13_[X]-P_12-9_[A/G/S], in the hinge region [52]. Herein, we predicted the inhibitory activity of *C. pomonella* serpins mainly based on the presence of a potential P1 site.

**Figure 2:**
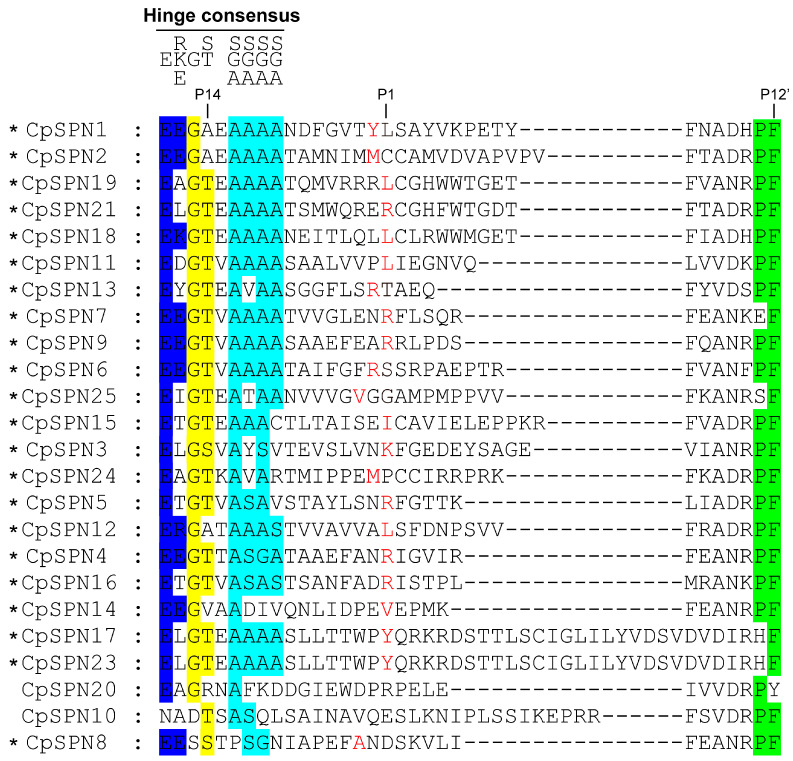
Multiple-sequence alignment of the reactive-center loop (RCL) regions in *C. pomonella* serpins. The conserved amino acids in the hinge region of inhibitory serpins are displayed above the alignment. The P1 sites, which determine substrate specificity, are highlighted in red. An asterisk indicates the inhibitory activity of the serpin. Serpin22 was not included in the alignment due to the lack of an RCL region. Serpin26 was also excluded from the alignment due to its extremely long RCL region (Figure S1), which might interfere with its binding to the target protease. Additionally, the incorporation of serpin26 into the alignment of RCL regions would make it difficult to predict the P1 site.

The alignment of RCL regions showed that the majority of *C*. *pomonella* serpins have a consensus sequence typical of the RCL hinge region. The positions of the scissile bond in *C. pomonella* serpins were predicted (Figure 2). The RCL sequence of CpSPN17 was completely consistent with that of CpSPN23, implying that they may play the same role, but the length may affect RCL insertion. CpSPN10 and CpSPN20 were less conserved in the hinge region (Figure 2), indicating that they might be non-inhibitory serpins. Nonetheless, the physiological roles of CpSPN10 and CpSPN20 remain to be elucidated experimentally. Notably, CpSPN22 lacks an RCL region. Nearly forty amino acids were inserted into the RCL of CpSPN26, resulting in a longer RCL. CpSPN22 and CpSPN26 were likely to be non-inhibitory serpins due to the abnormal length of their RCL, which might influence their binding to target proteases. However, their functional roles require further evidence from experimental studies. CpSPN3–7, 9, 13, 16, and 21 were anticipated to regulate trypsin-like enzymes with an arginine or lysine located at the predicted P1 position. CpSPN1, 11, 12, 15, 17–19, and 23 may participate in inhibiting chymotrypsin-like serine proteases (SPs), with leucine, isoleucine, or tyrosine being located at the P1 position. CpSPN2, 8, 14 and 24–25 may serve as an elastase inhibitor, with small hydrophobic residues at the P1 site. Therefore, four serpin genes (CpSPN10, 20, 22, and 26) in the codling moth were predicted to be non-inhibitory serpins (Figure 2; Table 2).

### 2.4. Evolutionary Relationships among the Lepidoptera Insect Serpins

**Figure 3:**
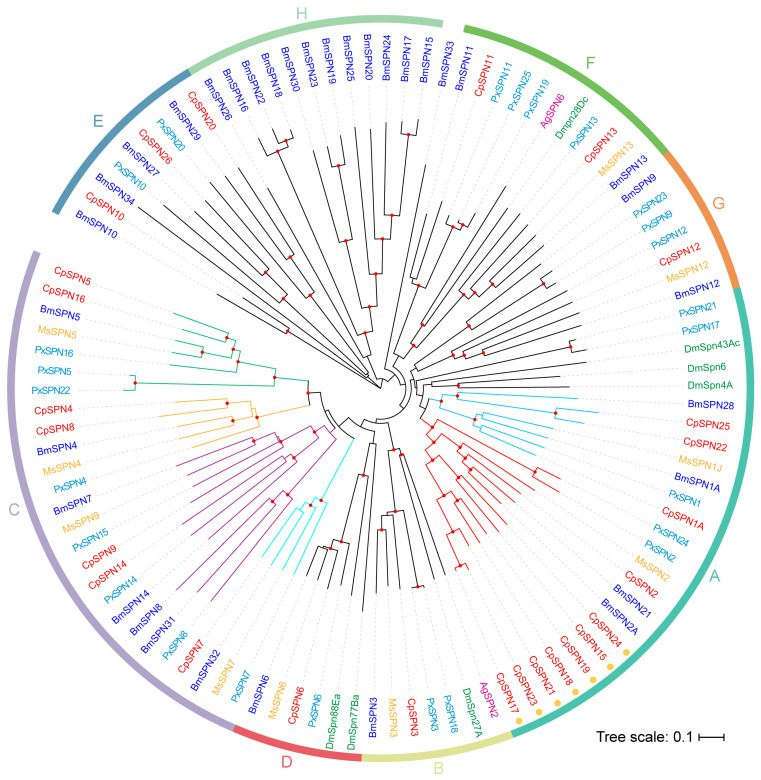
The neighbor-joining phylogenetic tree of serpins in insect species. Here, 26 *C. pomonella* serpins (red), 34 *B. mori* serpins (blue), 25 *P. xylostella* serpins (cyan), and known inhibitory serpins from *D. melanogaster* (green), *A. gambiae* (purple), and *M. sexta* (yellow) were included for constructing the phylogenetic tree. The phylogenetic tree was constructed using MEGA 11 with the neighbor-joining approach on the basis of the Poisson model and pairwise-deletion gaps. All nodes with bootstrap-supporting values higher than 70% are marked with red dots (1000 replications). These serpins were clustered into eight distinct groups (A–H). Seven members in group A that might have arisen from the rapid expansion of serpin genes in *C. pomonella* are indicated with orange circles around the gene IDs. Based on the topology, group A was further divided into two subgroups: A1 (red branch) and A2 (light-blue branch), and group C was further organized into four subgroups: C1 (green branch), C2 (orange branch), C3 (purple branch), and C4 (cyan branch). The scale bar represents 0.1 substitutions per amino acid position.

To investigate the evolutionary relationship of the serpin gene family, serpin genes from four Lepidoptera and two Diptera insects were selected to construct the phylogenetic tree. As shown in Figure 3, the serpins were divided into eight distinct groups (A–H) (Figure 3; Table 2). The homologous relationships for most *C. pomonella* serpins among the Lepidoptera species were 1:1, while the relationship with serpins from dipteran species was less clear.

Group A encompasses eleven *C. pomonella* serpins (1–2, 15, 17–19, 21–25). Most serpin genes in this group were composed of 7–9 exons, except for SPN22 (6 exons) and SPN25 (16 exons). CpSPN1A was clustered with PxSPN1, MsSPN1J, and BmSPN1A, and they had similar P_1_ residues (R for MsSPN1J, PxSPN1, and BmSPN1A; Y for CpSPN1A). MsSPN1J is capable of forming covalent complexes with prophenoloxidase-activating proteinase-3 (MsPAP3) and inhibits the pro-Spätzle-activating proteinase HP8, which may participate in the Toll pathway and PPO activation cascade [34,40]. A splicing isoform of CpSPN1, CpSPN1B share the same residue at P1 position with MsSPN1J (Figure S3). Therefore, we assume that CpSPN1 might also be involved in the regulation of the PPO pathway. The target enzyme of CpSPN1B may be similar to that of MsSPN1J, and CpSPN1A may function as the inhibitor of other enzymes in the PPO activation system.

CpSPN2 was clustered with BmSPN2, BmSPN21, and MsSPN2. In *B. mori*, *M. sexta* and the diamondback moth, serpin2 was considered an intracellular serpin due its lack of a signal peptide [21,29,53]. CpSPN2 had a signal peptide and was predicted to be an extracellular serpin. A subclade diverged from CpSPN2 at the same time, containing seven *C. pomonella* serpins (15, 17–19, 21, 23, 24). All serpins in this subclade lack classical signal peptides, suggesting that they may potentially be intracellular serpins. CpSPN15 and CpSPN24 share 57.88% sequence identity and are syntenic. CpSPN17–19, 21 and 23 reside on chromosome 15 (Table 2; Figure 3). In addition, CpSPN17, 21 and 23 showed a high level of sequence identity. High sequence identity and close genomic locations indicate recent gene duplications [21]. Serpin gene duplication has also occurred in the silkworm and other insects [18,21,54]. The rapid expansion gave rise to seven serpin genes in *C. pomonella* group A. However, this is fewer than those caused by serpin gene expansions reported in silkworm (11) [21] and *Drosophila* (18) [54].

In group B, CpSPN3 was clustered with *M. sexta*, *B. mori* serpin3, *P. xylostella* serpin-3, 18, AgSPN2, and DmSpn27A. Notably, serpin3 proteins in four Lepidoptera species share the proteinase cleavage sites (P1/P1′ = K/F). In *M. sexta*, MsSPN3 has been reported to be an inhibitor of PAPs [22]. It has been reported that AgSPN2 and DmSpn27A can participate in the regulation of melanization cascade [35,55]. We hypothesize that CpSPN3 might play an important role in the melanization cascade.

Group C contains many serpin members, including seven *C.* *pomonella* serpins (4, 5, 7–9, 14, 16), seven *B. mori* serpins (4, 5, 7, 8, 14, 31, 32), eight *P. xylostella* serpins (4, 5, 7, 8, 14–16, 22), and four *M. sexta* serpins (4, 5, 7, 9). Most genes in this group are encoded by a single exon; some serpins are composed of two or three exons (e.g., CpSPN9, 14). CpSPN4 shares an arginine residue at the P1 position with BmSPN4, MsSPN4 and PxSPN4, while CpSPN8 has a different residue at the P1 site because of gene duplication in *C. pomonella*. The same residue occurs at the P1 position of serpin5 and serpin7 in four insects. Additionally, nearly all of the group C members contain a signal peptide for secretion. Previous studies demonstrated that serpin4 and serpin5 inhibit the hemolymph proteinases HP21 and HP6 in *M. sexta*, respectively, which are the direct activators of PPO [37]. Serpin7 plays a role in regulating the PPO activation cascade and immune responses in *M. sexta* [32]. BmSPN32, an ortholog of MsSPN7, might also inhibit the activation of PPO by binding with BmPAP3 [56]. *B. mori* serpin5 negatively regulates melanization and AMP production by inhibiting hemolymph protease 6 (HP6) and serine protease 21 (SP21) [42,56]. This group of serpins in *C*. *pomonella* may play a similar role in the melanization cascade.

Group D is composed of one 1:1:1:1 ortholog, serpin6, DmSpn77Ba and DmSpn88Ea, thus allowing us to predict the putative functions of these serpins in *C. pomonella*. Serpin6 genes in four insects have a high sequence identity (69.42%) and share an arginine residue at the P1 position. The majority of serpins in group D have a signal peptide for secretion. Previous studies have demonstrated that serpin6 inhibits PAP3 and HP8 [57]. DmSpn88Ea is essential for wing unfolding and expansion in fruit flies [58]. The serpins in this group are relatively conserved and predicted to be secretory inhibitory proteins that modulate the melanization immune response.

In group E, BmSPN34, which lacks a conserved carboxyl-terminus (including the RCL), is not anticipated to fold properly [21]. CpSPN26 lacks an RCL as well. CpSPN10, CpSPN20, BmSPN27, BmSPN29, PxSPN10, and PxSPN20 are less conserved in their hinge regions. This group may not be related to protease inhibition. In a previous study [21], BmSPN10, 27, 29, and 34 formed an independent branch, while they have homologous genes with *P*. *xylostella* and *B. mori* in our evolutionary tree. Group E serpins are evolutionarily ancient, and their functions are unknown.

Group F contains *C. pomonella*, *P. xylostella,* and *B. mori*, serpin11 and serpin13, MsSPN13, PxSPN19, PxSPN25, AgSPN6, and DmSpn28Dc. A biochemical study confirmed that AgSPN6 is an inhibitor of trypsin-like serine proteinases and controls the melanization response by inhibiting the PPO activation pathway [59].

Group G is also composed of one 1:1:1:1 ortholog, serpin12, PxSPN9, PxSPN23, and BmSPN9. Serpin12 genes in four insects share 41.08% sequence identity, with a leucine residue at the P1 position. Serpin12 genes do not have signal peptides. Previous studies reported that *M. sexta* serpin12 regulates the PPO activation system by inhibiting hemolymph protease-14 (HP14), an initiating protease of the cascade [60]. Thus, the serpins in this group were assumed to function as inhibitors.

Group H is an independent clade that was completely composed of *B. mori* serpins, representing a unique expansion of serpin genes in the silkworm, as reported previously [21].

Furthermore, we employed the CODEML program with a site-specific model to test whether natural selection acted on the orthologous/paralogous serpin genes for each group. Herein, groups A and C were further divided into two (A1 and A2) and four subgroups (C1–4) (Figure 3; Table 2). Based on the M0 model, the *d*_N_/*d*_S_ values of the 12 groups ranged from 0.00763 to 0.35895 (Table 3), suggesting that the serpin genes had been subjected to strong purifying selection. However, the comparison between the M0 and M3 models indicated that the selective pressure varied between amino acid sites in all comparison groups. Further comparison between the M7 and M8 models showed that groups A1, D, and G exhibited evidence of positive selection (Table 3). However, the Bayes empirical Bayes (BEB) analysis showed that only five and one significant positively selected sites (PSSs) existed in groups A1 and G, respectively, while the PSS in group D is not statistically significant (BEB posterior probability = 0.733) (Table 3).

### 2.5. Collinearity and Chromosomal Location of Serpin Genes

**Figure 4:**
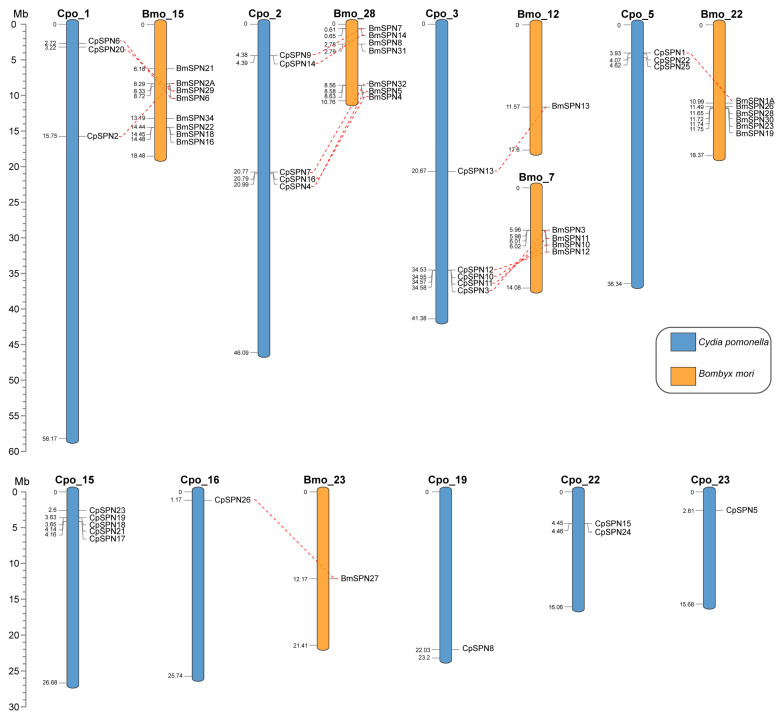
Chromosomal location of serpin genes in *C. pomonella* (blue) and *B. mori* (orange). Red dashed lines represent the corresponding orthologs between *C. pomonella* and *B. mori* serpin genes.

All 26 serpin genes were unevenly distributed on nine chromosomes of *C. pomonella* (Figure 4), mainly on chromosomes 1, 2, 3, 5, and 15, and the other four chromosomes only harbored a few serpin genes. Each of chromosomes 2, 3, and 15 contain five serpin genes, and together with the three serpin genes on each of chromosomes 1 and 5, these account for 80.77% of the repertoire of serpin genes in *C. pomonella*. In total, fifteen *C. pomonella* serpin genes have orthologs in *B. mori*. Interestingly, the serpin genes on chromosomes 1, 2, and 3 of *C. pomonella* show orthologous counterparts in *B. mori*, while no orthologs were detected for serpin genes on chromosome 15. Furthermore, the five *C. pomonella* serpin genes on chromosome 2 (CpSPN4, 7, 9, 14, and 16) and their orthologs in *B. mori* were clustered into group C in the phylogenetic tree (Figure 3). The five *C. pomonella* serpin genes on chromosome 3 (CpSPN3, 10, 11, 12, and 13) were clustered into different groups in the phylogenetic tree (Figure 3). Additionally, the five (CpSPN17, 18, 19, 21, and 23) and two serpin genes (CpSPN15 and 24) on chromosomes 15 and 22 clustered into group A, representing a unique expansion of serpin genes in *C. pomonella*, coinciding with the lack of orthologous counterparts in *B. mori* for these serpin genes.

### 2.6. Alternative Splicing Analysis of the Serpin1 Gene in C. pomonella

**Figure 5:**
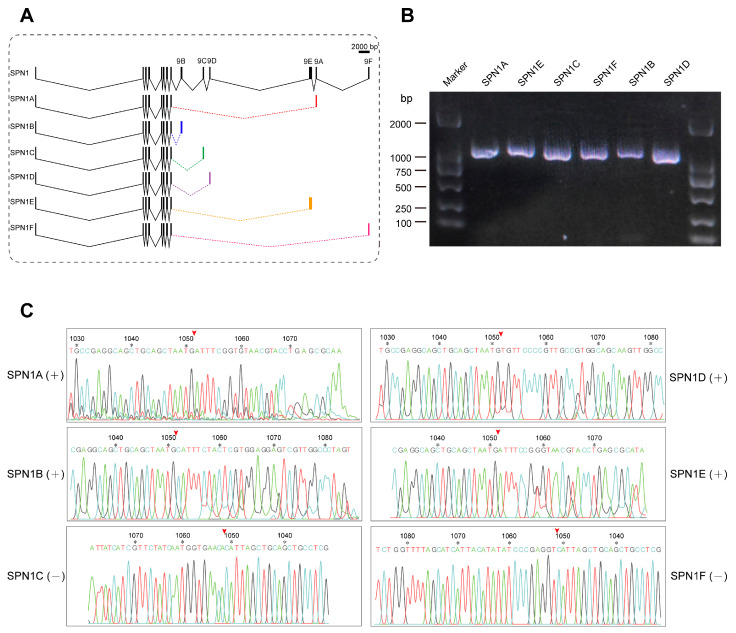
Comparison of gene structures of the *C. pomonella serpin1* isoforms and reverse transcription–polymerase chain reaction (RT-PCR) analysis. (**A**) Exon–intron structures of serpin1 alternative splicing isoforms. Black boxes represent exons, polylines represent introns, and colored boxes represent alternative ninth exons. Exons 1–8 were in common among the different serpin transcripts. The carboxyl-terminal regions encoding the reactive-center loop were different among the splice isoforms. (**B**) Validation of *serpin1* isoforms using RT-PCR analysis. An agarose gel electrophoresis photograph of the PCR products showed that all the serpin-1 isoforms migrated to the expected locations. The marker positions are labelled on the left and right side. (**C**) Sanger-sequencing chromatograph peaks showing the boundary sequences between the eighth and ninth exons. The red arrowhead above the sequence indicates the splice site between the eighth and ninth exons for each *serpin1* isoform. The number and the asterisk above the nucleotide indicate the position of the corresponding nucleotide in complete coding sequence.

Previous studies have reported that many splicing isoforms exist for the *serpin1* gene in multiple insects such as *M. sexta* [29], *B. mori* [21], and *Pteromalus puparum* [61]. CpSPN1 is capable of encoding six isoforms with varied RCL sequences via alternative splicing. The CpSPN1 gene is composed of ten exons, and the difference only lies in the ninth exon, which encodes the RCL. Due to the differences in the RCL region, serpin1 isoforms might have different inhibitory spectra. As shown in Figure 5A, the cDNA length of serpin1 isoforms was in the range of 1048–1099 bp. The expression of six CpSPN1 splice variants was validated using RT-PCR analysis. The results verified that the six alternative isoforms of CpSPN1 were indeed expressed in the codling moth (Figure 5B). Additionally, the sequences flanking the ninth exon of the splicing isoforms were further validated using Sanger sequencing (Figure 5C). Multiple-sequence alignment showed that serpin1 proteins exhibit high sequence similarity and conservation in the hinge region preceding the RCL across insects (Figure S3A). Additionally, we compared the RCL regions of CpSPN1 splice isoforms. The results showed that the RCLs were poorly conserved (Figure S3B), indicating the divergent target specificities of CpSPN1 isoforms. The large hydrophobic residues (Tyr or Leu) at the P1 site of CpSPN1A and CpSPN1E likely imply their roles in the regulation of chymotrypsin proteases. CpSPN1B and CpSPN1C were predicted to be trypsin inhibitors with a charged P1 residue. CpSPN1D and CpSPN1F possessed a hydrophobic residue (Ala or Val) at the P1 site and were presumed to be elastase inhibitors. Together, we verified the existence of six *serpin-1* alternative isoforms, and the alternative splicing of *serpin1* could efficiently increase the diversity of serpin repertoire in *C. pomonella*.

### 2.7. Expression Profile of C. pomonella Serpins

**Figure 6:**
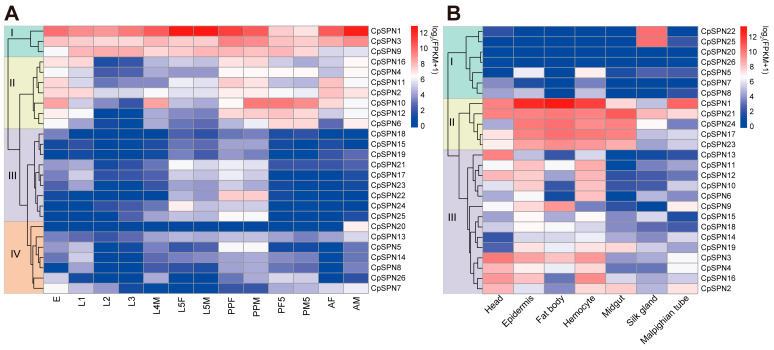
Expression profiles of *C. pomonella* serpins at different developmental stages (**A**) and in different tissues (**B**). Log_2_(FPKM+1) values for serpin genes were used to generate the heat map. Transcript levels ranging from low to high are represented by blue to red colors. Based on the expression pattern, all serpin genes were organized into four and three subgroups in the developmental stages and tissues, respectively. The abbreviations used are as follows: E, eggs; L1–L4, 1st- to 4th-instar larvae; L5F/M, 5th-instar female/male larva; PPF/M, female/male pre-pupa; PF/M1, female/male 1^st^-day pupa; PF/M5, female/male 5th-day pupa; AF, female adult; AM, male adult. All the tissue samples were collected from 4th-instar larvae.

Through RNA-seq analysis, the expression profile of *C. pomonella* serpins in different developmental stages and tissues was elucidated, exhibiting a specific pattern in time and space. Apparently, serpin genes were organized into four subgroups based on their expression pattern in various life stages of the codling moth (Figure 6A; Table 2). For group I, CpSPN1 was highly expressed in almost all stages, with the highest expression level in 5th-instar larvae and adult males. Similarly, CpSPN3 showed high expression levels over the entire life cycle. The CpSPN9 mRNA level was abundant in larvae, pre-pupae, and pupae, indicating its potential role in early development. For group II, these genes showed high transcript levels in the egg, 1st-instar larva, pre-pupa, pupa, and adult. Regarding CpSPN10 showing the highest expression level in the pre-pupal and pupal stages, we hypothesize that it might play an important role in the physiological activity of the pupal stage. For group III, these genes showed higher transcript levels in 5th-instar larvae and pre-pupae, while they were present at a lower level in other stages. Among them, CpSPN22 displayed the most abundant level in the pre-pupa period. For group IV, most of these genes were maintained at a relatively lower expression level across all stages, with the exception of CpSPN5 (higher expression in the pre-pupa), 7 (higher expression in the egg), and 20 (higher expression in the male adult). Interestingly, many genes (CpSPN4, 6, 10–12, 16, 20) exhibited a sex-specific expression pattern in the adult stage, implying their distinct roles between male and female adults.

In addition, *C. pomonella* serpins exhibited tissue-specific expression patterns in different tissues of the 4th-instar larva (Figure 6B; Table 2). According to their expression profile, 26 serpin genes were also clustered into three subgroups. For group I, CpSPN5 and 8 showed moderate expression levels in the epidermis and hemocyte, while CpSPN20 and 26 were lowly expressed in all tissues. Strikingly, CpSPN22 and 25 were only highly expressed in the silk gland, implying their critical roles in silk biosynthesis. For group II, almost all the genes were highly expressed in the epidermis, fat body, hemocyte, and midgut, indicating their potential roles in the defense response. In particular, CpSPN1 exhibited the most abundant levels in the fat body, epidermis, and hemocyte. Additionally, four of the five genes (CpSPN1, 17, 21, and 23) showed a higher expression in the head. For group III, the majority of these genes showed higher expression in the head, epidermis, fat body, and hemocyte. Additionally, CpSPN2 and 19 were highly expressed in the midgut, and CpSPN2–4 and 9 showed higher expression in the Malpighian tube. Almost all the genes in group III were lowly expressed in the silk gland. Together, the spatiotemporal expression pattern of *C. pomonella* serpin genes reveals their distinct roles in different developmental stages and tissues.

## 3. Discussion

Serpins are distributed throughout all branches of life and appear time and again to control proteolytic pathways related to insect growth and development and immune regulation with a unique mechanism of action [2,3]. Recently, genome-wide and transcriptome-wide analyses have helped us to identify serpin genes from several insect species, and the structure and function as well as evolutionary adaptation of serpins has been uncovered. In our study, a total of 26 serpin genes were identified in the *C. pomonella* genome. Serpins are distributed across nine different chromosomes, and a gene cluster of serpins might arise from tandem gene duplications. Rapid and large gene amplification appears to be quite extensive in the serpin family and may experience functional differentiation. The rapid expansion of group F in *B. mori* is a good example of this mechanism [21]. Eighteen serpins were rapidly amplified in *Drosophila* [21,54]. Similarly, a family expansion gave rise to seven serpin genes in *C. pomonella*. However, in the diamondback moth, which diverged at a similar time with the codling moth, there did not appear to be a large amplification of serpin genes. The amplified genes in *C. pomonella* are clustered in a small branch of group A, which emerged after the species differentiation, suggesting that it may be arisen from the recent gene replication event. The serpin genes rapidly amplified in the silkworm lack the characteristics of inhibitory serpins. By constrast, the serpin genes rapidly amplified in *C. pomonella* share the conserved sites in the RCL regions and were assumed to inhibit chymotrypsin enzymes. According to the phylogenetic analysis, this subgroup is most closely related to serpin2. PxSPN2 can inhibit Destruxin A, which is secreted by an entomopathogenic fungus and regulates the phenoloxidase (PO) activity and melanization [62]. BmSPN2 participates in the silkworm antiviral response and is associated with digestion and nutrient absorption [43,45,46]. These further indicate that the *Cydia* serpin genes in this subgroup are inhibitory serpins, and may play an important role in the regulation of the melanization and antiviral response. Additional biochemical and functional studies are required to determine their specific function and whether gene expansion is critical for resisting viral infection.

In addition to gene duplication, we identified six *serpin1* isoforms with distinct RCL regions and inhibitory spectrums due to the mutually exclusive alternative splicing of the ninth exon. The same strategy for serpin diversity also occurred in other Lepidoptera insects, as seen by the 14 serpin1 isoforms in *M. sexta* [29], and 4 serpin1 isoforms in *B. mori* [21,63]. However, the phenomenon has not been observed in mammals, suggesting that it may be common only among insects, especially in Lepidoptera. The alternative splicing of serpins might be a conservative mechanism during evolution, extending the inhibitory spectrum, which could potentially improve the immune defense.

Based on the alignment of full-length sequences and RCL regions, the conserved region and P1 sites were predicted, and almost all the serpins (22) were predicted as inhibitory. However, 13 of 34 serpins function as protease inhibitors in the silkworm [21]. In *M. sexta*, only 16 of 32 serpin genes are predicted to encode inhibitory serpins [29]. It is speculated that the difference in the number of inhibitory serpins among species might result from the variations in diet and environment. Although the total number of serpins in the silkworm is large, the inhibitory serpins are fewer [21], which might be related to the clean diet (mulberry leaves) and lower exposure to microorganisms in the environment. The larva of *C. pomonella* burrows into the fruit for feeding [50] and may encounter more pathogenic microbes; thus, more inhibitory serpins are required in *C. pomonella*. However, biochemical experiments are needed to elucidate the host–pathogen interaction.

Mseserpin8 was only present at a high level in 3 h eggs [29]. This was also true of Agserpin13 [29,38], leading Li et al. to suggest that there may be some conserved function for non-inhibitory serpins in eggs [29]. However, serpins with high expression in codling moth eggs are mostly inhibitors (Figure 6). Although the expression level of non-inhibitory serpin26 was high in the egg stage, it was higher in the pupa stage than in the egg stage. This may be related to the few non-inhibitory serpins in *C. pomonella*.

Based on the phylogenetic tree and expression patterns, the functions of serpins were inferred. The serpins that showed abundant transcript levels in the pre-pupal and pupal stages might have potential functions during pupation preparation and pupal development. The fat body, midgut, and hemolymph are three main organs of the immune system. A majority of the insect serpins are produced in the fat body and hemocytes and then secreted into the hemocoel [2]. Interestingly, nearly half of the serpin genes were highly expressed in the head of 4th-instar larvae (Figure 6). Although the head is not generally considered an immune organ in insects, it has been demonstrated that many immune-related proteins are expressed in the head of honey bees after a bacterial challenge, including signal-transduction proteins [64]. We suppose that serpins may play important roles in the immune defense or the development of the head. The midgut, a place where insects and pathogens interact, is related to immunity. Serpins have an opposite tissue distribution tendency when compared with SPs, which has been documented in the diamondback moth and silkworm. A large number of SPs generally distributed in the midgut are involved in various physiological processes, such as digestion, development, and the immune response [65]. In contrast, seven serpin genes that were generated by rapid amplification clustered together with serpin2, except for CpSPN18 and CpSPN25. Among them, six serpins were specifically expressed in the midgut. Therefore, the amplified serpins may be involved in the trypsin or chymotrypsin protease cascade reaction. In addition to the conserved serpin domain, three other domains were also detected in CpSPN25 (TIL domain) and CpSPN26 (PRK13335 and PRK08581 superfamilies) (Figure S2C). In fact, the TIL (trypsin-like inhibitor) domain has also been found in *P. xylostella* previously [53]. Based on a conserved-domain search, we found that the TIL domain was also present in serpins of nine other lepidopteran insects (*A. transitella*, *D. plexippus*, *H. armigera*, *H. melpomene*, *M. cinxia*, *P. rapae*, *P. xuthus*, *S. litura*, and *T. ni*) that were used for comparative genomics analysis. Previous studies have reported that the TIL domain-containing proteins are mainly involved in regulating the host immune response by inhibiting the protease cascade reaction [66,67,68] and by participating in the regulation of mosquito reproduction [69]; thus, we inferred that the function of CpSPN25 might be an immune inhibitor. As with the PRK13335 (annotated as superantigen-like protein 3 in the NCBI CDD) and PRK08581 (annotated as amidase domain-containing protein) superfamily domains, the former was only found in CpSPN26, while the latter was also detected in BmSPN27. Previously, it was found that superantigen-like protein 3 could antagonize Toll-like receptors to prevent receptor stimulation and promote bacterial pathogenicity [70,71]. Amidase is a common name for amide hydrolase, whose member, fatty acid amide hydrolase, has been implicated in human health and many physiological roles [72,73,74,75]. Thus, CpSPN26 might have diverse functions. Together, the functions of these serpin genes deserve further exploration.

The prediction of the RCL region is based on the conserved feature of the serpin sequences, the distance from the hinge, and the cleavage sites of previously identified serpins [10,33,47,57]. To determine the inhibitory selectivity and function of serpins in *C*. *pomonella*, experimental verification is required. Additionally, a single amino acid substitution in the RCL region of an inhibitory serpin can completely alter its substrate specificity [17,76]; thus, the identification of the P1 position is critical for future functional verification.

## 4. Materials and Methods

### 4.1. Identification of Serpin Genes in C. pomonella

Serpin protein sequences of *D. melanogaster*, *Anopheles gambiae*, *B. mori*, *M. sexta*, and *P. xylostella* were obtained from the literature and the NCBI (National Center for Biotechnology Information) (http://www.ncbi.nlm.nih.gov/ (accessed on 21 March 2021)) and then used as queries to search a protein database that was constructed from the *C. pomonella* official gene set (http://www.insect-genome.com/cydia/download.php (accessed on 22 March 2021)) using the BLASTP v2.2.26+ [77] program with the parameter “-evalue 1e-5”. The *C. pomonella* serpins that were identified in the reference annotation were used as queries to search the *C. pomonella* genome using TBLASTN v2.2.26+ with default settings to recover serpin genes that were possibly missed in the genome annotations. Then, novel serpin genes were identified using FGENESH v2.6 (Softberry, Inc., Mount Kisco, NY, USA) [78] (http://www.softberry.com/ (accessed on 5 April 2021)) and GeneWise v2.2.0 [79]. Subsequently, the protein sequences encoded by candidate serpins were submitted to the NCBI CDD database (http://www.ncbi.nlm.nih.gov/Structure/cdd/docs/cdd_search.html (accessed on 20 April 2021)), Pfam v34.0 [80] (https://pfam.xfam.org/ (accessed on 20 April 2021)), and SMART [81] (http://smart.embl-heidelberg.de/ (accessed on 20 April 2021)) to identify conserved domains. Finally, the corresponding entries with the conserved serpin domain were determined as serpin genes.

Additionally, the genomes of nine other lepidopteran insects were retrieved from NCBI, LepBase, and custom databases, including *S. litura* (NCBI RefSeq: GCF_002706865.1), *H. armigera* (NCBI RefSeq: GCF_002156985.1), *T. ni* (Cabbage Looper Database: http://cabbagelooper.org/ (accessed on 17 May 2021)), *A. transitella* (NCBI RefSeq: GCF_001186105.1), *P. xuthus* (NCBI RefSeq: GCF_000836235.1), *H. melpomene* (Butterfly Genome Database: http://butterflygenome.org/ (accessed on 17 May 2021)), *M. cinxia* (Butterfly Genome Database: http://butterflygenome.org/ (accessed on 17 May 2021)), *D. plexippus* (MonarchBase: http://monarchbase.umassmed.edu/ (accessed on 17 May 2021)), and *P. rapae* (NCBI RefSeq: GCF_001856805.1). For the identification of serpin genes in these species, the predicted proteome of each insect species was used for BLASTP alignment and protein-domain search against the conserved HMM profile (PF00079.19) using the HMMER3 program [82].

### 4.2. Feature Analysis of Serpin Protein Sequences

The theoretical molecular weight (Mw) and isoelectric point (pI) of the proteins encoded by codling moth serpin genes were calculated using ProtParam (https://web.expasy.org/protparam/ (accessed on 17 May 2021)) [83]. Signal peptides and cleavage sites were predicted using SignalP 5.0 (http://www.cbs.dtu.dk/services/SignalP/ (accessed on 17 May 2021)) [84]. Multiple-sequence alignment was performed using ClustalW with the default parameters, and the sequence alignment was manually modified using the GeneDoc 2.7 software (http://www.nrbsc.org/gfx/genedoc/index.html (accessed on 17 May 2021)). Subsequently, the secondary structures of codling moth serpins were predicted manually based on the domains previously assigned to *M. sexta* Serpin-1K [9,38].

### 4.3. Phylogenetic Analysis

To reveal the evolutionary relationship of serpin genes across insect species, multiple-sequence alignment of protein sequences encoded by serpin genes from four Lepidoptera insects (*C. pomonella*, *M. sexta*, *B. mori*, and *P. xylostella*) and two Diptera insects (*D. melanogaster* and *A. gambiae*) was performed using the MUSCLE algorithm [85] with the default settings. The phylogenetic tree was constructed based on the Jones–Taylor–Thornton (JTT) model with the neighbor-joining method [86] using MEGA 11 [87] with 1000 bootstrap replications. Gapped positions were treated by pairwise deletion. Poisson correction was used as a substitution model to determine pairwise distances. Finally, the phylogenetic tree was visualized and modified using ITOL v6.8.1 (https://itol.embl.de/ (accessed on 5 September 2023)) [88].

### 4.4. Gene Gain-and-Loss Analysis

The expansion and contraction analysis of serpin genes was performed using CAFÉ 3 [89], in which a birth and death model was employed to simulate the dynamics of gene family evolution. Firstly, *C. pomonella* and twelve other Lepidoptera insects, including seven moths (*P. xylostella*, *A. transitella*, *B. mori*, *M. sexta*, *T. ni*, *H. armigera*, and *S. litura*), and five butterflies (*P. rapae*, *D. plexippus*, *M. cinxia*, *H. melpomene*, and *P. xuthus*), and an outgroup species, *D. melanogaster*, were selected for the construction of a species tree. The phylogenetic species tree was reconstructed using the maximum likelihood method in RAxML [90] based on the strict single-copy orthologs of these lepidopteran insects identified by OrthoFinder analysis [91]. Then, the species divergence time was estimated using r8s [92] with the recalibration time points adopted from the TimeTree website (http://www.timetree.org/ (accessed on 17 May 2021)) as follows: 50–80 million years ago (Mya) for *B. mori* and *P. xylostella*; 70–90 Mya for *S. litura* and *T. ni*.

Gene numbers of the serpin gene family were collected from the publications for *B. mori* [21], *P. xylostella* [53], and *M. sexta* [29], while the number of serpin genes in *C. pomonella* and other insects was identified in this study. The number of serpin genes in *H. armigera* was updated from 22 [20] to 26. The divergence time-recalibrated species tree and the matrix of serpin gene numbers was taken as the input for gene family gain-and-loss analysis using CAFÉ 3.

### 4.5. Genomic Location and Synteny Analysis

The chromosomal locations of serpin genes in *C. pomonella* and *B. mori* were extracted from the genome annotation. Additionally, the orthologous serpin gene pairs between *C. pomonella* and *B. mori* were identified based on the reciprocal best BLAST hit. The distribution of serpin genes on the chromosomes was drawn using an in-house Python script (https://github.com/jackiexls/ChrLocPlotter (accessed on 18 May 2021)).

### 4.6. Estimation of the Synonymous and Nonsynonymous Rate Ratio

To compare the evolutionary rate of serpin genes between insect species, the serpin protein sequences were paired within each species. The paired serpin protein sequences were aligned using MUSCLE [85] and converted into codon alignments using PAL2NAL [93]. The nonsynonymous substitution rate (Ka), synonymous substitution rate (Ks), and Ka/Ks ratios were estimated using the KaKs_Calculator program [94] with the YN model.

### 4.7. Insect Rearing and Sample Collection

The original codling moth strain was collected from a conventional apple orchard located in Jiuquan (39.74° N, 98.50° E), Gansu Province, China, and was maintained in our laboratory as described previously [50]. Generally, after egg hatching, each individual neonate larva was transferred to separate plastic tubes containing an artificial diet. The larvae were kept in growth chambers at 26 ± 1 °C under a relative humidity of 60% and a photoperiod of 16 h:8 h (light:dark). The pupae were stored until their eclosion into adults, and the start of the next life cycle.

To examine the gene expression pattern in the codling moth, we collected samples from different developmental stages, including from eggs (50 individuals), 1st-instar larvae (50 individuals), 2nd-instar larvae (30 individuals), 3rd-instar larvae (10 individuals), mid-term 4th-instar larvae (5 individuals), 5th-instar females (3 individuals) and males (3 individuals) larvae, female (3 individuals) and male (3 individuals) pre-pupae, female (3 individuals) and male (3 individuals) pupae at day 5, and female (3 individuals) and male (3 individuals) adults. Additionally, seven different tissue samples were collected from 4th-instar larvae, such as from the head (10 individuals), silk gland (10 individuals), midgut (10 individuals), fat body (10 individuals), hemocyte (individuals), epidermis (5 individuals), and Malpighian tube (10 individuals).

### 4.8. RNA Sequencing (RNA-Seq) and Gene Expression Analysis

After total RNA extraction, the integrity of the RNA was evaluated using the Agilent Bioanalyzer 2100 system (Agilent Technologies, Palo Alto, CA, USA). Then, RNA-seq libraries were constructed for the above-mentioned samples with the Illumina TruSeq Stranded mRNA Library Prep Kit (San Diego, CA, USA). Three biological replicates were prepared for each sample. The cDNA libraries were paired-end sequenced on the Illumina HiSeq platform (Annoroad Gene Technology Company, Beijing, China).

Raw RNA-seq reads were filtered to remove the adapters, and low-quality and short sequences (<36 bp) were removed using Trimmomatic v0.38 [95]. The clean reads were mapped to the reference genome using Bowtie2 v2.4.5 [96]. RSEM v1.2.12 [97] was used for estimating the gene expression level, which was calculated as FPKM (fragments per kilobase of exon per million fragments mapped). The heat map of gene expression for serpin genes was generated based on log_2_(FPKM+1) values using the pheatmap package in the R environment (v3.6.1).

### 4.9. Identification and Cloning of C. pomonella Serpin1 Isoforms

Total RNA was extracted from 4th-instar larvae using TRIzol following the manufacturer’s instructions. RNA concentration was measured using the Implen Nanophotometer (Implen GmbH, Munich, Germany). A sample of 1 μg of total RNA was used for first-strand cDNA synthesis using the All-in-One Mix Kit (TransGen Biotech, Beijing, China). To detect the splice isoforms of the *serpin1* gene, specific primers were designed for each transcript using Primer 3 (Table S2). The PCR conditions were as follows: pre-denaturation at 95 °C for 3 min; followed by 35 cycles of denaturation at 95 °C for 30 s, annealing at 55 °C for 30 s, and extension at 72 °C for 30 s; sufficient extension at 72 °C for 5 min, and finally, maintenance at 12 °C. The PCR products for all the isoforms were separated by electrophoresis on a 1% agarose gel and visualized using a gel image system (BioRad Laboratories, Hercules, CA, USA). Additionally, the PCR products with unique bands were sent to a company (Sangon Biotech Co., Ltd., Shanghai, China) for direct Sanger sequencing. The sequencing of PCR products was performed using a proprietary chemistry based on Big Dye 3.1 on an Applied Biosystems 3730XL DNA Analyzer (Thermo Fisher Scientific, Waltham, MA, USA), and base calling was conducted using the Applied Biosystems Sequencing Analysis Software version 5.3.1.

### 4.10. Selective Pressure Analysis

To test whether the serpin genes underwent natural selection, we performed selective pressure analysis using the CODEML program in the PAML package (v4.9b) [98]. For each group of *serpin* genes in the phylogenetic tree, the site-specific model (model = 0, NSites = 0 3 7 8) was employed for testing which genes or sites had evolved under positive selection. The comparison between models was performed using the likelihood ratio test (LRT), and the chi-squared test was used for the determination of statistical significance. The positively selected sites (PSSs) were identified using the Bayes empirical Bayes (BEB) method, and the sites with BEB posterior probabilities of ≥0.95 were defined as PSSs.

## 5. Conclusions

In this study, the family members, sequence features, phylogenetic analysis, and expression pattern of serpins have been systematically characterized in *C. pomonella*. A total of 26 serpin genes have been identified in *C. pomonella*. Among them, fourteen serpins have a signal peptide for secretion. Most serpins are located within the normal size range, with the exception of CpSPN25 and CpSPN26. In addition to the conserved serpin domain, CpSPN25 and CpSPN26 also possess domains with unclear roles, suggesting that they might have additional functions. It was shown that 22 of the serpins might function as protease inhibitors. Additionally, comparative genomics analysis revealed the evolutionary history of the serpin gene repertoire in Lepidoptera insects. Combining the phylogenetic analysis with the gene expression pattern, the functional roles of the serpins were predicted and signs of positive selection for the serpin genes in the subclade were detected. Additionally, serpin1 showed six splicing isoforms with different RCLs. The expansion of group A suggests that these serpins are involved in regulating proteolytic cascades in *C. pomonella*. Overall, the genome-wide identification of serpins will advance our understanding of *C. pomonella* immunity and facilitate the elucidation of the role of serpins in physiological activities.

## Figures and Tables

**Table 1:** Summary of serpins identified in the *C. pomonella* genome.

Gene Name	Gene # ^b^ CPOM	Length ^c^	Exon ^d^	Signal Peptide ^e^	pI ^f^	Mw (kDa) ^f^	#chr ^g^	*B. mori* Homolog
*CpSPN1* ^a^	02460	391	9	1–18	5.30	43.132	chr5	BmSPN1
*CpSPN2*	01920	397	8	1–20	4.88	43.697	chr1	BmSPN2
*CpSPN3*	07058	456	7	1–17	5.48	50.788	chr3	BmSPN3
*CpSPN4*	00506	416	1	1–17	5.97	46.694	chr2	BmSPN4
*CpSPN5*	-	395	1	1–15	5.50	44.324	chr23	BmSPN5
*CpSPN6*	18793	439	8	1–17	5.50	49.838	chr1	BmSPN6
*CpSPN7*	00499	373	2	1–15	5.13	42.098	chr2	BmSPN32
*CpSPN8*	-	413	1	1–17	6.28	47.338	chr19	BmSPN4
*CpSPN9*	00026 and 00027	382	3	1–17	5.06	43.463	chr2	BmSPN7
*CpSPN10*	07057	518	7	1–16	5.55	58.325	chr3	BmSPN10
*CpSPN11*	07058	436	6	NO	6.28	48.631	chr3	BmSPN11
*CpSPN12*	07056	570	7	1–18	9.04	63.316	chr3	BmSPN12
*CpSPN13*	14815	432	7	1–19	5.30	49.080	chr3	BmSPN13
*CpSPN14*	00028	390	3	1–16	9.11	44.298	chr2	BmSPN14
*CpSPN15*	09906	371	9	NO	5.96	41.940	chr22	BmSNP2
*CpSPN16*	-	394	1	1–15	6.55	43.996	chr2	BmSPN5
*CpSPN17*	-	418	9	NO	6.47	46.646	chr15	BmSPN2
*CpSPN18*	14126	348	7	NO	5.70	39.096	chr15	BmSPN2
*CpSPN19*	14124 and 14125	391	8	NO	6.98	43.900	chr15	BmSPN2
*CpSPN20*	19028	366	7	NO	5.64	41.271	chr1	BmSPN29
*CpSPN21*	14131	383	8	NO	5.42	43.117	chr15	BmSPN2
*CpSPN22*	02462	276	6	NO	5.41	31.103	chr5	BmSPN1
*CpSPN23*	-	345	7	NO	7.72	38.892	chr15	BmSPN2
*CpSPN24*	-	465	9	NO	5.68	51.965	chr22	BmSPN2
*CpSPN25*	02473	814	16	NO	8.40	89.394	chr5	BmSPN1
*CpSPN26*	12437	1405	6	NO	5.49	155.10	chr16	BmSPN27

^a^ Alternatively spliced isoform A of serpin1; ^b^ *C. pomonella* genome assembly and reference annotations are available at InsectBase (http://www.insect-genome.com/cydia/download.php (accessed on 22 March 2021)); ^c^ Length of amino acid sequence; ^d^ Number of exons; ^e^ Signal peptide prediction; ^f^ Theoretical molecular weight and predicted isoelectric point; ^g^ Chromosome number of gene location.

**Table 2:** The grouping of *C. pomonella* serpin genes based on their phylogenetic relationship and expression pattern.

Serpin ID ^a^	Predicted P1/P1′ Cleavage	Target Protease ^c^	Inhibitory (Yes/No)	#chr	Phylogenetic Group ^d^	Expression Group	
						Stages ^e^	Tissues ^f^
1 ^b^	Y/L	C	Yes	5	A (A2)	I	II
2	M/C	E	Yes	1	A (A1)	II	III
15	I/C	C	Yes	22	A (A1)	III	III
17	Y/Q	C	Yes	15	A (A1)	III	II
18	L/C	C	Yes	15	A (A1)	III	III
19	L/C	C	Yes	15	A (A1)	III	III
21	R/C	T	Yes	15	A (A1)	III	II
22	-	-	No	5	A (A2)	III	I
23	Y/Q	C	Yes	15	A (A1)	III	II
24	M/P	E	Yes	22	A (A1)	III	II
25	V/G	E	Yes	5	A (A2)	III	I
3	K/F	T	Yes	3	B	I	III
4	R/I	T	Yes	2	C (C2)	II	III
5	R/F	T	Yes	23	C (C1)	IV	I
7	R/F	T	Yes	2	C (C4)	IV	I
8	A/N	E	Yes	19	C (C2)	IV	I
9	R/R	T	Yes	2	C (C3)	I	III
14	V/E	E	Yes	2	C (C3)	IV	III
16	R/I	T	Yes	2	C (C1)	II	III
6	R/S	T	Yes	1	D	II	III
10	-	-	No	3	E	II	III
20	-	-	No	1	E	IV	I
26	-	-	No	16	E	IV	I
11	L/I	C	Yes	3	F	II	III
13	R/T	T	Yes	3	F	IV	III
12	L/S	C	Yes	3	G	II	III

^a^ Serpin entries sorted by phylogenetic group (column 6). ^b^ Alternative splicing isoform A of serpin1. ^c^ Trypsin (T)-, chymotrypsin (C)-, or elastase (E)-like specificity. ^d^ Group numbering based on phylogenetic relationship (Figure 3) and subgroup numbering in parenthesis. ^e^ Group numbering based on expression levels across developmental stages (See panel A of the figure in Section 2.7). ^f^ Group numbering based on expression levels across tissues (See panel B of the figure in Section 2.7).

**Table 3:** Test of positive selection on the orthologous/paralogous serpin genes based on the site model.

Clade	n	*d*_N_/*d*_S_	2ΔI	
			M0 vs. M3	M7 vs. M8
Group A1	13	0.19403	477.533 ** (*p* = 0)	66.7717 (*p* = 3.22 × 10^−15^) **
Group A2	7	0.09542	180.71 ** (*p* = 0)	3.99655 (*p* = 0.1356)
Group B	7	0.02043	212.243 ** (*p* = 0)	0.00113 (*p* = 0.9994)
Group C1	7	0.02503	135.335 ** (*p* = 0)	0.70398 (*p* = 0.7033)
Group C2	5	0.13251	165.873 ** (*p* = 0)	0.43196 (*p* = 0.8057)
Group C3	10	0.00763	300.516 ** (*p* = 0)	1.51731 (*p* = 0.4683)
Group C4	4	0.06961	115.716 ** (*p* = 0)	3.60781 (*p* = 0.1647)
Group D	6	0.02532	237.675 ** (*p* = 0)	18.7153 (*p* = 8.63 × 10^−5^) **
Group E	9	0.03745	42.2526 ** (*p* = 6.68 × 10^−10^)	3.99341 (*p* = 0.1358)
Group F	11	0.01565	383.2 ** (*p* = 0)	0.00317 (*p* = 0.9984)
Group G	7	0.12685	210.412 ** (*p* = 0)	20.1964 (*p* = 4.12 × 10^−5^) **
Group H	12	0.35895	72.4922 ** (*p* = 2.22 × 10^−16^)	0.04041 (*p* = 0.9800)
**Clade**	**Parameter Estimated under the M8 Model**			**Positively Selected Sites (PSSs) from Bayes Empirical Bayes (BEB) Analysis**
Group A1	p0 = 0.95322, *p* = 0.80398, *q* = 15.38401, p1 = 0.04678, ω = 24.25724			230G (0.990) **
				232S (1.000) **
				234R (0.990) *
				235S (1.000) **
				239V (0.974) *
				265I (0.947)
Group D	p0 = 0.94870, *p* = 0.97062, *q* = 3.71832, p1 = 0.05130, ω = 10.62317			4C (0.733)
Group G	p0 = 0.97816, *p* = 1.15116, *q* = 6.46773, p1 = 0.02184, ω = 341.91199			3Q (0.985) ** 182S (0.945)

n, Number of genes tested; *d*_N_/*d*_S_, Estimated under M0 model; 2ΔI, Likelihood ratio test. ** Significant within the 1% interval after Bonferroni correction. * Significant within the 5% interval after Bonferroni correction.

## Data Availability

Not applicable.

## Supplementary Materials

The following supporting information can be downloaded at: https://www.mdpi.com/article/10.3390/ijms242216349/s1.
