# Supplementary material for: A Genome-Wide Analysis of Serine Protease Inhibitors in Cydia pomonella Provides Insights into Their Evolution and Expression Pattern
Source: Int J Mol Sci. 2023 Nov 15;24(22):16349. doi: 10.3390/ijms242216349 (PMC10671500; doi:10.3390/ijms242216349)
Supplement: Supplementary file 1 [file ijms-24-16349-s001.zip › Figure S1.pdf]

```
                                                            hA

MsSPN1K : ---------------------------------------------MAGETDLQKILRESNDQFTAQMFSEVVKA--N-------------------------
 CpSPN1 : ---------------------------------MNTLIFFTAVVAMASATGEEKILNDGNNVFTANMFSEVVKE--Q-------------------------
 CpSPN2 : ----------------------------MSTFLLTFLFFIFSTNQISSTNMDSAAVSSAVAKFSMQFCNELDKS----------------------------
 CpSPN3 : -----------------------------AAQPGKPTLGTNETLVDHDYWDSDDYYVQSPADYDSFDWTVTKRIAAY-------------------------
 CpSPN4 : ---------------------------------FGQNQGIKGHDEPEYPTNLHNGLSEATGNFSVELLYHAAKK--Q-------------------------
 CpSPN5 : -------------------------------------MRFTVLILLTSSCYCDVEFSERPRNFSIELLYHTQK---Q-------------------------
 CpSPN6 : ---------------------------------VTGQCFYKDDSAKKLDPEARTSLYRGQLEFTLNLFNSINDA--Y-------------------------
 CpSPN7 : ---------------------------------MWKVIIAACTSLVVTAPVDDRVDFSPINQFALRLLDNAYAFQEN-------------------------
 CpSPN8 : -------------------------------------LGQNHGVKEPVYRMNLAKFSESTGNFTIELLYHVAKR--Q-------------------------
 CpSPN9 : -----------------------------ATYLYLLAIFLPPILGQCSVEKAGHSFSRAVYEFSLDLIQRVGQ---E-------------------------
CpSPN10 : ---------------------------------VLCLAVGLSSARWVRRGRAPPKTTGFVGEATNQLSTAIFQGYID-------------------------
CpSPN11 : ---------------------------------------FLAYLQSLECIDNKRPENARLNYFDTDLLKYTTE---D-------------------------
CpSPN12 : ---------------------------------VVPVSQSSQLLNMTNDIKLASAVISHVGIFMMKSAR-------E-------------------------
CpSPN13 : ---------------------------------AISEHEENTTDPSVTDQPHEQQLSNTINELGFKLLTEEMKR--Y-------------------------
CpSPN14 : ---------------------------------------------NCDERSVTAYYKKPIYEFSTGLLDRVSQ---E-------------------------
CpSPN15 : ---------------------------------------------------MDPKLSLSLWNFAARFCNELEKG----------------------------
CpSPN16 : --------------------------------------MKTIVFLLFVATCYAEEFSARSRNFSIELLHHTQKQ----------------------------
CpSPN17 : -----------------------------------------------------MTSISHVANFGARFCNELDNS----------------------------
CpSPN18 : --------------------------------------------------MVKWGRRNKVVQFLGTLSEMYPEL--D-------------------------
CpSPN19 : ----------------------------------------------------MTLHVNHVANFAARFCNELDSE----------------------------
CpSPN20 : ----------------------------------------------MFRKTLDQVERKFLLSAFNKLTAISPDNRKE-------------------------
CpSPN21 : ------------------------------------------------------MTSIHVANFGARFCNELDKN----------------------------
CpSPN22 : ------------------------------------------------------------------------------------------------------
CpSPN23 : ------------------------------------------------------------------------------------------------------
CpSPN24 : ---------------------------------------------------MEPSLAASLSHFAARFANELEKG----------------------------
CpSPN25 : ---------------------------------TPTTTVKPPTISTGDRGSGLAGVLDGITAFTGKCLYERMQS--A-------------------------
CpSPN26 : DNFVKISTINPHQQNNNPPLKVKETINKVSNPVSKTTVGLETTTSTSTETPMDPILSESMDDLLSQVVNQAPQSIVDSENKLQESNVISTAENFVDTTTVVI
```

s6B hB hC

```
MsSPN1K : ------------------------------------------------------------PG-QNVVLSAFSVLPPLGQLALAS-VGESHDELLRALALP-N
CpSPN1  : ------------------------------------------------------------AG-KSVVMSAFSVLQPLAQLALAS-VGESHDELLRAIGLP-N
CpSPN2  : ---------------------------------------------------------------QNVVSSPLSAEILLALLSLGS-SEPALPELLGAIGFQ-D
CpSPN3  : ------------------------------------------------------------SE-ENFLLSPLGLKLALAILTEAA-TGPTQAQLASVLGFDVN
CpSPN4  : ------------------------------------------------------------GK-QNLILSPITAWTVLAVTSEGA-LGNTRTQLVSALRIKRN
CpSPN5  : ------------------------------------------------------------TG-GHVVISPFGIWTLMTGLAVGA-TGDSFEQIRVAFILPRS
CpSPN6  : ------------------------------------------------------------PT-DNIFFSPFSVYHSLLLAYFSA-GGQTEASLKESLQIE-K
CpSPN7  : ------------------------------------------------------------FGRQNFAISPLSVWSVFSLLAEGS-AGDTFQELMKELQLPQD
CpSPN8  : ------------------------------------------------------------PD-QNFVVSPFAAWTMLAVTYEGA-VGNTNEELILALRFPRK
CpSPN9  : ------------------------------------------------------------ND-NHFVTSPFSLWTVLTTASLGA-EDETLAEMKRVLRLH-H
CpSPN10 : ------------------------------------------------------------DN-KNIAFSPLGYSAILAILAEGA-TGQTREQLVSALHLPEN
CpSPN11 : ------------------------------------------------------------RS-GNVVVSPASIKSTLAMLLEGA-SGNTEVEIRCSLRLSPN
CpSPN12 : ------------------------------------------------------------NH-SNVVVSPTSVVNMLALLQAGA-TGVTQEQVSNALRLS-P
CpSPN13 : ------------------------------------------------------------AD-TNIVMSPTGIAGLLAMVLLGS-AGNTYTELAAALGFSQD
CpSPN14 : ------------------------------------------------------------TD-GHFVCSPINPWLQLVNLAQGA-HGATLKQIWKVTGHGKP
CpSPN15 : ---------------------------------------------------------------KSQVCSPLSAQAVLALTALGS-GGDTRKELLQAMALP-D
CpSPN16 : ------------------------------------------------------------TG-GHVVISPFGIWTLMTGVALGA-TGNSFAELQRAFILPRS
CpSPN17 : ---------------------------------------------------------------KNFICSPLSVETVLALVALGS-SNESRAEIFKALDIP-N
CpSPN18 : ------------------------------------------------------------SN-KNFICSPLSVETVLALVALGS-RNDSREEIFKALDIP-N
CpSPN19 : ---------------------------------------------------------------KTFICSPLSVETVLALVALGS-SNESRAEIFKALDIP-N
CpSPN20 : ------------------------------------------------------------NG-ISFLQSGLFLYHLLMSLSLTM-DQNAVREIEEYFGLNMS
CpSPN21 : ---------------------------------------------------------------KNFICSPLSVETVLALVALGS-SNESRAEIFKALNIP-N
CpSPN22 : -------------------------------------------------------------------MSAMSVFTPIAELCLYTEDGPAFDQCIKILNLK-D
CpSPN23 : ---------------------------------------------------------------MSF-------------------CSAGKQDL---------
CpSPN24 : ---------------------------------------------------------------KSQVCSPLSAQAVLALTALGS-GGDTRKELLEAMALS-D
CpSPN25 : ------------------------------------------------------------PN-ENIIMSAMSVFTPIAQLCLYS-NGPAFDQCIKTLNLK-D
CpSPN26 : KFKTDGNNPESPDKDIVLDVSKENQGLEVTTKDLSDDVAQFTELCNELAFRYWNAIAETIPNKRSFILSPFSITSMLAMMFMGA-RGATSGEMNEILKLD-D
```

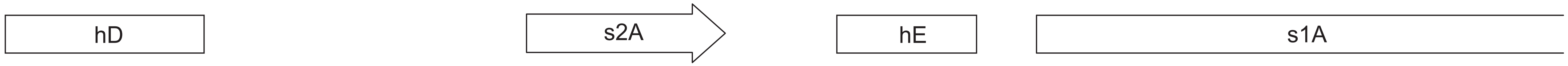


```
MsSPN1K : --DNVTKDVFADLNRGVRA-------------VKGVDLKMASK-IYVAKG-LELNDDFAAVSRDVFGSEV--------------------------------
 CpSPN1  : --DNVTKAVFPKVTAQLRA-------------VQGVKLNMANK-IYIPTN-AQVKEDFAALSKSVFGSEF--------------------------------
 CpSPN2  : --TDAIRSSFSLVSSRLKS-------------VKGVTLDVANR-VYLQQG-HELDPKLKDDAVKVFDAGL--------------------------------
 CpSPN3  : --RTVVRTKFSSIISSLQLA------------NPNYTLNVGSR-IYVDST-VSERQRFSSIAEEFYKTEL--------------------------------
 CpSPN4  : --RTVTRHDFQEIARWLVVN------------TTTVHLAKFNG-IFVDKS-KSLEDDFRQSSKTFYDTQT--------------------------------
 CpSPN5  : --LKTVVCGSKHLSKSVLKRT-----------SDGVSITSKNF-LFADDD-FLLKPAFKQTITEDFDATV--------------------------------
 CpSPN6  : --SLDKINLISAYKVDRRSRLTNNN-------SASYEFNSANK-MFIDET-LDVRQCL----LDIFEDEL--------------------------------
 CpSPN7  : --LRATQELHLAADSVLRSN------------DPDLVIHKQAA-LFPECS-LEIHQEFC-------------------------------------------
 CpSPN8  : SPRTTVRYYYQEIAQWLVVN------------RTTAQFTKFSG-IFMDRVWTGMDNDFRHIAEEIYNTEA--------------------------------
 CpSPN9  : --YPCFNDRYLEIVKGITVY------------NSKTTLERSSV-LFVDKT-VSLLESFHRKLSGTGVCDV--------------------------------
CpSPN10 : --PNLIRKTYRYIMERLKNTHE----------YKYNQPELKNY-FYIYKN-YTINDDYKKILEDYYLTDVRSVERYNDVDHFKPDESNDDDETIEISDKKAA
CpSPN11 : --KNEFREQLNIYLSALQCN------------NTAAKLQNANG-VFVSNK-LILKKEYERVIEKVYLTKV--------------------------------
CpSPN12 : --EKSA-EAFRLMNTELRKRS-----------TERNILRSASS-IFAADS-FELSRDFKNIAIQNFGSEV--------------------------------
CpSPN13 : --ILRNRAHHEQFGSLLQ--------------LLNSSTTYADA-ILVEPR-TRIRPLYREFLQRVYQGDA--------------------------------
CpSPN14 : CMQRTLRKTLNSLY------------------GEGKDYKCQSL-VIIDKY-LAVKNYFKRRVERSKTVKV--------------------------------
CpSPN15 : --DEAIRSLFGYLTSTLNS-------------IQTVKLRFANR-IYVSKH-GTLKPGIQKDSVEVFGSSL--------------------------------
CpSPN16 : --KKTVIAGYKELTKAVLNPS-----------TNGVSLTSKNF-VFIDDD-FTLNPDFRKTISTDFDATI--------------------------------
CpSPN17 : --EDDLFSTFISLTTTLASKPSKHKT------SNDVTLKIANK-IYVKQG-AALKAALKKNAEEVFQASL--------------------------------
CpSPN18 : --EDNLRSVFTSVTQNLKKPFIESLTSKLNPFNRDSTLQIATK-IYVKCG-KSVKPELKKDAEKVFNSSL--------------------------------
CpSPN19 : --EDNLRSVFTSLTQNLKKPFIETITSILKPFSQEYTLKIANK-IYVKQG-KDLKSNFKKDAEKVFNASL--------------------------------
CpSPN20 : --EADKIEILERTVSWLPKS------------SEELKFRWSSR-LVLQR--VAAGNGLHGAAARVLPLRL--------------------------------
CpSPN21 : --EDNLFSTFTSLTATLASKDPSEFKT-----CEDVTLKIANK-IYVKHG-GALKAALKKDAEEVFQASL--------------------------------
CpSPN22 : --KKDIRDTFANFSSLYEG-------------VTSVNFSTAAK-VYSSEK-YELSAGFIKDAIEVFHAAA--------------------------------
CpSPN23 : --RRLLFSTFISLTTTLASKPSKHKT------SNDVTLKIANK-IYVKQG-AALKAALKKNAEEVFQASL--------------------------------
CpSPN24 : --DEALRSLFSYVTSTTKS-------------VEGITLKMANK-VYVTNK-SKLQPDIQKDAEEVFQSAL--------------------------------
CpSPN25 : --KQDIRDTFENFSSLYEG-------------VTSVNFSTAAK-VYSSDK-YQLSDGFKKDAIDVFHAAA--------------------------------
CpSPN26 : MVTFNPHFTLKNISDSIETTP-----------ESGVAISAFIRELYSDRNKGQILTFYKERAQHFYNGHV--------------------------------
```

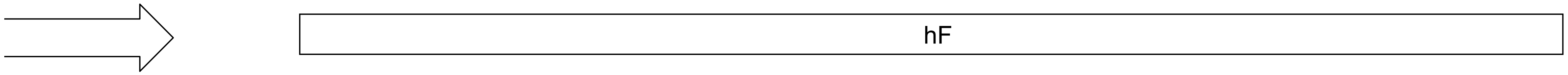


```
MsSPN1K : ------QNVDFVKSVEAA-----------------------------------------------GAINKW-----------------VEDQTNNRIKNLVD
CpSPN1  : ------KNIDFTKNVPAA-----------------------------------------------QEINSW-----------------VEDHTNHKIKDLVD
CpSPN2  : ------EPMDFSNGAVAA-----------------------------------------------KVINDW-----------------VESKTNNKIKDLIS
CpSPN3  : ------KKVDFRLPAETA-----------------------------------------------AQINGW-----------------VSNLTHGNIPNLID
CpSPN4  : ------VDLDFKNSAATA-----------------------------------------------ATINGA-----------------VSTATKGRIPKLVD
CpSPN5  : ------KVLDFKDSNEAA-----------------------------------------------AKANNF------------------IQNSGARVTNVLT
CpSPN6  : ------ERLNFHETPSEA--------------R--------------------------------EHINKW-----------------VAAATKNHIQALIP
CpSPN7  : -----------------------------------------------------------------QAANMY--------------------RTEGQIRNAVK
CpSPN8  : ------VDLPFKNKAAAA----------------------------------------------- TINRA-----------------VSLTTEDRIPTLID
CpSPN9  : ------MILPFDNYNNVA-----------------------------------------------TTINNY-----------------VSQATHNTIQEIVN
CpSPN10 : ESNKNEEIVDLVPPKESG---------------EKFISFAVEDKPEKVDISQIEYKPAKNIKEKIKLVKTYPKKDDSGEEEETMVAVEARNHARSHHHVLQE
CpSPN11 : ------VILNFLDPVGSA-----------------------------------------------AIVNDW-----------------VGNQTRGLIRSLIE
CpSPN12 : ------SRISFDNPNAAA-----------------------------------------------QKINSW-----------------IASKTNDQIDRLVS
CpSPN13 : ------RAVEFTDAPLAR-----------------------------------------------DTVNDW-----------------IKNHTKGKIEEFLK
CpSPN14 : ------ASLDRDTPYDSA-----------------------------------------------DEASEI-----------------IERATG--VGNSID
CpSPN15 : ------QQVDFMEKEGAA-----------------------------------------------DSINKW-----------------VAAKTNNMIRNIVS
CpSPN16 : ------KVLDFKNPDLAA-----------------------------------------------GKANNF------------------IQNSGGRVSNVLT
CpSPN17 : ------EQIDFTQSNNAA-----------------------------------------------NAINKW-----------------VSNQTNNTIKDIVS
CpSPN18 : ------EQIDFVKSSDAA-----------------------------------------------NSINNW-----------------VKKRTNNIIKDLVS
CpSPN19 : ------ELIDFGKNDDAA-----------------------------------------------NSINNW-----------------VAKQTNDIIKDIVS
CpSPN20 : -----SWLVGNETSTQLA-----------------------------------------------ETLNSM-----------------VLSDSGGSMHDTFS
CpSPN21 : ------EQIDFTQSNNAA-----------------------------------------------NAINKW-----------------VSNQTNNTIKDIVS
CpSPN22 : ------QNLDFSKNVEAA-----------------------------------------------AIMNAW-----------------VESQTNNRIKNLVT
CpSPN23 : ------EQIDFTQSNNAA-----------------------------------------------NAINKW-----------------VSNQTNNTIKDIVS
CpSPN24 : ------EQLDFTESAAAAGAVNKWMANKVYVTNKSKLQPDIQKDAEEVFQSALEQLDFTENAAAVGAVNKW-----------------VAEQTEDMIKDVVD
CpSPN25 : ------QEIDFIKKNQAA-----------------------------------------------AIMNAW-----------------VESKTNNRIKDLVS
CpSPN26 : ------EEINFKLISDII--------------R--------------------------------RRTNLL-----------------VKRYTWGKIIEYMK
```

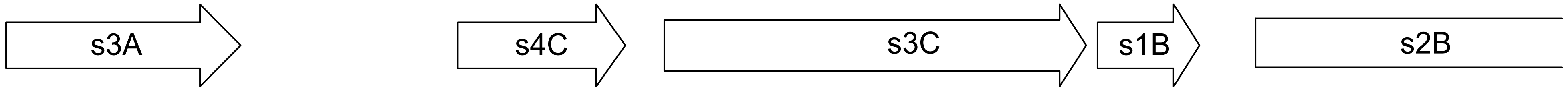


```
MsSPN1K : PDAL-----------------DETTRSVLVNAIYFKGSWKDKFNKERTMDRDFHVSKDKTI-KV----PTMIGKKD-VRYADVPELDAKM------------
 CpSPN1 : PNSL-----------------GADTRAVLVNALYFKGSWEKKFNDKATTDSDFHVTKDKTV-KV----PTMYKKDD-FKYAESKELDAKL------------
 CpSPN2 : EDSL-----------------NALTRLVLVNALYFKGKWKHQFKPHNTMDKPFYVKKDTTV-DV----PMMYIEEQ-FRYGESDKLNAQL------------
 CpSPN3 : EDEV------------------KGSIVLVLTTLFFKGTWKHQFNPNTTKTRAFYVTPTVQK-QV----PFMNVKDK-FYYAESAKYDAKI------------
 CpSPN4 : ESNL------------------SDAQMIITSAVYFKGQWTAPFNVSSTTKQNFYNSSGMKIGEV----NMMYNRYT-YPFANIKEIQARV------------
 CpSPN5 : SEDF------------------EQSRMILTNVITFKGLWTSPFNASHTTLEYFYNENKEIIGEV----NMMHQKGE-FPLSNIIDLKSFV------------
 CpSPN6 : EDGI-----------------THTTKLVLANAAYFKGVWASKFPAERTKKEAFFVSETLQT-LV----PFMKQKGT-FHFMVSEDLGAQI------------
 CpSPN7 : EEFL------------------RDLRLLIVDALYFKANWTHPFDPTATREEDFYNGQGKTIGRV----NMMFHKAS-HNVGDSNSIGAQI------------
 CpSPN8 : ESHL------------------NNTQLIIASAVYFNGQWTAPFNV-ITVKRDFYNSIGEKIGEV----NMMYNRYT-YPFANISELQASV------------
 CpSPN9 : SNDI------------------EGVYLLLLDAVYFKGMWNIPFEPSETEVTSFYDERNNPVGDV----NMMFSMRQ-YNLASIDQLQAKV------------
CpSPN10 : KSDIASSISVNTVGKKSEKTSPSNSLMLIFNGMYFRGAWKQPF--LKVEPAMFYTSNSEKK-QV----QMMCTKGI-FKTGSLPGLDSSA------------
CpSPN11 : PAHI-----------------PPSSDVLVINALYFKSAWQHAFDRSLTRSACFRVQGACKK-QV----AMMEISTE-LNYAFVDNLRAHA------------
CpSPN12 : PDSI-----------------GVNTQLVLANAIYFKGIWETMFKPSETSLQDFNLSNGSKK-SV----PFMTLRSG-FRAGVDKANKALV------------
CpSPN13 : EPL------------------PVETRVVLLSALYFSGQWTHPFLPEHTKKMAFK-TPTGDV-MA----DMMLNFGH-FQYTYSAEDGVQM------------
CpSPN14 : PFEF------------------KLTSLMLADVATFKAAWRQPFDPGFTAVQPFYHGNVKLG-EV----NMMSSIGY-FNYINLTAINAEV------------
CpSPN15 : PNSL-----------------HYATRLLLVNAIYFQGTWEKPFEKDLTKERIFHVNNRDTV-EV----PMMFMDEEYFRYRHSLELKAQL------------
CpSPN16 : SDDF------------------AESRMILTNVISFKGLWLSPFNTSETTVENFYNENNDVVGQV----NMMYQRAE-FPFSNIVELKAFV------------
CpSPN17 : ADML-----------------DEDTRLVLINAIYFHAFWEYQFDADDTYTRVFHVNKSKKI-DI----KQMSLMGKPFNYKHSPELKAKI------------
CpSPN18 : ADML-----------------DGDTGLVLVNAIHFH-----------------------------------------------------I------------
CpSPN19 : ADML-----------------MQDTRVVLVNAIYFHGFWEIPFKANATFTEKFQVNRTKSV-NV----KMMTLEGPSFNYKHSPELQAKI------------
CpSPN20 : EEEV-----------------EAGVCAAALGTVYLRGTWRTA-PTLLNDTLPFRDSPAAPN-RS----SRYFRLNDLMAYSYLHEWDTEA------------
CpSPN21 : ADML-----------------DEDTRLVLINAIYFHAFWEYRFDADDTCTRVFHVNNSKKI-DI----KQMSLMGKPFNYKHSPELKAKI------------
CpSPN22 : PDMI-----------------SALTRLVLINAIYFKGNWLYRFNETNTKEEDFYLSDGSTA-KV----KLMYQEINDLEYGEVPSLDCKV------------
CpSPN23 : ADML-----------------DEDTRLVLINAIYFHAFWEYQFDADDTYTRVFHVNKSKKI-DI----KQMSLMGKPFNYKHSPELKAKI------------
CpSPN24 : AKSL-----------------NSDTRLLLINAIYFQGFWETPFRTRATRDRPFHVDLNTQI-KV----PMMSMHDVHFRYKHSPELKAQLIVKKILGLVFDI
CpSPN25 : PDML-----------------SESTRIVLINAIYFKGKWLKQFNENDTKKEDFYLSDGSTT-KT----KLMYQEIKDLEYGEDASLDCKV------------
CpSPN26 : ANTI-----------------TMQPPLAAFSTNIFETDCTGSSVEGRDGEMYFVVSPNVRQRRLVPVPAVLYKKG--FLAGYDPVLDATA------------
```

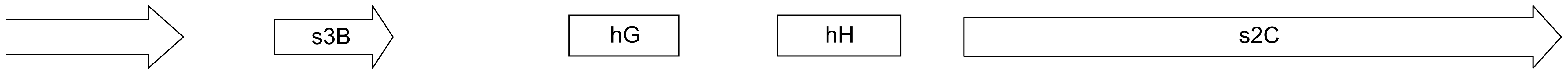


```
MsSPN1K : ---------IEMSY-EGDQASMIIILP--NQVDG----ITALEQKL-KDPKA---LSRAEERLYNT-----------------------------EVEIYLPK
 CpSPN1 : ---------LELPY-EGKEASFLIVLP--NEIDG----LPALQEKL-KNPTA---LDKAVAEMREV-----------------------------EVNVYLPK
 CpSPN2 : ---------LEMPY-EGDEASMVIVLP--KDIEG----LDTVLKQL-ASGVD---LVQELNNMIST-----------------------------KVQVTVPK
 CpSPN3 : ---------LRMPY-LGKKFSMYIVVP--NSLKG----LPRVMDSL----SD---LRTEMLYLAER-----------------------------TVDVTLPK
 CpSPN4 : ---------IELPYGVENRLSMLLMLP--NPGVT----LEDMFLNF--KKVP---LDTVFQELKLS----------------------QEEYSDDEIDCFIPR
 CpSPN5 : ---------CELTYGTDAAYSMLVILP--HPGVK----VEDVYKKF--ENIT---IPDIIARLQND----------------------EKEYGLDEVEIKLPR
 CpSPN6 : ---------LELPY-KGNDISMYILLPPYSMKED----MTNRCKPY--GEFA---WPYSMQNLLVSDFHRCEEHHSQPDTPARLAAVVEESYMGREVIVEIPK
 CpSPN7 : ---------LEMTYGKHEEFSMLILLP--FEGMP----LKTLLSNLVTSPLD---WITDFKRDDKR----------------------------PEIDVFIPR
 CpSPN8 : ---------IELPYGVENHMSMILILP--NPGMT----LKDMFLIF--LKAN---LKTVFQELKVS----------------------QEERGDVEIDCFIPR
 CpSPN9 : ---------LELPYGDGDRFSMLIFLP--YQSSS----VAKVTDQL--KRVT---LKSIFYIFQRN--------------------------GRDTVQVQIPS
CpSPN10 : ---------IELPY-DGGRYSLLIVLP--RTRDG----LTRLTADL--PAAP---LEDIQDSLREE-----------------------------ELQVSLPT
CpSPN11 : ---------VELPY-EGGRYSMVLLVP--QDHDG----LAGLIRDL--PYMS---LPQICKMMEPS-----------------------------HVQLSLPR
CpSPN12 : ---------IVLPF-ENNEYSLVMLLP--VPGAD----VSAVLTSL---DGE---TLANYQNLRRQ-----------------------------DVLLEIPK
CpSPN13 : ---------IALPY-KDNTTTMYVLKP--LKKLS----LQDLMSRL--NYSR---IDQLISNMTND-----------------------------RAVIRFPK
CpSPN14 : ---------LELPC-IDKRRSMLIFLPTNGTIKD----LFFHMKRI--RIAT---IFNLFKKSKSK-----------------------------LVVLRLPR
CpSPN15 : ---------LQMNY-KGRQASMILIVP--DAVEG----LGAMLQQL--SDHD---LMDEVAVMRAT-----------------------------RIEVMIPK
CpSPN16 : ---------VELPYGNDGKYAMLLILP--HPRVK----LDDVYRRL--ANVT---LTEITKKLQSD----------------------IDEYGMTDVDIKLPR
CpSPN17 : ---------LQMFY-VGGEASMVIVLP--DEIEG----LSAVMKQL-ATGYD---LMAEIKSLQKK-----------------------------QLQVIIPK
CpSPN18 : ---------LQMPY-VGGEASMVIILP--DEIEGDTGSVTDLVKRL-AAGFD---LMSEIKRLKKT-----------------------------DLQVKIPK
CpSPN19 : ---------LQMFY-KGGQASMVIVLP--DKIEGETAGVGGLMERL-IAGYD---LMADIKSLQKT-----------------------------PLKVIIPK
CpSPN20 : ---------IEIPY-ATPGLTLLILVP--KELS-----LRPLVERA--AAVG---LDAVVGVMHTM-----------------------------RIAVTLPL
CpSPN21 : ---------LQMFY-VGGEASMVIVLP--DEIEG----LSAVMKQL-AAGYD---LMTEIRSLQKK-----------------------------ELEVIIPK
CpSPN22 : ---------VRILY-TGKESSAIIILP--NSING----TMQLAKDL-QNTAN---WKKVIDSLYPQ-----------------------------KVKLYLPK
CpSPN23 : ---------LQMFY-VGGEASMVIVLP--DEIEG----LSAVMKQL-ATGYD---LMAEIKSLQKK-----------------------------QLQVIIPK
CpSPN24 : RIHFPFQFLQMDY-KGRQASMVIVLP--DEIEG----LGAVLQQL-AAGHD---LMAELTDMKST-----------------------------LLDVMVPK
CpSPN25 : ---------VRIPY-TGEESSAIIVLP--NSING----TMHLAQNL-QFPAN---WKKLLDSLSPQ-----------------------------KVQLYLPK
CpSPN26 : ---------AALGN-TNSIISTLFLMPGQQGNVVHAEDLELLEKRLLGSNPSTPAWNRLLRTLLPR----------------------------IGLELQIPR
```

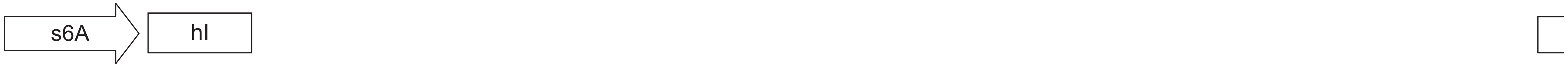


```
MsSPN1K : FKIETTT-DLKEVLSN--------------------------MNIKKLFTPGAA-------------------------------RLENLLKTKESLYVDAA
CpSPN1  : FKIETTI-DLKKVLQK--------------------------IGITSLFDAAKA-------------------------------RLDNLLKNESGLYVSDA
CpSPN2  : FKIETEI-DLKELLPK--------------------------LGIKAIFDEKNS-------------------------------GLTKILDSTEPLYVSKA
CpSPN3  : FKFDFTS-ILDGVLKD--------------------------LGIRQAFEDTASFP-----------------------------GLARGQSPQNRLKISKV
CpSPN4  : FKIDSSI-ELNEILKEG-------------------------MGIYDLFDPVKA-------------------------------NLGRL--ARVPMYVTRV
CpSPN5  : FKISTNI-VMNRPLND--------------------------MGVYDIFDPEYA-------------------------------SFSRA--TNESIYVSAI
CpSPN6  : FTVEKSL-PLRPILQS--------------------------LGVGDLFNSTSD--------------------------------FTTL--TEDQVSFDDA
CpSPN7  : FKISSQI-DLIPALKY--------------------------TGIHTIFDSEKA-------------------------------QLPGI--SDSPLFVSKT
CpSPN8  : FKIVSNI-ELNEILKNG-------------------------MGIHDLFDPKNV-------------------------------FLPRM--SRWPIRGTRV
CpSPN9  : AQFPHI--------------------------------------------------------------------------------------SDYSFYISNV
CpSPN10 : FYVETTT-KPVAALAK--------------------------FGVSSIFSRDAE--------------------------------LSGVS-SAEGLFVQEL
CpSPN11 : FTVDYSE-DMVGPLRS--------------------------MRITTLFSSKSN-------------------------------LSGII--EGGPAQINHL
CpSPN12 : FTVRSNA-NLQPIFAK--------------------------MGITKMFSPTSE-------------------------------LSHIGLYRANSPQVSSA
CpSPN13 : MDLKNSI-QLEDSLKS--------------------------IGLSEMFSPTANFALMIDTDRDKSNEELLTRIIFEDKSQHIQDAVNGL--PNPGVHVDKV
CpSPN14 : FKISSEIVNLPELISD--------------------------MGVSYIFKINGN--------------------------------LKGI--SDFPIRVNRM
CpSPN15 : FKIETDI-NLEELLPK--------------------------MGIHDIFDQNKS-------------------------------DIRMFT-SHDRMCVTQA
CpSPN16 : FKISTNV-VMNKPLND--------------------------MGVYDIFQPDVA-------------------------------SFKRV--TNENIYVSAI
CpSPN17 : FKIESEI-NLKELLPKGTSPTRKVGVNIFFCCNPTVWGIVGKMGIKAIFDPIKS-------------------------------EVNMLS-SDDRQFVSQA
CpSPN18 : FKVETEI-NLRELLPK--------------------------MGIQTIFDKNKA-------------------------------EINMLS-SNDPLHVSEA
CpSPN19 : FKAETEI-NLKELLPK--------------------------MGIKAVFDMNKG-------------------------------DTTMLS-SDEPLYVSEA
CpSPN20 : YTLRMTL-LLPNKLQD--------------------------MGMQSLLTNGTD---------------------------------------CQPVRLSHA
CpSPN21 : FKIESEI-NLKELLPK--------------------------MGIKTIFDPIKS-------------------------------EVNMLS-SDDRQFVSQA
CpSPN22 : FVISTTT-DLKVLLQK--------------------------SEV---------------------------------------------------------
CpSPN23 : FKIESEI-NLKELLPK--------------------------MGIKAIFDPIKS-------------------------------EVNMLS-SDDRQFVSQA
CpSPN24 : FKIETEI-DLTQLLPE--------------------------MGIKLIFNDRYS-------------------------------NINMLT-SNEPLYVSKA
CpSPN25 : FVISTTT-DLKDLLKK--------------------------ANITNWFNCGNS-------------------------------GLSGLLAKPEDICITEA
CpSPN26 : FSHKSIF-NVSSTLKK--------------------------MGLKDLFDAEHA-------------------------------DLGGLNGPSKDLYLSDM
```

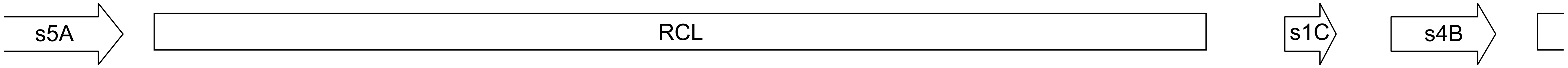


```
MsSPN1K : IQKAFIEVNEEGAEAAA--------------------------------------ANAF-------------KITTYSFHFVPKVEINKPFFFSLKYNRNS-
 CpSPN1 : IQKAFIEVNEEGAEAAA--------------------------------------ANDF-----GVTY------LSAYVKPETYFNADHPFIFILKSGESA-
 CpSPN2 : IQKAFIEVNEEGAEAAA--------------------------------------ATAM-----NIMM----CCAMVDVAPVPVFTADRPFLAVIQAHGIL-
 CpSPN3 : LQRSGIEVNELGSVAYS--------------------------------------VTEV-----SLVN-----KFGEDEYSAGEVIANRPFLFFIEDEATRQ
 CpSPN4 : IHKAEIEVTEEGTTASG--------------------------------------ATAA---------------EFANRIGVIRFEANRPFSYLIVEKVTNT
 CpSPN5 : VHKADIEVSETGTVASA--------------------------------------VSTA---------------YLSNRFGTTKLIADRPFLYFIIEKSTAT
 CpSPN6 : VHKAKIQIDEEGTVAAA--------------------------------------ATAI------------FGFRSSRPAEPTRFVANFPFVYLVYERPTNS
 CpSPN7 : IQNVEIDVKEEGTVAAA--------------------------------------ATVV---------------GLENRFLSQRFEANKEFVFMITHRKSNV
 CpSPN8 : IHKTEIDVTEESSTPSG--------------------------------------NIAP---------------EFANDSKVLIFEANRPFSYFIVEKHTDT
 CpSPN9 : VQKADVEVTEEGTVAAA--------------------------------------ASAA---------------EFEARRLPDSFQANRPFVFMIVDKLNYI
CpSPN10 : VQHVAVRVDNADTSASQ--------------------------------------LSAI-----NAVQESLKNIPLSSIKEPRRFSVDRPFMFFIIDRLDNL
CpSPN11 : YHRVYVSVDEDGTVAAA--------------------------------------ASAA--------------LVVPLIEGNVQLVVDKPFAFFIKDNTLGL
CpSPN12 : VHSAMMSIDERGATAAA--------------------------------------STVV------------AVVALSFDNPSVVFRADRPFLAVLWDNQLNV
CpSPN13 : LHEVRITIDEYGTEAVA--------------------------------------ASGG-----------------FLSRTAEQFYVDSPFYMFIRDESTKL
CpSPN14 : KQVVEIEVTEEGVAADI--------------------------------------VQNL----------------IDPEVEPMKFEANRPFAFMLVDKPTEI
CpSPN15 : IQRAVIEXNETGTEAAA--------------------------------------CTLT-----AISE----ICAVIELEPPKRFVADRPFLYLLLARGVP-
CpSPN16 : VHKADIEVTETGTVASA--------------------------------------STSA---------------NFADRISTPLMRANKPFAYFIIEKTTTT
CpSPN17 : IQKAFIAVDELGTEAAA--------------------------------------ASLLTTWPYQRKRDSTTLSCIGLILYVDSVDVDIRHF---IEHGHSC
CpSPN18 : IQKAFITVDEKGTEAAA--------------------------------------ANEI-----------TLQLLCLRWWMGETFIADHPFLYILLAQEIP-
CpSPN19 : IQKAFISVDEAGTEAAA--------------------------------------ATQM-----VRRR------LCGHWWTGETFVANRPFLYQLTAREIP-
CpSPN20 : VQRLMF-WAEAGRNAFK--------------------------------------DDGI----------------EWDPRPELEIVVDRPYLFFVRWNNVT-
CpSPN21 : IQKAFIAVDELGTEAAA--------------------------------------ATSM-----------WQRERCGHFWTGDTFTADRPFLFLLQAHEIP-
CpSPN22 : ---------------------------------------------------------------------------------------KLMFYVIMCVVT---
CpSPN23 : IQKAFIAVDELGTEAAA-------------------------------------ASLLTTWPYQRKRDSTTLSCIGLILYVDSVDVDIRHF---IEHGHS--
CpSPN24 : VQKALIEVDEAGTKAVA--------------------------------------RTMI------------PPEMPCCIRRPRKFKADRPFVYLLAAREFP-
CpSPN25 : IQKAWCEFNEIGTEATA--------------------------------------ANVV------------VGVGGAMPMPPVVFKANRSFLFYILLKNASE
CpSPN26 : VQATTFVTCGEGIIGEQHHIEEYPESIELARRRRTSRWATGWDEPRDYQRAFHDPHDVGDAMNLPLHLRPRQARLPARNAQPARLKFDRPFLYFVRHNPSGM
```

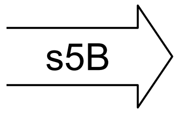


```
MsSPN1K : -MFSGVCVQP------------------------------
CpSPN1  : -LFSGVFYN-------------------------------
CpSPN2  : -YFLAVHSGNE-----------------------------
CpSPN3  : LLFTGRVSDPLVATAEFKAHTPSA----------------
CpSPN4  : IVFGGVYQQPILY---------------------------
CpSPN5  : VIFAGIYSKPSLY---------------------------
CpSPN6  : VLFMGVYRDPKK----------------------------
CpSPN7  : ILFAGVYSDPAVV---------------------------
CpSPN8  : VVLAGVYRKPILY---------------------------
CpSPN9  : PLFTEEDGTERSRLRDSLTGFR------------------
CpSPN10 : VVIAGKVIDPEAPTPFEI----------------------
CpSPN11 : VLFEGKIEEPHEYVAPKKGSENNFVKSSRNSFGTRF----
CpSPN12 : PLFMTKIEDPSL----------------------------
CpSPN13 : VTFSAAIFNPNL----------------------------
CpSPN14 : ILFAGVYSIPSVF---------------------------
CpSPN15 : -TFIGVYHQ-------------------------------
CpSPN16 : VIFGGIYSKPTVY---------------------------
CpSPN17 : ----GLPEHPHFHHMNSVQL--------------------
CpSPN18 : -IFYGIFHGNETSL--------------------------
CpSPN19 : -IFYGTFYGNGYSQ--------------------------
CpSPN20 : -IMNGVFVL-------------------------------
CpSPN21 : -VFYGTFNGSDIIFFKY-----------------------
CpSPN22 : ----LATDS-------------------------------
CpSPN23 : ---CGLPEHPHFHHMNSVQL--------------------
CpSPN24 : -IFLGIYQGSEFAVPDVSDSDIDQTKPVNVGVKKQLSK--
CpSPN25 : LPIFCGKYGNPN----------------------------
CpSPN26 : ILYVGRYNPRLLP---------------------------
```

Shutter Breach Hinge Gate

Figure S1 Multiple sequence alignment of 26 *Cydia pomonella* (CpSPN1-26) with *Manduca sexta* serpin-1K (MsSPN1K). Conserved residues in regions, which are critical for the inhibition mechanism, are highlighted based on the amino acid residues of MsSPN1K. Residues corresponding to the shutter are in cyan, residues of the breach in yellow, residues conserved in the hinge region are in green, and residues of the gate region are in magenta. Predicted α-helix and β-strand and reactive center loop (RCL) secondary structures of serpins are assigned based on the crystal structure of MsSPN1K.
