# Supplementary material for: A Genome-Wide Analysis of Serine Protease Inhibitors in Cydia pomonella Provides Insights into Their Evolution and Expression Pattern
Source: Int J Mol Sci. 2023 Nov 15;24(22):16349. doi: 10.3390/ijms242216349 (PMC10671500; doi:10.3390/ijms242216349)
Supplement: Supplementary file 1 [file ijms-24-16349-s001.zip › Figure S2.pdf]

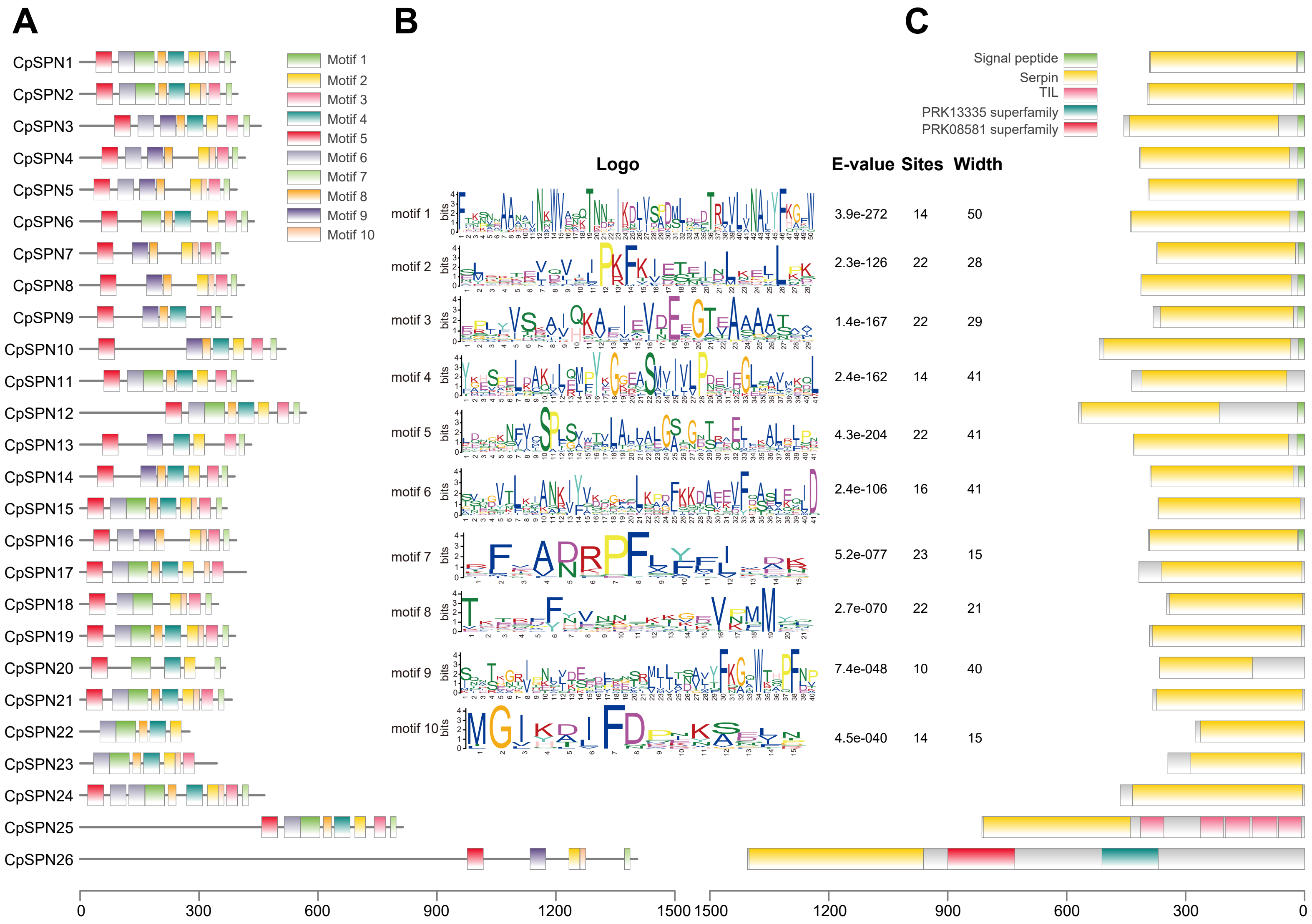

Figure S2 Conserved motifs and domain structure of serpin genes in the codling moth. **(A)** Distribution of conserved motifs in 26 codling moth serpins. The distribution plot of conserved motifs was created using TBtools-II (https://github.com/CJ-Chen/TBtools-II). **(B)** Logos of ten conserved motifs identified within codling moth serpins. Conserved motifs were predicted using MEME suite 5.5.4 (https://meme-suite.org/meme/tools/meme). **(C)** Domain structure of 26 serpin genes in the codling moth. Conserved domains were predicted using the online CD-search tool (https://www.ncbi.nlm.nih.gov/Structure/cdd/wrpsb.cgi).
