# Supplementary material for: A Genome-Wide Analysis of Serine Protease Inhibitors in Cydia pomonella Provides Insights into Their Evolution and Expression Pattern
Source: Int J Mol Sci. 2023 Nov 15;24(22):16349. doi: 10.3390/ijms242216349 (PMC10671500; doi:10.3390/ijms242216349)
Supplement: Supplementary file 1 [file ijms-24-16349-s001.zip › Figure S3.pdf]

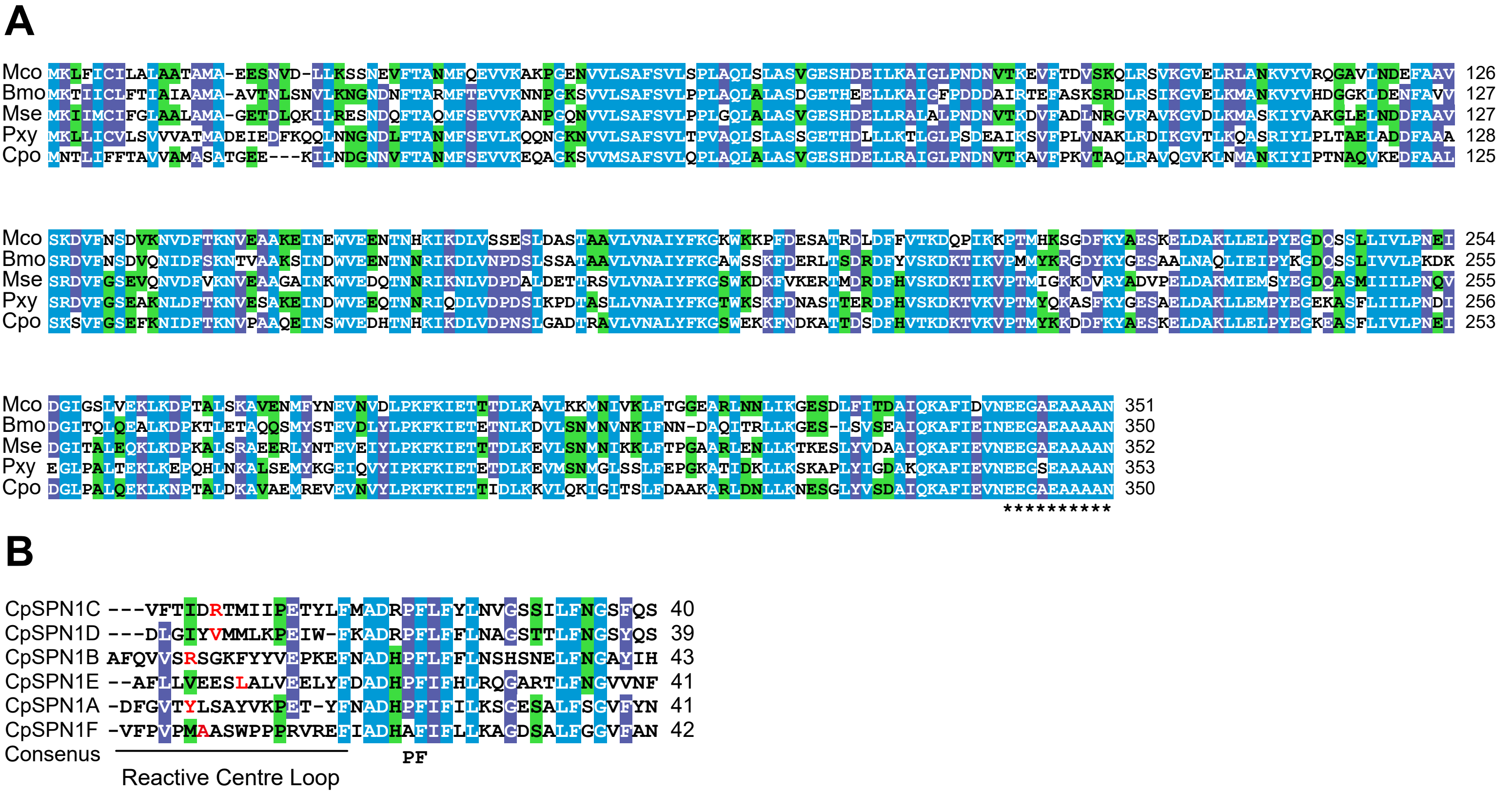

Figure S3 Comparison of protein sequences encoded by *serpin1* in several lepidopteran insects. **(A)** Sequence alignment of conserved region of serpin1 proteins in *Mamestra configurata*, *Bombyx mori*, *Manduca sexta*, *Plutella xylostella*, and *Cydia pomonella*. The hinge region preceding the reactive center loop (RCL) showed almost identical residues (in blue background) and similar residues (in green or purple background) were also indicated. **(B)** Alignment of *C. pomonella* serpin1 carboxyl-terminal inhibitor domain variants following the hinge region with the RCL and P1 positions are highlighted in red.
