# Supplementary material for: A Genome-Wide Analysis of Serine Protease Inhibitors in Cydia pomonella Provides Insights into Their Evolution and Expression Pattern
Source: Int J Mol Sci. 2023 Nov 15;24(22):16349. doi: 10.3390/ijms242216349 (PMC10671500; doi:10.3390/ijms242216349)
Supplement: Supplementary file 1 [file ijms-24-16349-s001.zip › Table S1.pdf]

Table S1. Complete sequences of *Cydia pomonella* serpins

Gene name	chr number	CDS sequence	Protein sequence*
CpSPN1A	chr5	ATGAATACACTTATATTCTTTACAGCCGTCGTGGCCATGGCTTCAGCCACTGGTGAGGAGAAGATCCTCAATGATGGAAACAACGTGTTTACTGCCAACATGTTTTCGGAGGTGGTAAAA<br>GAGCAGGCCGGCAAGAGCGTGGTGATGTCCGCCTTCTCTGTCCTGCAGCCGCTGGCTCAACTGGCCCTGGCCTCTGTCGGCGAGTCCCATGACGAGCTCCTCAGAGCCATCGGCCTTCC<br>CAACGACAATGTGACCAAAGCAGTGTTCCCCAAAGTGACCGCTCAACTTCGAGCAGTACAAGGAGTGAAACTGAACATGGCCAACAAAATATACATTCCCACAAACGCTCAAGTTAAGG<br>AAGACTTTGCCGCACTCTCAAAGAGCGTCTTCGGTTCAGAATTCAAGAACATTGACTTTACGAAAAATGTGCCTGCTGCTCAGGAAATCAACTCATGGGTGGAGGATCACACAAACCAC<br>AAAATCAAGGACTTGGTCGACCCGAACTCCCTGGGAGCTGATACTCGTGCGGTCCTCGTCAATGCTCTGTACTTCAAGGGTTCGTGGGAAAAAAAATTCAATGATAAGGCTACTACTGAT<br>AGTGATTTCCATGTAACTAAAGACAAGACAGTGAAAGTGCCGACGATGTACAAGAAAGACGACTTTAAATACGCCGAGAGCAAAGAGCTCGATGCTAAGCTCTTGGAACTGCCATATGA<br>AGGAAAGGAGGCATCTTTCCTTATCGTCCTCCCCAACGAAATCGACGGTCTGCCAGCTTTGCAGGAGAAACTGAAGAACCCCACTGCCTTAGACAAAGCTGTTGCCGAGATGCGCGAG<br>GTGGAAGTCAACGTCTACCTCCCTAAGTTCAAGATTGAAACTACTATCGACCTGAAGAAAGTTCTGCAAAAGATCGGCATTACCAGCCTTTTCGACGCCGCCAAGGCCAGATTGGACAAT<br>CTCTTGAAGAACGAAAGCGGTCTGTACGTCAGTGATGCCATCCAGAAGGCTTTCATTGAAGTCAACGAGGAGGGTGCCGAGGCAGCTGCAGCTAATGATTTCGGTGTAACGTACCTGA<br>GCGCATACGTTAAGCCCGAAACCTACTTCAACGCTGACCATCCATTCATCTTCATTCTTAAGTCGGGAGAAAGCGCGTTGTTCAGTGGCGTTTTCTATAATTAG	MNTLIFFTAVVAMASATGEEKILNDGNNVFTANMFSEVVK<br>EQAGKSVVMSAFSVLQPLAQLALASVGESHDELLRAIGLPN<br>DNVTKAVFPKVTAQLRAVQGVKLNMANKIYIPTNAQVKED<br>FAALSKSVFGSEFKNIDFTKNVPAAQEINSWVEDHTNHKIK<br>DLVDPNSLGADTRAVLVNALYFKGSWEKKFNDKATTDSDF<br>HVTKDKTVKVPTMYKKDDFKYAESKELDAKLLELPYEGKEA<br>SFLIVLPNEIDGLPALQEKLKNPTALDKAVAEMREVEVNVYL<br>PKFKIETTIDLKKVLQKIGITSLFDAAKARLDNLLKNESGLYVS<br>DAIQKAFIEVNEEGAEAAAANDFGVTYLSAYVKPETYFNAD<br>HPFIFILKSGESALFSGVFYN
CpSPN1B	chr5	ATGAATACACTTATATTCTTTACAGCCGTCGTGGCCATGGCTTCAGCCACTGGTGAGGAGAAGATCCTCAATGATGGAAACAACGTGTTTACTGCCAACATGTTTTCGGAGGTGGTAAAA<br>GAGCAGGCCGGCAAGAGCGTGGTGATGTCCGCCTTCTCTGTCCTGCAGCCGCTGGCTCAACTGGCCCTGGCCTCTGTCGGCGAGTCCCATGACGAGCTCCTCAGAGCCATCGGCCTTCC<br>CAACGACAATGTGACCAAAGCAGTGTTCCCCAAAGTGACCGCTCAACTTCGAGCAGTACAAGGAGTGAAACTGAACATGGCCAACAAAATATACATTCCCACAAACGCTCAAGTTAAGG<br>AAGACTTTGCCGCACTCTCAAAGAGCGTCTTCGGTTCAGAATTCAAGAACATTGACTTTACGAAAAATGTGCCTGCTGCTCAGGAAATCAACTCATGGGTGGAGGATCACACAAACCAC<br>AAAATCAAGGACTTGGTCGACCCGAACTCCCTGGGAGCTGATACTCGTGCGGTCCTCGTCAATGCTCTGTACTTCAAGGGTTCGTGGGAAAAAAAATTCAATGATAAGGCTACTACTGAT<br>AGTGATTTCCATGTAACTAAAGACAAGACAGTGAAAGTGCCGACGATGTACAAGAAAGACGACTTTAAATACGCCGAGAGCAAAGAGCTCGATGCTAAGCTCTTGGAACTGCCATATGA<br>AGGAAAGGAGGCATCTTTCCTTATCGTCCTCCCCAACGAAATCGACGGTCTGCCAGCTTTGCAGGAGAAACTGAAGAACCCCACTGCCTTAGACAAAGCTGTTGCCGAGATGCGCGAG<br>GTGGAAGTCAACGTCTACCTCCCTAAGTTCAAGATTGAAACTACTATCGACCTGAAGAAAGTTCTGCAAAAGATCGGCATTACCAGCCTTTTCGACGCCGCCAAGGCCAGATTGGACAAT<br>CTCTTGAAGAACGAAAGCGGTCTGTACGTCAGTGATGCCATCCAGAAGGCTTTCATTGAAGTCAACGAGGAGGGTGCCGAGGCAGCTGCAGCTAATGCATTCCAAGTTGTAAGCCGAT<br>CAGGCAAATTCTACTACGTTGAACCAAAAGAATTTAACGCAGACCATCCTTTCCTCTTCTTCTTAAATAGTCACTCAAACGAGCTTTTCAACGGAGCTTATATTCATTAA	MNTLIFFTAVVAMASATGEEKILNDGNNVFTANMFSEVVK<br>EQAGKSVVMSAFSVLQPLAQLALASVGESHDELLRAIGLPN<br>DNVTKAVFPKVTAQLRAVQGVKLNMANKIYIPTNAQVKED<br>FAALSKSVFGSEFKNIDFTKNVPAAQEINSWVEDHTNHKIK<br>DLVDPNSLGADTRAVLVNALYFKGSWEKKFNDKATTDSDF<br>HVTKDKTVKVPTMYKKDDFKYAESKELDAKLLELPYEGKEA<br>SFLIVLPNEIDGLPALQEKLKNPTALDKAVAEMREVEVNVYL<br>PKFKIETTIDLKKVLQKIGITSLFDAAKARLDNLLKNESGLYVS<br>DAIQKAFIEVNEEGAEAAAANAFQVVSRSGKFYYVEPKEFN<br>ADHPFLFFLNSHSNELFNGAYIH
CpSPN1C	chr5	ATGAATACACTTATATTCTTTACAGCCGTCGTGGCCATGGCTTCAGCCACTGGTGAGGAGAAGATCCTCAATGATGGAAACAACGTGTTTACTGCCAACATGTTTTCGGAGGTGGTAAAA<br>GAGCAGGCCGGCAAGAGCGTGGTGATGTCCGCCTTCTCTGTCCTGCAGCCGCTGGCTCAACTGGCCCTGGCCTCTGTCGGCGAGTCCCATGACGAGCTCCTCAGAGCCATCGGCCTTCC<br>CAACGACAATGTGACCAAAGCAGTGTTCCCCAAAGTGACCGCTCAACTTCGAGCAGTACAAGGAGTGAAACTGAACATGGCCAACAAAATATACATTCCCACAAACGCTCAAGTTAAGG<br>AAGACTTTGCCGCACTCTCAAAGAGCGTCTTCGGTTCAGAATTCAAGAACATTGACTTTACGAAAAATGTGCCTGCTGCTCAGGAAATCAACTCATGGGTGGAGGATCACACAAACCAC<br>AAAATCAAGGACTTGGTCGACCCGAACTCCCTGGGAGCTGATACTCGTGCGGTCCTCGTCAATGCTCTGTACTTCAAGGGTTCGTGGGAAAAAAAATTCAATGATAAGGCTACTACTGAT<br>AGTGATTTCCATGTAACTAAAGACAAGACAGTGAAAGTGCCGACGATGTACAAGAAAGACGACTTTAAATACGCCGAGAGCAAAGAGCTCGATGCTAAGCTCTTGGAACTGCCATATGA<br>AGGAAAGGAGGCATCTTTCCTTATCGTCCTCCCCAACGAAATCGACGGTCTGCCAGCTTTGCAGGAGAAACTGAAGAACCCCACTGCCTTAGACAAAGCTGTTGCCGAGATGCGCGAG<br>GTGGAAGTCAACGTCTACCTCCCTAAGTTCAAGATTGAAACTACTATCGACCTGAAGAAAGTTCTGCAAAAGATCGGCATTACCAGCCTTTTCGACGCCGCCAAGGCCAGATTGGACAAT<br>CTCTTGAAGAACGAAAGCGGTCTGTACGTCAGTGATGCCATCCAGAAGGCTTTCATTGAAGTCAACGAGGAGGGTGCCGAGGCAGCTGCAGCTAATGTGTTCACCATTGATAGAACGAT<br>GATAATTCCGGAAACATACTTGTTTATGGCAGACCGCCCGTTCTTGTTCTACCTTAACGTCGGCTCCAGCATTTTATTCAACGGCTCCTTCCAGTCCTAA	MNTLIFFTAVVAMASATGEEKILNDGNNVFTANMFSEVVK<br>EQAGKSVVMSAFSVLQPLAQLALASVGESHDELLRAIGLPN<br>DNVTKAVFPKVTAQLRAVQGVKLNMANKIYIPTNAQVKED<br>FAALSKSVFGSEFKNIDFTKNVPAAQEINSWVEDHTNHKIK<br>DLVDPNSLGADTRAVLVNALYFKGSWEKKFNDKATTDSDF<br>HVTKDKTVKVPTMYKKDDFKYAESKELDAKLLELPYEGKEA<br>SFLIVLPNEIDGLPALQEKLKNPTALDKAVAEMREVEVNVYL<br>PKFKIETTIDLKKVLQKIGITSLFDAAKARLDNLLKNESGLYVS<br>DAIQKAFIEVNEEGAEAAAANVFTIDRTMIIPETYLFMADR<br>PFLFYLNVGSSILFNGSFQS
CpSPN1D	chr5	ATGAATACACTTATATTCTTTACAGCCGTCGTGGCCATGGCTTCAGCCACTGGTGAGGAGAAGATCCTCAATGATGGAAACAACGTGTTTACTGCCAACATGTTTTCGGAGGTGGTAAAA<br>GAGCAGGCCGGCAAGAGCGTGGTGATGTCCGCCTTCTCTGTCCTGCAGCCGCTGGCTCAACTGGCCCTGGCCTCTGTCGGCGAGTCCCATGACGAGCTCCTCAGAGCCATCGGCCTTCC<br>CAACGACAATGTGACCAAAGCAGTGTTCCCCAAAGTGACCGCTCAACTTCGAGCAGTACAAGGAGTGAAACTGAACATGGCCAACAAAATATACATTCCCACAAACGCTCAAGTTAAGG<br>AAGACTTTGCCGCACTCTCAAAGAGCGTCTTCGGTTCAGAATTCAAGAACATTGACTTTACGAAAAATGTGCCTGCTGCTCAGGAAATCAACTCATGGGTGGAGGATCACACAAACCAC<br>AAAATCAAGGACTTGGTCGACCCGAACTCCCTGGGAGCTGATACTCGTGCGGTCCTCGTCAATGCTCTGTACTTCAAGGGTTCGTGGGAAAAAAAATTCAATGATAAGGCTACTACTGAT<br>AGTGATTTCCATGTAACTAAAGACAAGACAGTGAAAGTGCCGACGATGTACAAGAAAGACGACTTTAAATACGCCGAGAGCAAAGAGCTCGATGCTAAGCTCTTGGAACTGCCATATGA<br>AGGAAAGGAGGCATCTTTCCTTATCGTCCTCCCCAACGAAATCGACGGTCTGCCAGCTTTGCAGGAGAAACTGAAGAACCCCACTGCCTTAGACAAAGCTGTTGCCGAGATGCGCGAG	MNTLIFFTAVVAMASATGEEKILNDGNNVFTANMFSEVVK<br>EQAGKSVVMSAFSVLQPLAQLALASVGESHDELLRAIGLPN<br>DNVTKAVFPKVTAQLRAVQGVKLNMANKIYIPTNAQVKED<br>FAALSKSVFGSEFKNIDFTKNVPAAQEINSWVEDHTNHKIK<br>DLVDPNSLGADTRAVLVNALYFKGSWEKKFNDKATTDSDF<br>HVTKDKTVKVPTMYKKDDFKYAESKELDAKLLELPYEGKEA<br>SFLIVLPNEIDGLPALQEKLKNPTALDKAVAEMREVEVNVYL

		GTGGAAGTCAACGTCTACCTCCCTAAGTTCAAGATTGAAACTACTATCGACCTGAAGAAAGTTCTGCAAAAGATCGGCATTACCAGCCTTTTCGACGCCGCCAAGGCCAGATTGGACAAT<br>CTCTTGAAGAACGAAAGCGGTCTGTACGTCAGTGATGCCATCCAGAAGGCTTTCATTGAAGTCAACGAGGAGGGTGCCGAGGCAGCTGCAGCTAATGACCTCGGGATATATGTAATGAT<br>GCTAAAACCAGAAATATGGTTTAAGGCAGACCGGCCGTTTCTATTCTTCCTTAACGCCGGCTCCACTACTTTGTTCAACGGCTCCTACCAATCCTAA	PKFKIETTIDLKKVLQKIGITSLFDAAKARLDNLLKNESGLYVS<br>DAIQKAFIEVNEEGAEAAAANDLGIYVMMLKPEIWFKADR<br>PFLFFLNAGSTTLFNGSYQS
CpSPN1E	chr5	ATGAATACACTTATATTCTTTACAGCCGTCGTGGCCATGGCTTCAGCCACTGGTGAGGAGAAGATCCTCAATGATGGAAACAACGTGTTTACTGCCAACATGTTTTCGGAGGTGGTAAAA<br>GAGCAGGCCGGCAAGAGCGTGGTGATGTCCGCCTTCTCTGTCCTGCAGCCGCTGGCTCAACTGGCCCTGGCCTCTGTCGGCGAGTCCCATGACGAGCTCCTCAGAGCCATCGGCCTTCC<br>CAACGACAATGTGACCAAAGCAGTGTTCCCCAAAGTGACCGCTCAACTTCGAGCAGTACAAGGAGTGAAACTGAACATGGCCAACAAAATATACATTCCCACAAACGCTCAAGTTAAGG<br>AAGACTTTGCCGCACTCTCAAAGAGCGTCTTCGGTTCAGAATTCAAGAACATTGACTTTACGAAAAATGTGCCTGCTGCTCAGGAAATCAACTCATGGGTGGAGGATCACACAAACCAC<br>AAAATCAAGGACTTGGTCGACCCGAACTCCCTGGGAGCTGATACTCGTGCGGTCCTCGTCAATGCTCTGTACTTCAAGGGTTCGTGGGAAAAAAAATTCAATGATAAGGCTACTACTGAT<br>AGTGATTTCCATGTAACTAAAGACAAGACAGTGAAAGTGCCGACGATGTACAAGAAAGACGACTTTAAATACGCCGAGAGCAAAGAGCTCGATGCTAAGCTCTTGGAACTGCCATATGA<br>AGGAAAGGAGGCATCTTTCCTTATCGTCCTCCCCAACGAAATCGACGGTCTGCCAGCTTTGCAGGAGAAACTGAAGAACCCCACTGCCTTAGACAAAGCTGTTGCCGAGATGCGCGAG<br>GTGGAAGTCAACGTCTACCTCCCTAAGTTCAAGATTGAAACTACTATCGACCTGAAGAAAGTTCTGCAAAAGATCGGCATTACCAGCCTTTTCGACGCCGCCAAGGCCAGATTGGACAAT<br>CTCTTGAAGAACGAAAGCGGTCTGTACGTCAGTGATGCCATCCAGAAGGCTTTCATTGAAGTCAACGAGGAGGGTGCCGAGGCAGCTGCAGCTAATGCATTTCTACTCGTGGAGGAGT<br>CGTTGGCCCTAGTCGAAGAGCTGTACTTTGACGCCGACCATCCATTTATTTTTCATCTTAGGCAAGGAGCCCGTACACTTTTTAACGGAGTTGTCAATTTCTGA	MNTLIFFTAVVAMASATGEEKILNDGNNVFTANMFSEVVK<br>EQAGKSVVMSAFSVLQPLAQLALASVGESHDELLRAIGLPN<br>DNVTKAVFPKVTAQLRAVQGVKLNMANKIYIPTNAQVKED<br>FAALSKSVFGSEFKNIDFTKNVPAAQEINSWVEDHTNHKIK<br>DLVDPNSLGADTRAVLVNALYFKGSWEKKFNDKATTDSDF<br>HVTKDKTVKVPTMYKKDDFKYAESKELDAKLLELPYEGKEA<br>SFLIVLPNEIDGLPALQEKLKNPTALDKAVAEMREVEVNVYL<br>PKFKIETTIDLKKVLQKIGITSLFDAAKARLDNLLKNESGLYVS<br>DAIQKAFIEVNEEGAEAAAANAFLLVEESLALVEELYFDADH<br>PFIFHLRQGARTLFNGVVNF
CpSPN1F	chr5	ATGAATACACTTATATTCTTTACAGCCGTCGTGGCCATGGCTTCAGCCACTGGTGAGGAGAAGATCCTCAATGATGGAAACAACGTGTTTACTGCCAACATGTTTTCGGAGGTGGTAAAA<br>GAGCAGGCCGGCAAGAGCGTGGTGATGTCCGCCTTCTCTGTCCTGCAGCCGCTGGCTCAACTGGCCCTGGCCTCTGTCGGCGAGTCCCATGACGAGCTCCTCAGAGCCATCGGCCTTCC<br>CAACGACAATGTGACCAAAGCAGTGTTCCCCAAAGTGACCGCTCAACTTCGAGCAGTACAAGGAGTGAAACTGAACATGGCCAACAAAATATACATTCCCACAAACGCTCAAGTTAAGG<br>AAGACTTTGCCGCACTCTCAAAGAGCGTCTTCGGTTCAGAATTCAAGAACATTGACTTTACGAAAAATGTGCCTGCTGCTCAGGAAATCAACTCATGGGTGGAGGATCACACAAACCAC<br>AAAATCAAGGACTTGGTCGACCCGAACTCCCTGGGAGCTGATACTCGTGCGGTCCTCGTCAATGCTCTGTACTTCAAGGGTTCGTGGGAAAAAAAATTCAATGATAAGGCTACTACTGAT<br>AGTGATTTCCATGTAACTAAAGACAAGACAGTGAAAGTGCCGACGATGTACAAGAAAGACGACTTTAAATACGCCGAGAGCAAAGAGCTCGATGCTAAGCTCTTGGAACTGCCATATGA<br>AGGAAAGGAGGCATCTTTCCTTATCGTCCTCCCCAACGAAATCGACGGTCTGCCAGCTTTGCAGGAGAAACTGAAGAACCCCACTGCCTTAGACAAAGCTGTTGCCGAGATGCGCGAG<br>GTGGAAGTCAACGTCTACCTCCCTAAGTTCAAGATTGAAACTACTATCGACCTGAAGAAAGTTCTGCAAAAGATCGGCATTACCAGCCTTTTCGACGCCGCCAAGGCCAGATTGGACAAT<br>CTCTTGAAGAACGAAAGCGGTCTGTACGTCAGTGATGCCATCCAGAAGGCTTTCATTGAAGTCAACGAGGAGGGTGCCGAGGCAGCTGCAGCTAATGTGTTCCCCGTTCCCATGGCAG<br>CAAGTTGGCCTCCACCTAGAGTGCGGGAGTTTATTGCAGACCATGCCTTCATTTTCCTCCTGAAGGCGGGAGATAGCGCACTCTTTGGCGGAGTATTCGCTAACTAG	MNTLIFFTAVVAMASATGEEKILNDGNNVFTANMFSEVVK<br>EQAGKSVVMSAFSVLQPLAQLALASVGESHDELLRAIGLPN<br>DNVTKAVFPKVTAQLRAVQGVKLNMANKIYIPTNAQVKED<br>FAALSKSVFGSEFKNIDFTKNVPAAQEINSWVEDHTNHKIK<br>DLVDPNSLGADTRAVLVNALYFKGSWEKKFNDKATTDSDF<br>HVTKDKTVKVPTMYKKDDFKYAESKELDAKLLELPYEGKEA<br>SFLIVLPNEIDGLPALQEKLKNPTALDKAVAEMREVEVNVYL<br>PKFKIETTIDLKKVLQKIGITSLFDAAKARLDNLLKNESGLYVS<br>DAIQKAFIEVNEEGAEAAAANVFPVPMAASWPPPRVREFI<br>ADHAFIFLLKAGDSALFGGVFAN
CpSPN2	chr1	ATGTCAACGTTTTTACTAACCTTTCTGTTTTTTATATTTTCAACAAACCAAATCAGTTCCACAAATATGGATTCCGCTGCTGTATCCTCGGCTGTCGCCAAGTTCTCCATGCAGTTCTGCAATG<br>AGCTGGATAAAAGCCAGAATGTGGTGTCTTCCCCCCTATCGGCTGAAATTCTGCTGGCACTCCTTTCACTTGGATCCTCTGAACCTGCCCTTCCAGAGCTTCTAGGAGCCATTGGATTTCA<br>AGACACTGATGCAATCCGCTCATCATTTTCACTTGTGTCATCAAGACTGAAATCCGTGAAAGGAGTCACTTTGGATGTGGCCAACCGGGTTTACTTACAACAGGGCCATGAGCTGGATCC<br>CAAGCTTAAAGATGATGCAGTTAAAGTCTTTGATGCAGGACTTGAGCCCATGGATTTTTCCAATGGTGCTGTTGCAGCAAAAGTTATTAATGACTGGGTGGAGAGCAAGACTAACAACA<br>AAATCAAGGATCTCATATCAGAAGACTCTCTCAATGCCCTGACTCGACTTGTACTTGTCAATGCTCTCTACTTCAAGGGTAAATGGAAGCATCAGTTTAAGCCCCATAACACAATGGACAAA<br>CCATTCTATGTGAAGAAGGACACTACTGTGGATGTTCCTATGATGTATATTGAAGAACAATTCAGATATGGGGAGAGTGACAAATTAAATGCACAGTTACTGGAGATGCCGTATGAAGGCG<br>ACGAGGCGAGCATGGTGATCGTGCTGCCCAAGGACATCGAGGGTCTCGACACGGTCCTCAAGCAACTGGCCTCGGGCGTCGACCTCGTCCAGGAGCTGAACAACATGATCTCCACCAA<br>GGTGCAGGTCACCGTTCCCAAGTTCAAGATAGAGACCGAGATCGACCTTAAGGAACTGCTGCCTAAGCTAGGCATCAAGGCCATCTTCGACGAAAAGAACTCTGGCCTAACCAAGATCT<br>TGGACAGCACCGAGCCCCTGTACGTGTCCAAGGCGATACAGAAAGCGTTCATCGAGGTGAACGAGGAGGGTGCGGAGGCGGCTGCGGCCACCGCGATGAATATTATGATGTGTTGTGC<br>AATGGTGGACGTCGCCCCCGTGCCAGTATTCACTGCGGATCGACCTTTCCTCGCGGTCATCCAAGCTCACGGAATACTTTACTTCCTGGCCGTACACAGCGGGAACGAATAA	MSTFLLTFLFFIFSTNQISSTNMDSAAVSSAVAKFSMQFCNE<br>LDKSQNVVSSPLSAEILLALLSLGSSEPALPELLGAIGFQDTD<br>AIRSSFSLVSSRLKSVKGVTLDVANRVYLQQGHELDPKLKDD<br>AVKVFDAGLEPMDFSNGAVAAKVINDWVESKTNNKIKDLI<br>SEDSLNALTRLVLVNALYFKGKWKHQFKPHNTMDKPFYVK<br>KDTTVDVPMMYIEEQFRYGESDKLNAQLLEMPYEGDEAS<br>MVIVLPKDIEGLDTVLKQLASGVDLVQELNNMISTKVQVTV<br>PKFKIETEIDLKELLPKLGIKAIFDEKNSGLTKILDSTEPLYVSKA<br>IQKAFIEVNEEGAEAAAATAMNIMMCCAMVDVAPVPVFT<br>ADRPFLAVIQAHGILYFLAVHSGNE
CpSPN3	chr3	ATGACTTTAATGCTGTGTTTAATACTTTTTGTCGCTCCACTGTCACAATGCGACGTGGTTCCCGACCAACAAATCCTCCAAAGTGTATTCGGAACCCCAGCGGGCTACGCTGGCCCTCTCCC<br>CGCAGCCCAACCGGGCAAGCCAACACTCGGAACAAATGAGACCCTGGTGGACCATGATTATTGGGACTCCGACGATTATTATGTGCAGTCGCCGGCCGACTACGACTCGTTTGATTGGA<br>CGGTTACTAAGCGCATAGCGGCATACTCAGAGGAAAACTTCCTACTTTCGCCTTTAGGCTTGAAACTAGCGCTGGCCATCTTAACTGAAGCAGCGACTGGCCCCACTCAAGCCCAGTTGG<br>CCTCGGTCCTAGGCTTTGACGTCAATAGAACCGTTGTTCGGACTAAGTTCTCGTCCATCATATCTTCTCTCCAGCTAGCAAATCCAAACTACACCCTGAACGTTGGCAGTAGAATCTACGTA<br>GATAGCACAGTGTCTGAGAGACAGCGGTTCTCTTCGATTGCTGAAGAATTCTACAAAACTGAGCTGAAGAAGGTGGACTTCCGCCTCCCCGCGGAAACTGCGGCCCAAATCAACGGAT<br>GGGTCTCCAATTTGACTCACGGGAATATACCTAATCTAATCGATGAAGACGAGGTGAAAGGCAGTATAGTCCTGGTCCTAACGACCCTGTTCTTCAAGGGCACCTGGAAGCACCAGTTCA<br>ACCCGAACACTACCAAAACCAGGGCCTTCTACGTGACACCGACTGTACAAAAACAAGTGCCCTTCATGAATGTAAAGGACAAGTTTTATTATGCAGAGTCAGCGAAATATGACGCAAAGA<br>TTTTGAGGATGCCATATTTGGGTAAAAAGTTCTCCATGTACATAGTAGTGCCGAACTCCTTGAAGGGCCTTCCTCGAGTAATGGACTCGCTGAGCGACCTGCGCACGGAAATGCTATACCT<br>CGCCGAGCGCACGGTTGATGTCACGCTCCCGAAATTCAAGTTCGACTTCACCTCCATATTGGATGGAGTCTTGAAAGATTTGGGCATTCGGCAAGCGTTTGAAGACACGGCATCCTTCCC	MTLMLCLILFVAPLSQCDVVPDQQILQSVFGTPAGYAGPLP<br>AAQPGKPTLGTNETLVDHDYWDSDDYYVQSPADYDSFDW<br>TVTKRIAAYSEENFLLSPLGLKLALAILTEAATGPTQAQLASVL<br>GFDVNRTVVRTKFSSIISSLQLANPNYTLNVGSRIYVDSTVSE<br>RQRFSSIAEEFYKTELKKVDFRLPAETAAQINGWVSNLTHG<br>NIPNLIDEDEVKGSIVLVLTTLFFKGTWKHQFNPNTTKTRAF<br>YVTPTVQKQVPFMNVKDKFYYAESAKYDAKILRMPYLGKKF<br>SMYIVVPNSLKGLPRVMDSLSDLRTEMLYLAERTVDVTLPK<br>FKFDFTSILDGVLKDLGIRQAFEDTASFPGLARGQSPQNRLK

		CGGCCTGGCTAGAGGGCAGTCTCCACAGAACCGACTGAAGATTTCCAAGGTTCTACAGCGCTCCGGCATCGAAGTCAACGAGCTGGGAAGCGTTGCGTACTCTGTCACAGAGGTGTCG<br>CTAGTAAATAAGTTTGGCGAGGACGAATACAGCGCCGGAGAGGTGATCGCCAACCGACCATTCTTGTTCTTCATAGAAGATGAGGCCACGAGACAACTGCTGTTCACCGGGCGGGTGT<br>CGGACCCTCTTGTGGCCACCGCGGAGTTCAAAGCGCACACACCCAGTGCGTAA	ISKVLQRSGIEVNELGSVAYSVTEVSLVNKFGEDEYSAGEVIA<br>NRPFLFFIEDEATRQLLFTGRVSDPLVATAEFKAHTPSA
CpSPN4	chr2	ATGCTTATATTTGTGCTCGAATTATGCGCGTGTTTTGTGTTGTGTTTCGGGCAGAATCAAGGAATTAAAGGACACGATGAACCGGAATATCCCACGAACTTACACAACGGCCTCAGCGAG<br>GCGACGGGGAATTTCTCAGTGGAGCTATTATATCATGCAGCAAAAAAACAAGGGAAGCAGAATCTCATATTGTCGCCGATCACAGCGTGGACGGTGTTGGCGGTGACGTCGGAAGGCG<br>CACTGGGCAATACGCGGACTCAACTGGTCTCGGCCTTACGAATAAAAAGAAACCGGACGGTCACCAGACATGACTTCCAAGAAATCGCGCGATGGCTAGTCGTAAACACAACTACGGTG<br>CATCTTGCTAAATTCAATGGAATATTTGTGGACAAGTCTAAATCTCTGGAAGATGACTTCCGTCAATCTTCCAAAACATTTTATGATACGCAAACTGTCGATTTAGACTTCAAAAACAGCGC<br>AGCAACCGCCGCCACCATCAACGGAGCCGTGTCAACAGCAACCAAAGGCAGAATTCCTAAATTGGTTGATGAAAGTAATTTGAGTGATGCACAGATGATCATCACGAGTGCAGTATATTT<br>TAAAGGCCAGTGGACTGCACCTTTTAATGTCTCCTCTACAACTAAACAAAATTTTTATAATAGTAGTGGGATGAAAATTGGGGAAGTGAACATGATGTACAATAGATACACATACCCATTTG<br>CTAATATTAAGGAGATTCAAGCAAGAGTAATTGAGTTGCCTTATGGTGTTGAAAATCGTCTGTCCATGCTATTAATGCTGCCAAATCCTGGTGTTACTCTAGAAGATATGTTCTTAAACTTCA<br>AAAAAGTACCTCTTGATACTGTGTTTCAAGAGCTGAAACTCTCTCAAGAAGAGTATAGTGATGATGAAATAGATTGTTTCATTCCTCGCTTCAAAATTGATTCAAGCATTGAACTGAATGAA<br>ATATTGAAAGAGGGAATGGGAATATATGATCTGTTTGACCCTGTGAAAGCCAATTTGGGCCGCTTGGCTCGGGTGCCCATGTATGTGACTAGGGTTATCCATAAAGCAGAAATTGAGGTT<br>ACAGAGGAAGGTACAACAGCGTCTGGGGCTACAGCTGCAGAGTTTGCAAACCGGATTGGAGTTATAAGATTTGAAGCCAACCGGCCTTTTAGTTACCTTATTGTGGAAAAAGTCACAAA<br>CACAATCGTTTTCGGAGGAGTGTACCAACAGCCGATTTTGTACTGA	MLIFVLELCACFVLCFGQNQGIKGHDEPEYPTNLHNGLSEA<br>TGNFSVELLYHAAKKQGKQNLILSPITAWTVLAVTSEGALGN<br>TRTQLVSALRIKRNRTVTRHDFQEIARWLVVNTTTVHLAKF<br>NGIFVDKSKSLEDDFRQSSKTFYDTQTVDLDFKNSAATAATI<br>NGAVSTATKGRIPKLVDESNLSDAQMIITSAVYFKGQWTAP<br>FNVSSTTKQNFYNSSGMKIGEVNMMYNRYTYPFANIKEIQ<br>ARVIELPYGVENRLSMLLMLPNPGVTLEDMFLNFKKVPLDT<br>VFQELKLSQEEYSDDEIDCFIPRFKIDSSIELNEILKEGMGIYD<br>LFDPVKANLGRLARVPMYVTRVIHKAEIEVTEEGTTASGATA<br>AEFANRIGVIRFEANRPFSYLIVEKVTNTIVFGGVYQQPILY
CpSPN5	chr23	ATGAGATTCACAGTTTTAATATTGTTAACATCATCATGTTACTGTGATGTGGAATTCAGTGAACGACCAAGAAACTTTAGCATAGAACTGCTTTATCATACTCAGAAGCAAACTGGTGGACA<br>TGTTGTGATATCTCCTTTTGGAATTTGGACACTTATGACCGGTTTAGCCGTGGGTGCGACCGGCGACAGCTTCGAGCAAATAAGAGTGGCTTTTATACTTCCCAGGAGCCTTAAAACCGTC<br>GTATGTGGATCTAAACACTTGTCTAAATCAGTGCTGAAACGGACCTCCGATGGAGTATCTATAACGAGTAAGAATTTTCTATTCGCTGATGATGATTTCTTACTTAAACCAGCATTTAAGCAG<br>ACTATTACTGAAGATTTCGACGCCACTGTCAAAGTGCTTGATTTTAAAGATTCTAACGAAGCTGCAGCTAAAGCAAACAATTTTATACAAAACTCCGGTGCTCGAGTTACTAATGTACTAAC<br>TTCAGAAGATTTTGAACAATCAAGAATGATTTTGACAAACGTGATAACTTTTAAAGGACTATGGACATCGCCTTTCAATGCAAGTCATACAACTCTTGAATATTTCTATAACGAAAACAAAG<br>AGATAATAGGCGAAGTGAATATGATGCATCAAAAAGGAGAATTTCCATTATCTAACATAATAGATCTTAAATCATTCGTCTGTGAACTGACTTATGGAACTGATGCGGCATATTCCATGCTAG<br>TGATCTTGCCTCATCCAGGTGTTAAAGTTGAAGACGTTTATAAGAAGTTTGAAAATATCACCATACCTGACATAATAGCGAGACTCCAAAACGATGAAAAGGAATACGGTTTAGATGAAGT<br>TGAAATTAAATTACCTAGATTCAAAATAAGCACGAATATAGTTATGAATAGACCTTTAAACGATATGGGTGTTTATGATATTTTCGATCCAGAATATGCTAGTTTTAGCAGAGCAACAAATGA<br>GAGTATTTATGTGTCGGCCATAGTTCATAAAGCAGACATAGAAGTAAGTGAAACAGGGACCGTTGCGTCAGCGGTATCTACAGCGTATTTAAGTAATAGATTTGGAACAACTAAATTGATT<br>GCTGATAGACCTTTCCTGTACTTCATCATTGAGAAGTCGACAGCTACTGTGATTTTTGCGGGCATTTATTCTAAGCCTTCTTTGTACTGA	MRFTVLILLTSSCYCDVEFSERPRNFSIELLYHTQKQTGGHVV<br>ISPFGIWTLMTGLAVGATGDSFEQIRVAFILPRSLKTVVCGS<br>KHLSKSVLKRTSDGVSITSKNFLFADDDFLLKPAFKQTITEDF<br>DATVKVLDFKDSNEAAAKANNFIQNSGARVTNVLTSEDFE<br>QSRMILTNVITFKGLWTSPFNASHTTLEYFYNENKEIIGEVN<br>MMHQKGEFPLSNIIDLKSFVCELTYGTDAAYSMLVILPHPG<br>VKVEDVYKKFENITIPDIIARLQNDEKEYGLDEVEIKLPRFKIS<br>TNIVMNRPLNDMGVYDIFDPEYASFSRATNESIYVSAIVHK<br>ADIEVSETGTVASAVSTAYLSNRFGTTKLIADRPFLYFIIEKSTA<br>TVIFAGIYSKPSLY
CpSPN6	chr1	ATGTTCAAAATAGTAGCCCTCGGGTTGCTTATAATTGCGACGGTGACTGGTCAATGCTTCTATAAAGATGATTCAGCTAAGAAACTGGACCCTGAGGCTCGCACGTCTCTTTATAGGGGCC<br>AATTGGAGTTCACCCTAAATTTGTTTAACTCCATTAATGACGCGTATCCCACCGACAACATATTCTTTTCGCCTTTCTCAGTATACCACTCTCTACTGCTAGCTTACTTTTCTGCTGGGGGCCA<br>GACGGAAGCGTCTCTGAAGGAGTCACTACAGATAGAAAAATCTTTGGATAAAATCAATTTGATATCAGCATACAAGGTAGACCGCCGGTCCCGCTTGACGAATAACAACAGCGCCAGCTA<br>TGAGTTCAACAGCGCCAACAAAATGTTCATCGATGAAACGCTCGACGTCAGGCAGTGCTTGCTCGACATCTTCGAGGACGAACTAGAGAGACTTAATTTCCACGAAACGCCCTCGGAAG<br>CACGCGAGCACATTAACAAGTGGGTGGCCGCGGCGACGAAGAACCATATCCAGGCGTTGATCCCGGAGGACGGCATCACGCACACCACGAAGCTGGTGCTCGCCAACGCCGCCTACTT<br>CAAAGGCGTCTGGGCCTCTAAATTCCCCGCAGAAAGGACAAAGAAAGAGGCTTTCTTTGTTTCTGAAACTCTTCAAACGCTGGTACCGTTTATGAAGCAGAAAGGCACTTTCCATTTCAT<br>GGTGAGCGAAGACCTCGGTGCACAGATTCTGGAGCTGCCCTACAAAGGCAACGACATCAGCATGTACATCCTCCTGCCGCCCTACTCTATGAAGGAAGACATGACAAATCGTTGCAAGC<br>CATATGGAGAGTTTGCTTGGCCCTACTCTATGCAAAATTTATTAGTGTCAGATTTCCACAGGTGTGAAGAACATCATAGCCAACCTGACACCCCAGCGCGACTAGCCGCCGTAGTGGAGG<br>AAAGCTACATGGGCCGCGAGGTCATCGTCGAGATCCCCAAGTTCACCGTTGAGAAGAGCCTGCCCCTGAGGCCAATCTTGCAGTCTCTGGGAGTAGGCGACTTGTTCAACAGCACCTCC<br>GACTTCACGACGCTGACGGAGGACCAAGTCAGCTTCGACGATGCCGTCCACAAGGCCAAGATCCAAATCGATGAAGAAGGAACCGTGGCTGCAGCGGCAACGGCCATCTTCGGGTTC<br>CGGTCATCGCGGCCCGCGGAGCCCACCCGCTTCGTGGCCAACTTCCCCTTCGTGTACCTCGTGTACGAGCGGCCCACCAACTCTGTCCTCTTCATGGGCGTCTACAGGGACCCTAAGAAG<br>TAA	MFKIVALGLLIIATVTGQCFYKDDSAKKLDPEARTSLYRGQLE<br>FTLNLFNSINDAYPTDNIFFSPFSVYHSLLLAYFSAGGQTEAS<br>LKESLQIEKSLDKINLISAYKVDRRSRLTNNNSASYEFNSANK<br>MFIDETLDVRQCLLDIFEDELERLNFHETPSEAREHINKWVA<br>AATKNHIQALIPEDGITHTTKLVLANAAYFKGVWASKFPAER<br>TKKEAFFVSETLQTLVPFMKQKGTFHFMVSEDLGAQILELPY<br>KGNDISMYILLPPYSMKEDMTNRCKPYGEFAWPYSMQNLL<br>VSDFHRCEEHHSQPDTPARLAAVVEESYMGREVIVEIPKFT<br>VEKSLPLRPILQSLGVGDLFNSTSDFTTLTEDQVSFDDAVHK<br>AKIQIDEEGTVAAAATAIFGFRSSRPAEPTRFVANFPFVYLVY<br>ERPTNSVLFMGVYRDPKK
CpSPN7	chr2	ATGTGGAAGGTCATAATAGCCGCGTGCACGAGCCTGGTGGTCACCGCTCCGGTCGATGATCGGGTCGATTTCTCACCCATCAATCAATTCGCTCTAAGACTCTTAGACAACGCATATGCTT<br>TTCAAGAGAACTTTGGGCGACAAAATTTTGCTATTTCTCCATTATCAGTATGGAGCGTTTTCTCGCTACTCGCCGAAGGATCGGCAGGGGACACCTTCCAGGAACTAATGAAGGAACTTC<br>AGCTACCTCAGGATTTGAGGGCAACTCAAGAGCTACATCTTGCGGCGGATAGCGTTTTAAGAAGCAACGACCCTGATCTCGTGATTCACAAACAAGCAGCCTTGTTCCCCGAATGTTCAT<br>TAGAGATACACCAAGAGTTTTGTCAAGCAGCTAACATGTACAGGACAGAAGGACAAATAAGAAACGCAGTGAAAGAAGAATTTCTTAGAGATTTAAGGCTGCTGATAGTAGACGCTTTG<br>TACTTTAAAGCAAATTGGACACATCCTTTCGACCCAACCGCAACCAGAGAAGAAGATTTTTACAATGGCCAAGGGAAAACAATTGGACGAGTAAACATGATGTTCCACAAAGCATCTCAC<br>AATGTAGGAGACTCAAACTCAATAGGAGCACAGATTCTTGAAATGACATATGGGAAACATGAAGAATTCTCAATGTTGATCCTCTTACCTTTCGAAGGAATGCCACTGAAAACACTACTTA	MWKVIIAACTSLVVTAPVDDRVDFSPINQFALRLLDNAYAF<br>QENFGRQNFAISPLSVWSVFSLLAEGSAGDTFQELMKELQL<br>PQDLRATQELHLAADSVLRSNDPDLVIHKQAALFPECSLEIH<br>QEFCQAANMYRTEGQIRNAVKEEFLRDLRLLIVDALYFKAN<br>WTHPFDPTATREEDFYNGQGKTIGRVNMMFHKASHNVG<br>DSNSIGAQILEMTYGKHEEFSMLILLPFEGMPLKTLLSNLVT

		GCAATTTAGTTACGTCTCCATTAGACTGGATAACAGACTTCAAACGAGACGATAAACGGCCGGAAATTGATGTGTTTATTCCCCGTTTCAAAATATCTTCTCAGATTGATTTAATTCCTGCTT<br>TAAAATACACCGGAATTCATACAATTTTCGACAGTGAGAAGGCGCAGTTGCCTGGTATTTCTGACAGTCCTTTATTTGTATCTAAAACAATTCAAAATGTAGAAATAGATGTAAAAGAAGAA<br>GGTACAGTTGCCGCCGCAGCTACAGTGGTTGGGTTAGAGAATCGATTTCTTTCCCAGCGGTTTGAGGCTAACAAAGAGTTTGTTTTCATGATCACGCATAGGAAATCAAACGTTATCTTG<br>TTTGCCGGTGTGTACAGTGACCCTGCTGTTGTGTGA	SPLDWITDFKRDDKRPEIDVFIPRFKISSQIDLIPALKYTGIHTI<br>FDSEKAQLPGISDSPLFVSKTIQNVEIDVKEEGTVAAAATVV<br>GLENRFLSQRFEANKEFVFMITHRKSNVILFAGVYSDPAVV
CpSPN8	chr19	ATGATTCTTTTTGTGCTCAAATTGTGCGCGTGTTTTGTGCTGTGTTTAGGGCAGAATCATGGAGTTAAAGAACCGGTATATCGAATGAATTTAGCTAAATTCAGTGAGTCGACGGGCAATT<br>TTACAATAGAGTTGTTATATCACGTGGCAAAACGACAACCGGACCAAAACTTCGTTGTGTCCCCGTTCGCAGCGTGGACAATGCTAGCGGTGACTTATGAGGGCGCAGTGGGTAATACCA<br>ATGAAGAACTGATCTTGGCCTTACGATTTCCCCGAAAAAGCCCTCGAACAACAGTTAGATATTACTATCAAGAAATCGCGCAATGGCTTGTCGTCAACAGAACCACAGCCCAATTTACAAA<br>ATTCAGCGGAATATTCATGGACAGGGTATGGACTGGAATGGATAATGACTTCCGTCACATTGCGGAGGAGATTTATAATACAGAAGCAGTTGACTTACCGTTCAAAAACAAAGCAGCAGC<br>GGCCACCATCAACCGAGCAGTATCTCTAACTACCGAAGACAGGATTCCCACATTAATTGATGAAAGTCATTTGAATAACACACAGCTGATTATCGCCAGTGCTGTATATTTTAATGGGCAGT<br>GGACTGCACCATTTAATGTTATTACAGTTAAACGAGATTTCTATAACAGTATTGGGGAGAAAATTGGTGAAGTGAATATGATGTATAATAGATACACATACCCGTTTGCGAATATTAGTGAAC<br>TTCAAGCTAGTGTGATCGAGCTGCCTTATGGTGTTGAAAACCATATGTCTATGATTTTAATTCTGCCAAACCCTGGTATGACTCTAAAAGATATGTTCTTAATCTTCCTCAAAGCAAATCTTAA<br>GACAGTATTTCAAGAGCTGAAAGTCTCTCAAGAAGAGCGTGGTGATGTTGAAATAGATTGTTTCATCCCACGCTTCAAAATTGTTTCAAATATTGAATTAAACGAAATTTTAAAAAATGGT<br>ATGGGAATACATGACCTTTTTGACCCAAAGAATGTCTTCTTGCCTCGCATGTCCCGGTGGCCCATTCGTGGTACAAGGGTCATACATAAAACCGAGATTGACGTAACTGAAGAAAGCAGC<br>ACACCATCCGGAAATATAGCTCCAGAATTTGCAAATGACAGTAAAGTTCTAATTTTTGAAGCTAATCGGCCATTTAGTTATTTTATTGTGGAAAAACACACAGACACTGTTGTTTTGGCAGG<br>AGTTTACCGAAAGCCTATTTTATATTGA	MILFVLKLCACFVLCLGQNHGVKEPVYRMNLAKFSESTGNF<br>TIELLYHVAKRQPDQNFVVSPFAAWTMLAVTYEGAVGNTN<br>EELILALRFPRKSPRTTVRYYYQEIAQWLVVNRTTAQFTKFS<br>GIFMDRVWTGMDNDFRHIAEEIYNTEAVDLPFKNKAAAA<br>TINRAVSLTTEDRIPTLIDESHLNNTQLIIASAVYFNGQWTAP<br>FNVITVKRDFYNSIGEKIGEVNMMYNRYTYPFANISELQAS<br>VIELPYGVENHMSMILILPNPGMTLKDMFLIFLKANLKTVF<br>QELKVSQEERGDVEIDCFIPRFKIVSNIELNEILKNGMGIHDL<br>FDPKNVFLPRMSRWPIRGTRVIHKTEIDVTEESSTPSGNIAP<br>EFANDSKVLIFEANRPFSYFIVEKHTDTVVLAGVYRKPILY
CpSPN9	chr2	ATGGCCACTTATTTGTACTTGCTGGCCATCTTTCTGCCTCCGATATTAGGGCAGTGTTCGGTCGAAAAGGCCGGTCATAGTTTCAGTCGCGCGGTTTACGAGTTTTCTCTGGATTTAATACA<br>AAGAGTGGGACAGGAAAACGATAACCATTTCGTCACGTCACCATTCTCGCTATGGACTGTTCTAACAACAGCATCTTTAGGGGCCGAAGACGAGACCCTTGCGGAAATGAAGCGAGTAC<br>TCCGCCTTCACCATTACCCGTGCTTCAACGACAGGTACCTAGAAATCGTGAAAGGGATCACAGTGTACAACTCGAAGACCACACTGGAGAGAAGTTCTGTGCTCTTCGTTGACAAGACT<br>GTTTCGTTGCTCGAATCTTTCCATAGAAAACTGAGTGGCACCGGAGTGTGCGACGTCATGATCCTACCTTTCGACAACTACAACAACGTGGCTACAACTATAAATAATTACGTGAGCCAAG<br>CGACCCACAACACCATCCAAGAAATCGTGAATTCAAATGACATCGAAGGAGTCTATCTGCTTCTGCTCGACGCTGTTTACTTTAAAGGAATGTGGAATATTCCCTTTGAGCCCTCAGAAAC<br>TGAAGTCACTTCTTTCTACGACGAGAGAAACAACCCTGTTGGAGATGTAAACATGATGTTCTCCATGCGGCAATATAATTTAGCATCCATTGATCAGCTACAAGCGAAGGTCCTAGAACTG<br>CCCTATGGAGATGGAGACCGTTTTTCCATGCTCATATTCCTTCCCTACCAATCCAGTTCTGTGGCCAAAGTCACAGACCAGCTGAAGAGAGTGACTTTAAAGTCCATATTCTACATTTTCCA<br>GCGTAACGGACGCGACACAGTTCAAGTTCAGATCCCGAGTGCTCAGTTCCCGCATATATCTGACTACAGTTTCTACATTTCGAATGTGGTGCAGAAGGCTGATGTGGAGGTGACAGAGG<br>AAGGGACGGTGGCGGCGGCGGCGTCGGCGGCGGAGTTCGAGGCAAGACGCCTACCTGACTCCTTCCAAGCGAACAGGCCGTTCGTGTTCATGATTGTGGACAAGTTGAACTATATTCC<br>GCTGTTCACTGAAGAAGATGGGACGGAGAGAAGCAGGCTGCGCGACAGCCTCACAGGATTTAGATAG	MATYLYLLAIFLPPILGQCSVEKAGHSFSRAVYEFSLDLIQRV<br>GQENDNHFVTSPFSLWTVLTTASLGAEDETLAEMKRVLRL<br>HHYPCFNDRYLEIVKGITVYNSKTTLERSSVLFVDKTVSLLES<br>FHRKLSGTGVCDVMILPFDNYNNVATTINNYVSQATHNTI<br>QEIVNSNDIEGVYLLLLDAVYFKGMWNIPFEPSETEVTSFYD<br>ERNNPVGDVNMMFSMRQYNLASIDQLQAKVLELPYGDG<br>DRFSMLIFLPYQSSSVAKVTDQLKRVTLKSIFYIFQRNGRDT<br>VQVQIPSAQFPHISDYSFYISNVVQKADVEVTEEGTVAAAA<br>SAAEFEARRLPDSFQANRPFVFMIVDKLNYIPLFTEEDGTER<br>SRLRDSLTGFR
CpSPN10	chr3	ATGCGAATACTATTTGTGCTATGCCTCGCGGTGGGTCTGAGCAGCGCGCGGTGGGTGCGGCGCGGCCGAGCCCCGCCCAAGACCACAGGCTTCGTCGGGGAAGCCACCAACCAACTAT<br>CCACTGCCATCTTTCAGGGTTACATCGACGATAATAAGAATATTGCATTCTCACCACTTGGTTACTCTGCTATCCTCGCCATACTTGCCGAAGGCGCGACGGGTCAGACGAGGGAACAACT<br>CGTATCTGCCCTCCACCTCCCAGAGAATCCGAACCTTATTAGAAAAACATATCGATACATCATGGAAAGACTGAAGAATACACACGAGTACAAATACAACCAACCTGAATTAAAGAATTATT<br>TCTACATCTATAAAAATTACACAATCAACGATGACTACAAGAAAATCTTAGAAGATTATTACCTTACAGATGTTAGATCCGTAGAAAGGTATAATGACGTAGACCACTTTAAGCCCGATGAAT<br>CTAATGATGATGATGAAACAATTGAAATTTCCGACAAGAAAGCTGCAGAAAGTAACAAGAATGAAGAAATTGTAGACCTTGTTCCACCTAAAGAATCTGGTGAGAAGTTTATTTCCTTTG<br>CTGTAGAAGATAAACCTGAAAAAGTAGACATATCTCAAATTGAGTACAAACCCGCGAAGAATATTAAAGAAAAGATAAAGCTTGTAAAAACTTACCCCAAAAAGGACGATTCCGGAGAA<br>GAAGAAGAGACTATGGTTGCTGTCGAGGCTAGAAATCATGCGAGAAGCCACCACCACGTTTTGCAGGAAAAATCCGATATTGCGTCAAGTATATCGGTTAATACTGTCGGAAAGAAATC<br>GGAAAAAACTTCGCCATCAAACTCTCTGATGCTTATCTTCAACGGGATGTATTTCCGCGGCGCGTGGAAACAACCTTTCCTAAAGGTCGAGCCAGCTATGTTCTACACGTCCAACAGTGA<br>GAAAAAGCAAGTGCAGATGATGTGTACGAAGGGCATCTTCAAGACTGGCTCTCTTCCTGGTCTTGACAGTTCAGCTATTGAACTGCCTTACGATGGCGGTCGCTACTCCTTGCTGATCGT<br>GTTGCCTCGCACCCGCGACGGTCTGACGCGGCTGACGGCTGATCTGCCCGCGGCGCCGCTCGAAGACATCCAGGACAGCTTGCGCGAGGAGGAGCTGCAGGTTTCGCTGCCCACATT<br>CTACGTGGAGACCACTACAAAACCAGTTGCAGCCTTAGCCAAGTTCGGAGTGAGCAGCATCTTCAGCCGCGACGCCGAGCTGTCGGGTGTTTCTTCAGCGGAGGGACTCTTCGTGCAG<br>GAGCTGGTCCAGCACGTAGCGGTTCGCGTCGACAACGCGGACACCTCTGCGTCACAACTGTCAGCAATTAACGCAGTCCAGGAGTCTCTTAAGAACATCCCATTGTCCAGCATCAAAGA<br>ACCACGCCGCTTCAGCGTCGACCGACCCTTCATGTTCTTCATCATTGATCGCCTTGACAACTTGGTGGTTATTGCTGGAAAAGTTATAGACCCAGAAGCACCGACGCCGTTCGAAATTTAA	MRILFVLCLAVGLSSARWVRRGRAPPKTTGFVGEATNQLST<br>AIFQGYIDDNKNIAFSPLGYSAILAILAEGATGQTREQLVSAL<br>HLPENPNLIRKTYRYIMERLKNTHEYKYNQPELKNYFYIYKN<br>YTINDDYKKILEDYYLTDVRSVERYNDVDHFKPDESNDDDE<br>TIEISDKKAAESNKNEEIVDLVPPKESGEKFISFAVEDKPEKV<br>DISQIEYKPAKNIKEKIKLVKTYPKKDDSGEEEETMVAVEAR<br>NHARSHHHVLQEKSDIASSISVNTVGKKSEKTSPSNSLMLIF<br>NGMYFRGAWKQPFLKVEPAMFYTSNSEKKQVQMMCTKG<br>IFKTGSLPGLDSSAIELPYDGGRYSLLIVLPRTRDGLTRLTADL<br>PAAPLEDIQDSLREEELQVSLPTFYVETTTKPVAALAKFGVSS<br>IFSRDAELSGVSSAEGLFVQELVQHVAVRVDNADTSASQLS<br>AINAVQESLKNIPLSSIKEPRRFSVDRPFMFFIIDRLDNLVVIA<br>GKVIDPEAPTPFEI
		ATGACTACTGTCGAACCAAAACAAAATTTGCCTTCTTTCACCATGCAAGATTTTGCTAAGATCTTGGACTACATAGGTGTTTTCTTAGCCTACCTTCAAAGTTTAGAGTGCATTGATAATAAG<br>AGGCCAGAAAATGCTCGTCTAAACTATTTCGACACAGATCTCCTTAAATATACGACTGAAGACCGAAGTGGCAATGTGGTGGTCTCCCCGGCGAGCATCAAATCGACTCTGGCTATGCTCC<br>TTGAAGGAGCAAGTGGCAACACTGAAGTTGAAATAAGATGTTCCTTACGATTGTCACCGAATAAAAATGAGTTCAGAGAACAACTGAATATCTATCTATCAGCGTTACAATGCAATAATAC<br>TGCAGCCAAACTTCAAAATGCTAATGGCGTCTTCGTGTCGAACAAATTGATACTGAAGAAAGAGTACGAGAGAGTCATAGAAAAAGTATACCTTACAAAAGTCGTCATTCTGAATTTTCTG	MTTVEPKQNLPSFTMQDFAKILDYIGVFLAYLQSLECIDNKR<br>PENARLNYFDTDLLKYTTEDRSGNVVVSPASIKSTLAMLLEG<br>ASGNTEVEIRCSLRLSPNKNEFREQLNIYLSALQCNNTAAKL<br>QNANGVFVSNKLILKKEYERVIEKVYLTKVVILNFLDPVGSA

CpSPN11	chr3	GATCCGGTAGGGTCTGCTGCCATCGTAAACGATTGGGTTGGCAACCAGACCAGGGGGCTAATACGATCCCTAATAGAACCTGCCCACATCCCCCCATCATCCGACGTTCTCGTCATCAACG<br>CTCTGTACTTCAAAAGCGCCTGGCAGCACGCCTTCGATCGCAGCCTAACAAGGTCTGCCTGTTTTCGCGTCCAGGGCGCTTGTAAGAAGCAAGTCGCTATGATGGAGATCAGTACTGAG<br>CTCAACTACGCGTTCGTTGACAACTTGAGGGCTCATGCCGTTGAATTACCTTATGAGGGTGGCCGTTATTCTATGGTGTTATTAGTACCGCAAGACCACGACGGCTTGGCAGGACTGATAA<br>GGGACTTGCCGTACATGAGCCTGCCGCAGATATGCAAGATGATGGAACCGTCTCACGTGCAGCTCTCGTTGCCGAGGTTTACCGTGGACTATAGCGAGGATATGGTCGGACCACTTAGG<br>AGTATGCGAATCACAACCCTCTTCTCGTCAAAATCCAACCTGTCTGGAATCATCGAAGGTGGCCCCGCCCAAATCAACCACTTATACCACAGGGTGTACGTATCCGTTGACGAAGACGGAA<br>CAGTGGCCGCTGCAGCTTCTGCTGCCCTTGTCGTGCCTTTAATAGAAGGGAATGTGCAACTTGTGGTGGACAAACCCTTTGCGTTCTTTATAAAGGACAATACTTTGGGTTTGGTTCTTTT<br>TGAAGGAAAGATTGAAGAGCCACATGAATATGTGGCACCGAAAAAGGGTTCAGAGAACAATTTTGTTAAAAGCAGTCGGAATAGTTTTGGGACTCGCTTCTGA	AIVNDWVGNQTRGLIRSLIEPAHIPPSSDVLVINALYFKSAW<br>QHAFDRSLTRSACFRVQGACKKQVAMMEISTELNYAFVDN<br>LRAHAVELPYEGGRYSMVLLVPQDHDGLAGLIRDLPYMSL<br>PQICKMMEPSHVQLSLPRFTVDYSEDMVGPLRSMRITTLF<br>SSKSNLSGIIEGGPAQINHLYHRVYVSVDEDGTVAAAASAAL<br>VVPLIEGNVQLVVDKPFAFFIKDNTLGLVLFEGKIEEPHEYVA<br>PKKGSENNFVKSSRNSFGTRF
CpSPN12	chr3	ATGAGGTTTCAGATTTTATTGACAATAGTCTGCCTGACTTTAAGAATAGATTCCCAAAGTCTGAAGCCGACACGGACTTCCAATAGATATGAACCTGGATCTGAACAGTTATTTTTCGAAGA<br>TAACTTGCCCAGGAATTATAATAGAAATAACGTGAATCTGAGTAGTGACAACGCAAAAGCCAGCTTACAACAGGCGCAGACGCAGTCCCTTAATGACAGTCACTTGCGAGCTGATCACCA<br>AGGACAACGAAACACAAGAAATGGTAACAAACAGGATGAAAGATTTAAGATCGTAAATAAAGCAGTAATTCAAAATGGTAACTTACAGAATCAGTACCAAACAATTAATCAAAACGATAA<br>TACACAGAGTCAAAACCAAAATCAATTTAATCCGAATTTGGACCAAAATTCAAACTACCAGTTTCAACAAAGCATAGAACTTGCACCGCCTTTGCAATCAAGTCAAGATTATTATTCCAAG<br>CAAAATTTAAAGCAATCAAATATCGGTGGTATTGACAACAGCTATACAACTCGAGCCAACGTCGTTCCTGTGTCTCAGAGTTCCCAGCTGTTGAATATGACCAATGATATAAAACTTGCGTC<br>TGCGGTTATCTCACATGTTGGTATTTTCATGATGAAGAGTGCAAGAGAAAATCATTCAAACGTTGTGGTTTCCCCAACGTCTGTGGTGAATATGCTGGCGCTCCTGCAAGCTGGCGCAAC<br>GGGCGTGACTCAAGAACAGGTCTCGAATGCATTGAGATTGTCTCCAGAGAAGTCAGCAGAAGCATTTCGACTTATGAATACCGAGTTGCGGAAGCGAAGTACCGAACGAAACATTTTG<br>CGATCGGCGAGCAGTATTTTCGCAGCAGATTCATTCGAACTTAGTCGTGATTTCAAGAATATTGCCATTCAAAACTTTGGCAGCGAAGTATCAAGAATCAGCTTTGATAACCCAAATGCTG<br>CTGCCCAGAAGATAAATTCTTGGATTGCTAGTAAGACAAATGACCAGATCGACAGGCTGGTATCACCAGATTCCATCGGTGTCAATACTCAACTCGTGCTTGCCAATGCGATATACTTCAAG<br>GGTATATGGGAGACCATGTTCAAACCGTCAGAAACGTCACTGCAGGACTTCAATCTAAGCAATGGTAGCAAAAAATCTGTTCCATTCATGACGCTGCGGAGTGGCTTCAGGGCAGGTGT<br>AGACAAGGCTAATAAAGCCCTTGTTATCGTTTTACCTTTTGAGAACAATGAATATTCCCTAGTCATGTTGCTGCCAGTTCCTGGCGCCGACGTATCAGCTGTGTTGACGTCGCTCGACGGT<br>GAAACTCTAGCAAACTACCAGAATTTACGGCGCCAGGATGTTCTTCTCGAAATACCGAAATTCACTGTCCGTAGTAATGCTAATCTGCAACCGATTTTCGCGAAGATGGGAATCACAAAGA<br>TGTTTTCTCCAACATCGGAACTCAGTCACATCGGCCTGTACCGTGCCAATTCGCCCCAAGTCTCCTCAGCGGTTCACTCCGCCATGATGTCAATAGATGAACGGGGTGCCACAGCCGCCGC<br>ATCTACAGTGGTTGCAGTTGTAGCATTGTCATTCGACAACCCGTCCGTTGTGTTCAGAGCAGACCGGCCGTTTTTAGCTGTGCTGTGGGATAATCAGCTAAACGTGCCTTTGTTCATGACC<br>AAGATTGAAGACCCTTCACTGTAA	MRFQILLTIVCLTLRIDSQSLKPTRTSNRYEPGSEQLFFEDNL<br>PRNYNRNNVNLSSDNAKASLQQAQTQSLNDSHLRADHQ<br>GQRNTRNGNKQDERFKIVNKAVIQNGNLQNQYQTINQN<br>DNTQSQNQNQFNPNLDQNSNYQFQQSIELAPPLQSSQDY<br>YSKQNLKQSNIGGIDNSYTTRANVVPVSQSSQLLNMTNDIK<br>LASAVISHVGIFMMKSARENHSNVVVSPTSVVNMLALLQA<br>GATGVTQEQVSNALRLSPEKSAEAFRLMNTELRKRSTERNI<br>LRSASSIFAADSFELSRDFKNIAIQNFGSEVSRISFDNPNAAA<br>QKINSWIASKTNDQIDRLVSPDSIGVNTQLVLANAIYFKGIW<br>ETMFKPSETSLQDFNLSNGSKKSVPFMTLRSGFRAGVDKA<br>NKALVIVLPFENNEYSLVMLLPVPGADVSAVLTSLDGETLAN<br>YQNLRRQDVLLEIPKFTVRSNANLQPIFAKMGITKMFSPTSE<br>LSHIGLYRANSPQVSSAVHSAMMSIDERGATAAASTVVAVV<br>ALSFDNPSVVFRADRPFLAVLWDNQLNVPLFMTKIEDPSL
CpSPN13	chr3	ATGGATTGGAGGTCGCGAGTCTTCCTGTTCCTGATGTTCATTAGCGTCGCCATCTCTGAACATGAGGAAAACACAACGGATCCGAGCGTCACAGACCAACCACATGAGCAACAGTTATCC<br>AACACAATCAACGAGTTGGGTTTCAAATTGCTCACTGAGGAGATGAAACGATACGCGGACACAAACATAGTTATGTCCCCTACTGGTATCGCAGGTCTCCTCGCGATGGTACTTCTGGGA<br>AGTGCTGGCAACACGTACACGGAGCTGGCAGCGGCGCTCGGGTTTTCCCAAGATATTCTCCGAAACCGCGCGCACCACGAACAGTTCGGCTCGCTGCTCCAACTCCTGAACTCCAGCA<br>CCACATACGCCGACGCCATATTGGTGGAGCCGCGTACACGTATTCGTCCGTTGTACAGAGAGTTCCTTCAACGCGTGTACCAAGGCGACGCGCGCGCTGTGGAGTTTACCGACGCGCCC<br>TTGGCTAGAGACACCGTCAATGATTGGATCAAAAACCACACGAAAGGGAAAATAGAAGAATTTCTGAAAGAACCCCTCCCAGTGGAGACAAGGGTTGTGTTGCTGAGCGCCCTCTACT<br>TCAGCGGACAATGGACTCATCCCTTCTTGCCGGAACACACGAAGAAGATGGCGTTCAAGACTCCTACTGGCGACGTCATGGCCGATATGATGTTGAACTTCGGACATTTCCAATACACAT<br>ACTCAGCTGAAGATGGCGTGCAAATGATAGCTTTGCCCTACAAAGACAACACAACCACGATGTACGTGTTGAAGCCACTTAAAAAACTTTCCTTGCAAGACCTCATGAGCCGCCTAAACT<br>ACTCGAGGATCGACCAACTCATCTCCAACATGACCAATGACAGAGCGGTCATCAGATTCCCAAAAATGGACCTAAAAAACTCAATCCAGTTAGAAGACAGCCTTAAATCTATAGGTCTCAG<br>CGAAATGTTCTCTCCCACTGCGAATTTCGCTCTCATGATCGATACGGATAGAGATAAATCTAATGAAGAATTGTTGACGAGAATAATTTTTGAGGATAAGTCGCAGCATATTCAGGATGCGG<br>TGAACGGCCTGCCCAATCCGGGGGTGCATGTGGACAAGGTGCTACATGAAGTTAGGATCACTATAGATGAATACGGCACGGAGGCGGTGGCGGCCTCGGGCGGGTTCCTGTCTCGCAC<br>GGCGGAGCAGTTCTATGTGGACTCCCCCTTCTACATGTTCATCAGGGACGAGAGCACCAAACTAGTCACCTTCAGCGCCGCCATTTTTAATCCTAATCTATAG	MDWRSRVFLFLMFISVAISEHEENTTDPSVTDQPHEQQLS<br>NTINELGFKLLTEEMKRYADTNIVMSPTGIAGLLAMVLLGSA<br>GNTYTELAAALGFSQDILRNRAHHEQFGSLLQLLNSSTTYA<br>DAILVEPRTRIRPLYREFLQRVYQGDARAVEFTDAPLARDTV<br>NDWIKNHTKGKIEEFLKEPLPVETRVVLLSALYFSGQWTHP<br>FLPEHTKKMAFKTPTGDVMADMMLNFGHFQYTYSAEDG<br>VQMIALPYKDNTTTMYVLKPLKKLSLQDLMSRLNYSRIDQL<br>ISNMTNDRAVIRFPKMDLKNSIQLEDSLKSIGLSEMFSPTAN<br>FALMIDTDRDKSNEELLTRIIFEDKSQHIQDAVNGLPNPGVH<br>VDKVLHEVRITIDEYGTEAVAASGGFLSRTAEQFYVDSPFYM<br>FIRDESTKLVTFSAAIFNPNL
CpSPN14	chr2	ATGAGACGCGCCGTTTTTTTGATAAGTTTTTGCTGTTACTCGTTAGCGAAGAACTGCGATGAGCGGTCCGTTACCGCGTACTACAAAAAGCCTATTTATGAGTTCTCAACCGGGCTTTTGG<br>ACAGAGTTTCCCAAGAAACGGATGGTCATTTCGTCTGTTCACCGATAAATCCGTGGCTGCAGCTCGTGAACCTCGCACAGGGCGCCCATGGAGCTACCCTCAAGCAGATCTGGAAGGTG<br>ACCGGACATGGGAAGCCATGTATGCAACGGACTTTGAGAAAAACCCTGAACAGCTTATACGGGGAAGGGAAGGACTATAAATGCCAAAGTTTAGTGATAATAGATAAATATTTAGCAGT<br>GAAAAATTATTTTAAAAGAAGAGTTGAACGATCAAAAACAGTTAAAGTGGCATCTTTAGATAGAGATACTCCTTATGACTCTGCAGATGAAGCTAGTGAAATTATAGAAAGAGCTACTGGT<br>GTTGGGAATAGTATAGACCCATTCGAGTTCAAGCTGACTTCTCTGATGTTGGCTGATGTGGCCACGTTTAAAGCTGCTTGGCGGCAACCCTTCGATCCTGGATTCACTGCTGTACAGCCTT<br>TCTACCACGGAAACGTTAAACTGGGCGAAGTCAATATGATGTCCAGCATAGGATACTTCAACTACATTAACTTAACAGCCATTAATGCCGAAGTTTTGGAACTTCCATGCATTGACAAAAG<br>GCGTTCTATGCTCATATTTCTACCCACAAACGGAACCATCAAAGATTTATTTTTCCACATGAAACGAATAAGGATAGCTACTATTTTCAATCTTTTCAAGAAATCCAAAAGCAAATTAGTGGT<br>TTTAAGATTGCCTAGGTTTAAGATAAGCAGTGAAATTGTGAACTTACCGGAGCTGATCAGCGATATGGGGGTGTCGTATATTTTTAAGATTAACGGTAATCTTAAAGGTATAAGTGATTTCC<br>CGATACGAGTGAATAGGATGAAGCAAGTGGTGGAGATAGAGGTGACAGAGGAAGGTGTGGCGGCTGATATAGTTCAAAATCTAATTGATCCGGAAGTGGAACCGATGAAGTTTGAGGC	MRRAVFLISFCCYSLAKNCDERSVTAYYKKPIYEFSTGLLDRV<br>SQETDGHFVCSPINPWLQLVNLAQGAHGATLKQIWKVTG<br>HGKPCMQRTLRKTLNSLYGEGKDYKCQSLVIIDKYLAVKNYF<br>KRRVERSKTVKVASLDRDTPYDSADEASEIIERATGVGNSID<br>PFEFKLTSLMLADVATFKAAWRQPFDPGFTAVQPFYHGNV<br>KLGEVNMMSSIGYFNYINLTAINAEVLELPCIDKRRSMLIFLP<br>TNGTIKDLFFHMKRIRIATIFNLFKKSKSKLVVLRLPRFKISSEI<br>VNLPELISDMGVSYIFKINGNLKGISDFPIRVNRMKQVVEIE<br>VTEEGVAADIVQNLIDPEVEPMKFEANRPFAFMLVDKPTEII

		AAACAGACCGTTTGCTTTTATGTTGGTAGATAAACCGACAGAAATCATATTATTTGCTGGAGTATACTCTATCCCATCAGTGTTCTAA	LFAGVYSIPSVF
CpSPN15	chr22	ATGGATCCGAAATTATCGCTAAGTCTTTGGAATTTTGCGGCGAGGTTTTGCAATGAGCTGGAGAAGGGTAAAAGTCAGGTGTGCTCCCCGTTGTCGGCGCAGGCGGTGCTGGCGCTGA<br>CGGCGCTCGGCAGCGGTGGCGACACCCGCAAGGAGCTGCTGCAGGCCATGGCGCTACCCGATGATGAGGCGATTCGCTCCTTATTTGGCTATTTAACATCCACTCTAAATTCAATCCAAA<br>CAGTCAAGCTGCGTTTCGCCAACAGAATATACGTGTCGAAGCATGGAACTCTGAAACCTGGCATCCAGAAGGACTCAGTAGAAGTCTTCGGATCTAGTCTGCAGCAGGTGGACTTCATG<br>GAAAAGGAGGGTGCCGCAGATAGTATTAATAAATGGGTAGCAGCAAAGACCAACAATATGATCAGAAACATAGTCTCTCCTAACTCGTTGCACTACGCCACCCGTTTGTTGCTGGTGAAC<br>GCCATCTACTTCCAGGGAACCTGGGAAAAGCCGTTTGAAAAGGATTTAACGAAAGAAAGGATATTCCATGTAAATAATAGGGACACAGTCGAAGTTCCTATGATGTTTATGGATGAGGAA<br>TATTTTCGGTACAGACACAGTCTAGAGTTGAAAGCACAACTCCTGCAGATGAATTACAAAGGCCGTCAAGCAAGCATGATACTAATAGTACCAGACGCAGTCGAAGGTCTGGGAGCCAT<br>GCTGCAGCAGCTCTCAGACCACGACCTGATGGATGAAGTGGCTGTGATGAGGGCCACCAGGATAGAAGTCATGATACCCAAGTTCAAGATAGAAACTGATATCAACCTGGAGGAACTGT<br>TACCAAAGATGGGAATCCATGATATATTCGATCAAAACAAGTCAGACATTCGAATGTTTACATCTCATGACAGGATGTGCGTGACGCAAGCCATCCAGAGGGCCGTCATCGAGAATGAGA<br>CGGGAACTGAGGCTGCAGCCTGCACACTAACCGCTATCAGCGAAATCTGCGCCGTCATAGAACTGGAACCTCCGAAGCGTTTTGTGGCAGACCGGCCGTTTCTGTATTTGCTGCTGGCG<br>CGTGGTGTACCCACCTTCATAGGCGTTTACCATCAGTAG	MDPKLSLSLWNFAARFCNELEKGKSQVCSPLSAQAVLALTA<br>LGSGGDTRKELLQAMALPDDEAIRSLFGYLTSTLNSIQTVKL<br>RFANRIYVSKHGTLKPGIQKDSVEVFGSSLQQVDFMEKEGA<br>ADSINKWVAAKTNNMIRNIVSPNSLHYATRLLLVNAIYFQG<br>TWEKPFEKDLTKERIFHVNNRDTVEVPMMFMDEEYFRYR<br>HSLELKAQLLQMNYKGRQASMILIVPDAVEGLGAMLQQLS<br>DHDLMDEVAVMRATRIEVMIPKFKIETDINLEELLPKMGIH<br>DIFDQNKSDIRMFTSHDRMCVTQAIQRAVIENETGTEAAA<br>CTLTAISEICAVIELEPPKRFVADRPFLYLLLARGVPTFIGVYH<br>Q
CpSPN16	chr2	ATGAAGACCATAGTTTTCCTACTATTTGTCGCGACATGTTATGCTGAGGAGTTTAGTGCGCGATCAAGAAACTTCAGCATCGAACTTTTGCATCATACACAGAAACAAACTGGTGGACATG<br>TTGTAATATCGCCTTTTGGAATATGGACCCTAATGACCGGAGTCGCGCTCGGTGCAACAGGCAACAGTTTCGCGGAGTTGCAACGCGCTTTCATCCTCCCAAGGAGCAAGAAAACCGTTA<br>TTGCCGGTTACAAAGAATTGACAAAAGCCGTGCTAAATCCATCCACAAACGGAGTATCACTAACAAGCAAAAACTTTGTATTCATTGACGACGATTTCACTTTGAATCCGGACTTTCGGAA<br>AACGATTAGCACAGATTTCGACGCAACAATAAAAGTACTCGATTTTAAGAATCCTGACCTGGCGGCCGGCAAAGCGAACAATTTTATCCAAAATTCCGGGGGGCGAGTCTCTAACGTGCT<br>AACCTCAGATGACTTTGCTGAGTCCAGGATGATTTTAACTAATGTGATATCCTTTAAAGGCTTATGGTTATCTCCCTTCAACACGAGCGAAACAACAGTTGAGAACTTTTACAATGAAAACA<br>ATGATGTCGTAGGGCAAGTCAACATGATGTATCAGAGAGCGGAGTTTCCATTCTCCAATATCGTTGAACTAAAAGCGTTTGTTGTCGAATTGCCGTACGGAAATGATGGAAAATATGCTAT<br>GCTGTTAATTCTGCCACATCCCCGAGTAAAGTTGGACGATGTCTATAGAAGGCTAGCGAATGTGACTCTTACAGAAATAACAAAGAAGCTACAGAGCGATATAGACGAATACGGAATGAC<br>TGATGTTGACATTAAACTTCCCAGATTCAAGATTAGTACGAATGTTGTTATGAACAAGCCCTTAAATGACATGGGTGTTTACGACATTTTTCAACCAGATGTCGCGAGTTTCAAACGTGTGA<br>CGAATGAGAATATTTATGTCTCGGCCATTGTGCACAAAGCTGACATTGAAGTCACTGAAACTGGGACAGTAGCCTCTGCTTCGACTTCCGCCAATTTCGCCGATAGGATATCGACGCCACT<br>TATGAGAGCCAATAAGCCTTTTGCGTACTTTATTATTGAGAAAACTACCACGACTGTTATATTTGGTGGCATATATTCGAAACCTACTGTTTATTAA	MKTIVFLLFVATCYAEEFSARSRNFSIELLHHTQKQTGGHVVI<br>SPFGIWTLMTGVALGATGNSFAELQRAFILPRSKKTVIAGYK<br>ELTKAVLNPSTNGVSLTSKNFVFIDDDFTLNPDFRKTISTDFD<br>ATIKVLDFKNPDLAAGKANNFIQNSGGRVSNVLTSDDFAES<br>RMILTNVISFKGLWLSPFNTSETTVENFYNENNDVVGQVN<br>MMYQRAEFPFSNIVELKAFVVELPYGNDGKYAMLLILPHPR<br>VKLDDVYRRLANVTLTEITKKLQSDIDEYGMTDVDIKLPRFKI<br>STNVVMNKPLNDMGVYDIFQPDVASFKRVTNENIYVSAIV<br>HKADIEVTETGTVASASTSANFADRISTPLMRANKPFAYFIIE<br>KTTTTVIFGGIYSKPTVY
CpSPN17	chr15	ATGACATCGATTTCTCACGTAGCGAATTTCGGGGCGCGGTTCTGCAATGAACTGGACAACAGCAAAAACTTCATCTGCTCTCCTCTGTCGGTGGAGACGGTGTTGGCATTAGTGGCCCTC<br>GGCAGCAGCAACGAATCTCGCGCAGAAATATTCAAGGCGCTCGACATCCCTAATGAAGACGATCTCTTTTCCACATTCATATCCCTGACAACAACTCTAGCTTCGAAGCCTTCAAAACATA<br>AAACATCCAACGACGTTACGTTAAAAATCGCGAACAAGATCTACGTGAAACAGGGGGCAGCCCTAAAGGCGGCTCTCAAGAAGAACGCCGAAGAAGTCTTCCAAGCCAGTTTGGAAC<br>AAATAGATTTTACACAAAGTAATAATGCTGCGAATGCCATTAATAAATGGGTGTCAAATCAAACCAACAATACTATTAAAGACATTGTTTCTGCCGACATGCTCGATGAAGACACACGACTA<br>GTACTCATCAATGCCATCTACTTCCACGCTTTCTGGGAGTATCAATTTGACGCCGACGATACATACACCAGAGTATTTCATGTCAATAAATCGAAGAAAATCGATATTAAACAGATGAGTTTA<br>ATGGGAAAACCTTTTAATTACAAACACAGTCCCGAGTTAAAGGCCAAAATACTGCAGATGTTCTACGTTGGAGGTGAAGCTAGTATGGTGATAGTTCTGCCAGATGAAATCGAGGGTCTA<br>AGTGCTGTGATGAAACAACTGGCAACCGGGTATGATCTGATGGCAGAGATCAAAAGTCTACAAAAAAAACAACTTCAAGTAATCATACCTAAGTTTAAAATTGAATCGGAGATCAATCTG<br>AAGGAACTGTTACCTAAGGGTACCTCCCCTACACGTAAAGTGGGGGTGAATATCTTTTTTTGCTGCAACCCTACAGTGTGGGGTATCGTTGGAAAGATGGGAATCAAGGCAATTTTTGAT<br>CCAATTAAGTCAGAGGTCAACATGTTGTCAAGTGATGATCGACAATTTGTATCCCAAGCCATTCAAAAGGCCTTCATCGCGGTGGATGAACTGGGCACGGAGGCGGCAGCAGCCAGTCT<br>TCTCACTACATGGCCGTACCAGCGCAAGCGCGATTCTACGACTTTGTCTTGTATCGGCCTGATCTTATATGTAGATTCCGTGGATGTAGACATTAGGCACTTTATCGAACATGGTCACTCCTG<br>CGGCCTACCTGAGCATCCGCATTTCCACCACATGAACTCTGTTCAGCTGTAA	MTSISHVANFGARFCNELDNSKNFICSPLSVETVLALVALGS<br>SNESRAEIFKALDIPNEDDLFSTFISLTTTLASKPSKHKTSNDV<br>TLKIANKIYVKQGAALKAALKKNAEEVFQASLEQIDFTQSN<br>NAANAINKWVSNQTNNTIKDIVSADMLDEDTRLVLINAIYF<br>HAFWEYQFDADDTYTRVFHVNKSKKIDIKQMSLMGKPFN<br>YKHSPELKAKILQMFYVGGEASMVIVLPDEIEGLSAVMKQL<br>ATGYDLMAEIKSLQKKQLQVIIPKFKIESEINLKELLPKGTSPT<br>RKVGVNIFFCCNPTVWGIVGKMGIKAIFDPIKSEVNMLSSD<br>DRQFVSQAIQKAFIAVDELGTEAAAASLLTTWPYQRKRDST<br>TLSCIGLILYVDSVDVDIRHFIEHGHSCGLPEHPHFHHMNSV<br>QL
CpSPN18	chr15	ATGGTGAAATGGGGACGGAGGAATAAGGTTGTGCAGTTCCTCGGCACACTCTCCGAAATGTATCCTGAATTGGACAGCAACAAAAACTTCATCTGCTCTCCTCTGTCCGTGGAGACAGT<br>GCTGGCACTGGTGGCCCTCGGCAGCAGGAACGACTCACGCGAGGAAATATTCAAGGCTCTAGACATCCCTAATGAAGATAATCTTCGTTCAGTGTTTACGTCTGTCACACAAAATCTGAA<br>GAAACCATTCATAGAATCTCTCACATCAAAACTCAATCCTTTTAATCGTGACTCCACCCTCCAAATCGCTACCAAAATCTACGTGAAATGTGGAAAATCTGTGAAACCGGAGCTTAAGAAAG<br>ATGCTGAAAAAGTTTTCAATTCCAGTTTGGAACAGATAGATTTTGTTAAAAGTTCTGATGCAGCAAATTCCATTAATAATTGGGTGAAAAAACGAACTAACAATATTATTAAGGACTTAGTT<br>TCTGCCGACATGCTGGACGGAGACACAGGACTTGTGCTAGTAAATGCAATCCACTTTCACATTTTGCAAATGCCCTATGTGGGAGGAGAAGCTAGTATGGTGATAATATTGCCTGATGAAA<br>TTGAGGGCGACACAGGTAGCGTGACTGATTTGGTGAAAAGACTGGCAGCTGGATTTGACCTGATGTCAGAGATCAAGCGTCTGAAAAAAACAGATCTTCAAGTAAAAATACCGAAGTT<br>TAAAGTTGAAACTGAGATAAATTTGAGGGAATTACTGCCGAAGATGGGAATCCAGACGATTTTCGATAAGAATAAGGCAGAAATTAACATGCTCTCTTCTAACGATCCACTACATGTATCA<br>GAAGCTATTCAGAAAGCTTTCATCACAGTGGATGAGAAAGGAACGGAAGCGGCAGCAGCTAATGAAATTACGTTGCAATTATTATGCTTGCGATGGTGGATGGGTGAAACGTTTATAGC<br>AGACCACCCCTTTCTCTACATACTTCTGGCTCAGGAGATACCTATATTTTACGGAATTTTTCATGGAAATGAAACTTCGCTGTAG	MVKWGRRNKVVQFLGTLSEMYPELDSNKNFICSPLSVETV<br>LALVALGSRNDSREEIFKALDIPNEDNLRSVFTSVTQNLKKPF<br>IESLTSKLNPFNRDSTLQIATKIYVKCGKSVKPELKKDAEKVF<br>NSSLEQIDFVKSSDAANSINNWVKKRTNNIIKDLVSADMLD<br>GDTGLVLVNAIHFHILQMPYVGGEASMVIILPDEIEGDTGSV<br>TDLVKRLAAGFDLMSEIKRLKKTDLQVKIPKFKVETEINLREL<br>LPKMGIQTIFDKNKAEINMLSSNDPLHVSEAIQKAFITVDEK<br>GTEAAAANEITLQLLCLRWWMGETFIADHPFLYILLAQEIPI<br>FYGIFHGNETSL
		ATGACATTGCATGTGAATCATGTGGCGAATTTCGCGGCGCGATTTTGCAATGAATTGGATAGCGAAAAAAACCTTTATCTGCTCTCCTCTGTCAGTGGAGACGGTGTTAGCATTAGTGGCCC	MTLHVNHVANFAARFCNELDSEKTFICSPLSVETVLALVALG

CpSPN19	chr15	TTGGCAGCAGTAACGAATCTCGCGCGGAAATATTCAAAGCTCTCGACATCCCTAATGAAGATAATCTTCGCTCTGTGTTTACGTCTCTCACCCAAAATTTAAAGAAGCCATTCATCGAAACC<br>ATTACATCAATACTTAAACCATTCAGCCAGGAGTATACACTTAAAATCGCCAACAAAATCTACGTGAAACAAGGAAAAGATCTAAAATCTAATTTTAAGAAGGATGCTGAAAAAGTCTTCA<br>ATGCTAGTTTGGAGCTAATCGATTTTGGCAAAAATGATGATGCAGCTAATTCTATTAATAACTGGGTAGCGAAACAAACAAATGATATTATTAAAGACATCGTTTCTGCTGACATGCTGATGC<br>AAGACACACGGGTTGTGTTAGTGAATGCAATCTACTTTCACGGTTTTTGGGAAATTCCATTCAAAGCGAACGCCACATTTACCGAAAAATTTCAAGTCAATAGAACCAAAAGTGTAAATG<br>TCAAAATGATGACCCTGGAAGGACCAAGCTTCAATTACAAACATAGTCCAGAATTACAAGCTAAAATATTGCAAATGTTTTATAAGGGAGGTCAGGCCAGTATGGTGATAGTACTGCCAGA<br>TAAGATCGAGGGTGAGACAGCGGGCGTAGGTGGCCTGATGGAACGACTGATAGCCGGGTATGACCTGATGGCAGACATTAAGAGTCTGCAAAAAACACCTCTCAAAGTAATCATACCG<br>AAGTTCAAAGCTGAAACAGAAATAAATTTGAAGGAACTTCTGCCAAAGATGGGAATTAAGGCAGTTTTCGATATGAATAAGGGAGATACAACCATGCTCTCATCTGATGAGCCACTCTAC<br>GTATCGGAAGCCATCCAGAAGGCCTTCATCTCGGTGGATGAGGCAGGAACGGAAGCTGCAGCTGCTACTCAAATGGTCCGACGTCGTCTCTGCGGCCACTGGTGGACCGGCGAGACTT<br>TCGTTGCAAACCGTCCCTTCCTCTACCAACTTACAGCTCGCGAAATTCCTATATTCTATGGAACTTTCTATGGAAATGGATATTCACAATAA	SSNESRAEIFKALDIPNEDNLRSVFTSLTQNLKKPFIETITSILK<br>PFSQEYTLKIANKIYVKQGKDLKSNFKKDAEKVFNASLELIDF<br>GKNDDAANSINNWVAKQTNDIIKDIVSADMLMQDTRVVL<br>VNAIYFHGFWEIPFKANATFTEKFQVNRTKSVNVKMMTLE<br>GPSFNYKHSPELQAKILQMFYKGGQASMVIVLPDKIEGETA<br>GVGGLMERLIAGYDLMADIKSLQKTPLKVIIPKFKAETEINLK<br>ELLPKMGIKAVFDMNKGDTTMLSSDEPLYVSEAIQKAFISV<br>DEAGTEAAAATQMVRRRLCGHWWTGETFVANRPFLYQLT<br>AREIPIFYGTFYGNGYSQ
CpSPN20	chr1	ATGTTCAGGAAAACACTAGATCAAGTTGAACGTAAGTTTTTATTGTCGGCATTTAATAAGTTGACCGCTATTAGTCCAGACAATCGGAAAGAAAACGGGATTAGCTTTTTACAATCAGGGC<br>TTTTCTTATACCACCTGCTGATGTCGCTGTCTCTTACAATGGATCAAAACGCAGTTCGAGAAATAGAAGAATATTTTGGCTTAAATATGTCGGAAGCAGACAAAATCGAGATTTTAGAACGA<br>ACAGTATCATGGCTTCCAAAATCCAGCGAAGAGCTGAAATTCCGGTGGTCGTCGCGGCTGGTGCTGCAGCGGGTGGCAGCTGGAAACGGGCTCCACGGCGCCGCCGCGCGCGTTCTG<br>CCGCTGCGCCTGAGCTGGCTCGTCGGCAACGAAACTTCTACACAACTTGCCGAAACCCTTAACAGTATGGTGTTGTCGGACTCCGGAGGCAGCATGCACGACACGTTCTCGGAGGAGG<br>AGGTGGAGGCGGGCGTGTGCGCGGCGGCGCTGGGCACGGTGTACCTGCGCGGCACGTGGCGCACGGCGCCTACGCTGCTCAACGACACGCTGCCCTTCCGCGACTCGCCCGCCGCG<br>CCCAACCGCTCGTCGCGCTACTTTCGCCTCAACGACCTCATGGCTTACTCCTACTTGCACGAATGGGACACAGAGGCCATAGAGATACCGTACGCTACGCCTGGCCTCACGCTACTCATCC<br>TGGTGCCGAAAGAGCTGTCGCTGCGGCCGTTGGTGGAGCGCGCGGCCGCCGTGGGGCTGGACGCCGTGGTCGGCGTTATGCACACGATGAGGATAGCCGTCACACTACCGCTCTATAC<br>GTTGCGCATGACTTTATTGCTGCCTAATAAACTACAAGATATGGGTATGCAAAGTTTGCTAACGAATGGCACCGACTGTCAGCCTGTGAGGCTGTCCCACGCCGTACAGCGTCTCATGTTC<br>TGGGCAGAAGCTGGTCGAAACGCGTTCAAAGACGATGGTATAGAATGGGACCCGCGTCCAGAATTGGAAATTGTCGTAGACCGTCCATATCTGTTCTTCGTTCGTTGGAATAACGTAACC<br>ATTATGAATGGAGTATTCGTACTGTGA	MFRKTLDQVERKFLLSAFNKLTAISPDNRKENGISFLQSGLFL<br>YHLLMSLSLTMDQNAVREIEEYFGLNMSEADKIEILERTVS<br>WLPKSSEELKFRWSSRLVLQRVAAGNGLHGAAARVLPLRLS<br>WLVGNETSTQLAETLNSMVLSDSGGSMHDTFSEEEVEAG<br>VCAAALGTVYLRGTWRTAPTLLNDTLPFRDSPAAPNRSSRY<br>FRLNDLMAYSYLHEWDTEAIEIPYATPGLTLLILVPKELSLRPL<br>VERAAAVGLDAVVGVMHTMRIAVTLPLYTLRMTLLLPNKL<br>QDMGMQSLLTNGTDCQPVRLSHAVQRLMFWAEAGRNA<br>FKDDGIEWDPRPELEIVVDRPYLFFVRWNNVTIMNGVFVL
CpSPN21	chr15	ATGACATCGATTCACGTTGCGAATTTCGGGGCGCGGTTCTGCAATGAACTGGATAAAAACAAAAACTTCATCTGCTCTCCTCTGTCGGTGGAGACGGTGTTGGCGTTAGTGGCCCTCGGC<br>AGCAGCAACGAATCTCGCGCAGAAATATTCAAGGCACTTAACATCCCTAATGAAGACAATCTTTTTTCCACATTCACATCCCTGACGGCAACTCTAGCTTCGAAGGACCCGTCAGAATTTA<br>AAACATGTGAAGACGTTACATTAAAAATCGCGAACAAGATCTACGTGAAACATGGAGGAGCCCTAAAGGCGGCTCTCAAGAAGGACGCCGAAGAAGTCTTCCAAGCCAGTTTGGAACA<br>AATAGATTTTACACAAAGTAATAATGCTGCGAACGCCATTAATAAATGGGTGTCGAATCAAACTAACAATACTATCAAAGATATTGTTTCTGCCGACATGCTCGATGAAGACACACGGCTTG<br>TACTCATCAATGCTATCTACTTCCATGCTTTCTGGGAGTATAGATTTGACGCCGACGATACATGCACCAGAGTATTTCATGTTAATAACTCCAAGAAAATCGATATTAAACAGATGAGTTTAAT<br>GGGAAAACCTTTTAATTACAAACACAGTCCCGAGTTAAAGGCCAAAATACTGCAGATGTTCTACGTTGGAGGTGAAGCTAGTATGGTGATAGTTCTGCCAGATGAAATCGAGGGTCTAAG<br>TGCTGTGATGAAACAACTGGCAGCCGGGTATGATCTGATGACAGAGATCAGAAGTCTACAAAAAAAAGAACTTGAAGTAATCATACCTAAGTTTAAAATTGAATCGGAAATCAATCTAAA<br>GGAACTATTACCTAAGATGGGAATCAAGACAATTTTTGATCCAATTAAGTCAGAGGTCAACATGTTGTCAAGTGATGATCGACAATTTGTATCCCAAGCCATTCAGAAAGCGTTCATAGCA<br>GTGGATGAACTGGGCACGGAGGCGGCAGCAGCCACATCGATGTGGCAACGAGAACGCTGCGGCCATTTTTGGACCGGCGATACGTTCACGGCGGACCGCCCCTTTCTCTTTCTACTCC<br>AGGCTCATGAGATCCCTGTATTTTATGGAACTTTCAATGGATCAGACATCATTTTCTTTAAATAT	MTSIHVANFGARFCNELDKNKNFICSPLSVETVLALVALGSS<br>NESRAEIFKALNIPNEDNLFSTFTSLTATLASKDPSEFKTCEDV<br>TLKIANKIYVKHGGALKAALKKDAEEVFQASLEQIDFTQSN<br>NAANAINKWVSNQTNNTIKDIVSADMLDEDTRLVLINAIYF<br>HAFWEYRFDADDTCTRVFHVNNSKKIDIKQMSLMGKPFN<br>YKHSPELKAKILQMFYVGGEASMVIVLPDEIEGLSAVMKQL<br>AAGYDLMTEIRSLQKKELEVIIPKFKIESEINLKELLPKMGIKTI<br>FDPIKSEVNMLSSDDRQFVSQAIQKAFIAVDELGTEAAAAT<br>SMWQRERCGHFWTGDTFTADRPFLFLLQAHEIPVFYGTFN<br>GSDIIFFKY
CpSPN22	chr5	ATGTCAGCAATGTCGGTGTTCACTCCGATAGCCGAGCTTTGTCTGTACACGGAGGACGGTCCCGCCTTTGATCAGTGCATCAAAATACTTAACTTGAAGGACAAAAAAGATATCCGAGAT<br>ACCTTTGCCAATTTCTCCTCCTTATACGAAGGTGTGACCAGTGTTAACTTTTCCACTGCCGCCAAAGTGTATTCCAGTGAGAAATACGAGCTGAGCGCTGGTTTCATAAAAGATGCCATTG<br>AAGTCTTCCACGCCGCAGCACAAAATCTTGATTTTAGCAAAAACGTTGAAGCTGCAGCTATAATGAATGCCTGGGTTGAGAGTCAAACTAACAATCGTATTAAGAATTTAGTTACACCAGA<br>TATGATAAGTGCATTAACAAGACTTGTACTCATCAATGCTATTTACTTCAAGGGTAATTGGCTTTACAGATTTAACGAAACTAATACGAAAGAAGAAGATTTCTATCTAAGTGATGGTTCAAC<br>TGCGAAAGTAAAGTTGATGTATCAAGAGATTAATGATCTTGAGTATGGAGAAGTTCCTTCCTTAGACTGCAAGGTGGTTCGAATCCTTTACACTGGCAAAGAAAGCTCCGCGATCATTATT<br>TTGCCAAACAGCATTAACGGCACAATGCAATTGGCGAAAGACCTGCAAAATACAGCGAATTGGAAAAAAGTCATAGATAGCCTTTATCCTCAAAAAGTGAAACTGTATTTACCAAAGTTT<br>GTCATTAGTACAACTACAGACTTAAAGGTTTTGTTACAGAAGTCTGAAGTTAAGTTAATGTTTTATGTCATTATGTGTGTAGTAACTCTGGCCACAGACAGTTAG	MSAMSVFTPIAELCLYTEDGPAFDQCIKILNLKDKKDIRDTF<br>ANFSSLYEGVTSVNFSTAAKVYSSEKYELSAGFIKDAIEVFHA<br>AAQNLDFSKNVEAAAIMNAWVESQTNNRIKNLVTPDMIS<br>ALTRLVLINAIYFKGNWLYRFNETNTKEEDFYLSDGSTAKVKL<br>MYQEINDLEYGEVPSLDCKVVRILYTGKESSAIIILPNSINGT<br>MQLAKDLQNTANWKKVIDSLYPQKVKLYLPKFVISTTTDLK<br>VLLQKSEVKLMFYVIMCVVTLATDS
CpSPN23	chr15	ATGTCCTTCTGTTCTGCAGGAAAACAAGATTTGAGGAGATTGCTCTTTTCCACATTCATATCCCTGACAACAACTCTAGCTTCGAAGCCTTCAAAACATAAAACATCCAACGACGTTACGTT<br>AAAAATCGCGAACAAGATCTACGTGAAACAGGGGGCAGCCCTAAAGGCGGCTCTCAAGAAGAACGCCGAAGAAGTCTTCCAAGCCAGTTTGGAACAAATAGATTTTACACAAAGTAAT<br>AATGCTGCGAATGCCATTAATAAATGGGTGTCAAATCAAACCAACAATACTATTAAAGACATTGTTTCTGCCGACATGCTCGATGAAGACACACGACTAGTACTCATCAATGCCATCTACTTC<br>CACGCTTTCTGGGAGTATCAATTTGACGCCGACGATACATACACCAGAGTATTTCATGTCAATAAATCGAAGAAAATCGATATTAAACAGATGAGTTTAATGGGAAAACCTTTTAATTACAA<br>ACACAGTCCCGAGTTAAAGGCCAAAATACTGCAGATGTTCTACGTTGGAGGTGAAGCTAGTATGGTGATAGTTCTGCCAGATGAAATCGAGGGTCTAAGTGCTGTGATGAAACAACTGG<br>CAACCGGGTATGATCTGATGGCAGAGATCAAAAGTCTACAAAAAAAACAACTTCAAGTAATCATACCTAAGTTTAAAATTGAATCGGAGATCAATCTGAAGGAACTGTTACCTAAGATGG	MSFCSAGKQDLRRLLFSTFISLTTTLASKPSKHKTSNDVTLKI<br>ANKIYVKQGAALKAALKKNAEEVFQASLEQIDFTQSNNAA<br>NAINKWVSNQTNNTIKDIVSADMLDEDTRLVLINAIYFHAF<br>WEYQFDADDTYTRVFHVNKSKKIDIKQMSLMGKPFNYKH<br>SPELKAKILQMFYVGGEASMVIVLPDEIEGLSAVMKQLATG<br>YDLMAEIKSLQKKQLQVIIPKFKIESEINLKELLPKMGIKAIFD

		GAATCAAGGCAATTTTTGATCCAATTAAGTCAGAGGTCAACATGTTGTCAAGTGATGATCGACAATTTGTATCCCAAGCCATTCAAAAGGCCTTCATCGCGGTGGATGAACTGGGCACGG<br>AGGCGGCAGCAGCCAGTCTTCTCACTACATGGCCGTACCAGCGCAAGCGCGATTCTACGACTTTGTCTTGTATCGGCCTGATCTTATATGTAGATTCCGTGGATGTAGACATTAGGCACTTT<br>ATCGAACATGGTCACTCCTGCGGCCTACCTGAGCATCCGCATTTCCACCACATGAACTCTGTTCAGCTGTAA	PIKSEVNMLSSDDRQFVSQAIQKAFIAVDELGTEAAAASLLT<br>TWPYQRKRDSTTLSCIGLILYVDSVDVDIRHFIEHGHSCGLP<br>EHPHFHHMNSVQL
CpSPN24	chr22	ATGGAGCCGTCGCTCGCAGCGTCACTCTCGCATTTCGCAGCGCGGTTCGCGAATGAACTGGAGAAGGGTAAAAGTCAGGTGTGCTCCCCGCTGTCGGCGCAGGCGGTGCTGGCGCTG<br>ACGGCGCTCGGCAGCGGTGGCGACACCCGCAAGGAGCTGCTGGAGGCCATGGCGCTGTCTGATGATGAAGCGCTTCGTTCTTTATTCTCCTACGTAACATCTACCACGAAGTCCGTCGA<br>AGGCATCACGCTGAAGATGGCCAACAAAGTCTATGTCACCAATAAAAGCAAGCTGCAGCCAGACATCCAGAAGGATGCCGAGGAGGTGTTCCAGTCCGCGCTGGAGCAGTTGGACTTT<br>ACGGAGAGTGCAGCAGCTGCGGGGGCTGTTAATAAATGGATGGCCAACAAAGTCTACGTCACCAATAAAAGCAAGTTGCAGCCAGACATCCAGAAGGATGCCGAGGAGGTGTTCCAG<br>TCCGCGCTGGAGCAGTTGGATTTTACGGAGAATGCAGCAGCTGTGGGGGCTGTTAATAAATGGGTGGCGGAGCAAACTGAAGACATGATAAAGGACGTAGTAGACGCCAAGTCACTG<br>AACAGTGACACGCGCCTGTTGCTCATCAACGCCATCTACTTCCAGGGATTTTGGGAGACACCGTTCAGGACTCGAGCCACCCGCGACCGACCGTTCCATGTGGACCTCAACACGCAGAT<br>CAAAGTGCCGATGATGAGCATGCACGATGTCCACTTCCGGTACAAGCACAGCCCGGAATTGAAAGCTCAATTGATAGTAAAAAAAATATTGGGTCTAGTTTTTGACATCAGGATTCATTTC<br>CCATTCCAGTTCCTCCAGATGGACTACAAAGGTCGACAGGCGAGCATGGTGATAGTCCTCCCCGATGAGATCGAGGGTCTCGGAGCCGTGCTGCAGCAGCTCGCCGCCGGCCACGACCT<br>GATGGCTGAACTGACAGATATGAAGAGCACGCTGCTGGACGTCATGGTTCCCAAGTTCAAGATTGAGACGGAGATAGATTTGACGCAGCTTCTGCCTGAGATGGGCATCAAGCTAATAT<br>TCAACGATCGCTATTCAAACATCAATATGCTAACGTCCAACGAGCCCCTCTACGTGTCGAAAGCTGTCCAGAAGGCTCTGATCGAGGTGGACGAGGCAGGGACGAAGGCTGTTGCACGC<br>ACGATGATACCCCCCGAAATGCCTTGTTGTATACGGAGGCCACGGAAGTTTAAAGCGGACCGTCCCTTTGTATATTTACTGGCGGCACGAGAGTTCCCGATTTTCTTGGGAATCTATCAAG<br>GAAGCGAATTTGCAGTACCAGATGTGTCTGATAGTGATATTGACCAAACGAAACCGGTTAACGTCGGTGTCAAAAAGCAATTATCAAAATAA	MEPSLAASLSHFAARFANELEKGKSQVCSPLSAQAVLALTAL<br>GSGGDTRKELLEAMALSDDEALRSLFSYVTSTTKSVEGITLK<br>MANKVYVTNKSKLQPDIQKDAEEVFQSALEQLDFTESAAA<br>AGAVNKWMANKVYVTNKSKLQPDIQKDAEEVFQSALEQL<br>DFTENAAAVGAVNKWVAEQTEDMIKDVVDAKSLNSDTRL<br>LLINAIYFQGFWETPFRTRATRDRPFHVDLNTQIKVPMMS<br>MHDVHFRYKHSPELKAQLIVKKILGLVFDIRIHFPFQFLQMD<br>YKGRQASMVIVLPDEIEGLGAVLQQLAAGHDLMAELTDM<br>KSTLLDVMVPKFKIETEIDLTQLLPEMGIKLIFNDRYSNINML<br>TSNEPLYVSKAVQKALIEVDEAGTKAVARTMIPPEMPCCIRR<br>PRKFKADRPFVYLLAAREFPIFLGIYQGSEFAVPDVSDSDIDQ<br>TKPVNVGVKKQLSK
CpSPN25	chr5	ATGGCGTTGTCAGCAAAACCCTGCCCGGTCAACGAAGAATACTCCTCCTGCATCAACGGTGGATGTGGACCCAGAAACTGCAGTCAGATCGACAAGTCGAGGACCTGTGTGGATCCTGT<br>CAAGTGCATTGAGGGATGCGTCTGTAAGCAGGGCTGGTTGAGGGCCGGAAATGGAACCTGCGTACCTAAGGACCAATGCAGAACAAAATGCGGACTAAACGAAGTGTTCAGCGACTG<br>TCCCAACCCCTGTCCGCCCAGGAAGTGCAACCTCAAAAACGCAGTGTTCAAATGCAAGGCACCACCCAAGCCAGGGGATCCTGAATGCAAGCCTGGGTGCACGTGCGCTAAAAACTATT<br>ACAGAAATGCGACAGGATCGTGTGTGCTGAAGGAGAACTGCGAAAAACCATTAAAATGCCCAGTAAACGAACAGTATGATCCCTGCCCAATGATCTGTCCGCCTCAAAAATGTGATGTCA<br>ACCCAGCTCTGATCCTGTGCCTTCCTAATCCAGAGTATGGCAATCCTAACTGTAAACCAGGCTGCATCTGCAAGAACTTAGAAGTACGGAACGATGCCGGACAATGTGTCCCGAAGGATA<br>AATGCCCAAAATGTACTGGCCAGAACGAATACTGGGAACCCTGCATTCAAGGCTGCTCCAACCAAACCTGCGGAAGCATCGGTCAGAAATACTACTGCCCGTTACAGAAAGGCTGCAAG<br>AGTGGCTGCAGATGCAAGCCCGGATTCTACAGGAAAGATGTTGGATCACCTTGCAGTCTCACAGAGGAACAGTGCACCAAAGGAAAGTGTCCGCCTGGTGAGAATTGGAACACGTGTC<br>CTGCGATCAAATGCGATGCGGATTACTGCGGAAAAAATAAAGATTCACCAAAATGGTGCCCGATTGGGATAGTATGTGACCCGCCAAGATGTACATGCGAAAACAATAAAAAAAGAGAC<br>AGGAAAAATGGCAAATGTATCCCAGTGGTTGACTGTCGAAATGGATATCCATTACCTGAATTGAAAGCAGAAAAAGTAAGGATAGCCAATCTATCAAAAACACCATTCCCATGCCCCCGCA<br>ATGAAGAGTATAAAGTTTGTCCACCACCATGCCCCGGAGAGACGTGTTCTGAGCACATTACCGGGCCGCCGGATGGGCACTGTCCGCCGAACGAGCCCGTGACTATCTGCACTCCACAG<br>TGCAGGTGTTGCAAAGGATATTCCAGAAACGATAATGGAACGTGCATACCAAGCCTTAAGGCAACACCTACAACTACAGTAAAACCGCCTACTATCAGCACCGGTGACAGAGGCTCTGG<br>ATTAGCAGGCGTACTTGATGGCATCACAGCATTCACTGGAAAATGTCTTTATGAACGCATGCAATCTGCACCTAACGAGAACATAATAATGTCCGCCATGTCGGTGTTCACTCCGATAGCTC<br>AGCTTTGTCTCTACTCGAACGGACCCGCCTTTGATCAATGCATCAAAACACTGAATCTTAAGGATAAACAAGACATCCGAGATACATTTGAAAATTTCTCCTCCTTATACGAAGGCGTTACC<br>AGTGTTAACTTTTCCACTGCTGCCAAAGTCTATTCCAGTGATAAGTACCAGCTGAGCGATGGTTTCAAAAAAGATGCCATCGATGTCTTCCACGCCGCAGCACAAGAGATCGATTTCATTA<br>AAAAAAATCAAGCTGCAGCTATAATGAACGCCTGGGTTGAGAGTAAAACCAACAATCGAATTAAAGATCTAGTTTCACCTGACATGCTAAGTGAATCGACAAGGATTGTACTTATCAATGC<br>CATTTACTTCAAGGGTAAATGGTTGAAACAATTTAATGAAAATGATACGAAAAAAGAAGATTTCTATTTAAGCGATGGTTCAACTACAAAAACCAAGTTGATGTATCAAGAAATCAAGGAC<br>CTAGAGTACGGAGAAGATGCATCTTTAGATTGCAAGGTCGTTCGAATTCCTTACACAGGCGAAGAGAGCTCCGCGATAATTGTTTTGCCAAACAGCATTAACGGCACAATGCATTTGGCC<br>CAAAATCTACAATTTCCAGCAAATTGGAAGAAACTCTTAGATAGCCTCTCCCCTCAAAAAGTGCAACTATATTTACCGAAATTTGTAATTAGCACTACTACAGACTTGAAGGACTTATTAAA<br>GAAGGCTAATATAACTAATTGGTTTAACTGCGGCAATTCTGGTTTATCCGGGCTTTTAGCCAAGCCAGAAGATATCTGCATAACAGAGGCCATTCAAAAAGCGTGGTGTGAATTCAATGAA<br>ATAGGAACAGAGGCTACTGCAGCTAATGTTGTAGTAGGCGTAGGTGGCGCCATGCCAATGCCGCCAGTGGTATTTAAAGCTAACCGTAGCTTCCTCTTCTATATTCTACTAAAGAATGCAA<br>GCGAATTGCCCATTTTCTGTGGAAAATACGGAAATCCAAACTAA	MALSAKPCPVNEEYSSCINGGCGPRNCSQIDKSRTCVDPVK<br>CIEGCVCKQGWLRAGNGTCVPKDQCRTKCGLNEVFSDCP<br>NPCPPRKCNLKNAVFKCKAPPKPGDPECKPGCTCAKNYYR<br>NATGSCVLKENCEKPLKCPVNEQYDPCPMICPPQKCDVNP<br>ALILCLPNPEYGNPNCKPGCICKNLEVRNDAGQCVPKDKCP<br>KCTGQNEYWEPCIQGCSNQTCGSIGQKYYCPLQKGCKSGC<br>RCKPGFYRKDVGSPCSLTEEQCTKGKCPPGENWNTCPAIKC<br>DADYCGKNKDSPKWCPIGIVCDPPRCTCENNKKRDRKNGK<br>CIPVVDCRNGYPLPELKAEKVRIANLSKTPFPCPRNEEYKVC<br>PPPCPGETCSEHITGPPDGHCPPNEPVTICTPQCRCCKGYS<br>RNDNGTCIPSLKATPTTTVKPPTISTGDRGSGLAGVLDGITA<br>FTGKCLYERMQSAPNENIIMSAMSVFTPIAQLCLYSNGPAF<br>DQCIKTLNLKDKQDIRDTFENFSSLYEGVTSVNFSTAAKVYSS<br>DKYQLSDGFKKDAIDVFHAAAQEIDFIKKNQAAAIMNAWV<br>ESKTNNRIKDLVSPDMLSESTRIVLINAIYFKGKWLKQFNEN<br>DTKKEDFYLSDGSTTKTKLMYQEIKDLEYGEDASLDCKVVRI<br>PYTGEESSAIIVLPNSINGTMHLAQNLQFPANWKKLLDSLSP<br>QKVQLYLPKFVISTTTDLKDLLKKANITNWFNCGNSGLSGLL<br>AKPEDICITEAIQKAWCEFNEIGTEATAANVVVGVGGAMP<br>MPPVVFKANRSFLFYILLKNASELPIFCGKYGNPN
		ATGTACGGACAAGTACGAGCGGAATGCACTATGACTAATGACATCAATCAGATATTGGCCTCATTGGCCTGGTTCTTGAGTGTATGGGCGGACGAGATGCGTGCGACACTGTGTGTGCTG<br>CTTGCAGCCACCTGCGTATTGGCTGATGAAAGCAGGGGCTCCAAGCGTCCGTACATCGCCGACGCGGTTGGCAATACTTTGCCCCCACCATCTAATGGGCCAGCGACCGTCGGCAGCCC<br>TGTCAACCTCCTAACCCCAGACCGCTATGAGTTCTACACTTTCGATGAATCCGGAGAACTGGTTAAACGACTCATGACTTTACAAGAAATTCAAGCCATAGTTGCGGCTGGTGAAGAAGC<br>AGAAGGACTGGTTACGTTTGCTAATGATAATCAGCCGACGATTGCATTTAATTTCAGTCAACCACCTTACACTAAAGTTCATAGCGTTGTCACTAATGTGCAGAATGTACTCAAGGCTCAAA<br>TGGAAGCTCATAAAAACAAACCACAATTGAATCCTACATTGGATACACCTGATGTGTCTGATTCTTGGAGTCTAATTTTACCATCGATCTTTGGAAACACTGGAGTCGATATCGTACCAGATA<br>AGACTTCTGCATCTTTTACTACCCCAGAAACAGAAACAATTGAGTTTGACCGTATTAACTTTGATGACAATCTTAGCAAGGAAGGAAGTAACCCGATCGGAAGTTCCACTACAGTACAAAA	MYGQVRAECTMTNDINQILASLAWFLSVWADEMRATLCV<br>LLAATCVLADESRGSKRPYIADAVGNTLPPPSNGPATVGSPV<br>NLLTPDRYEFYTFDESGELVKRLMTLQEIQAIVAAGEEAEGL<br>VTFANDNQPTIAFNFSQPPYTKVHSVVTNVQNVLKAQME<br>AHKNKPQLNPTLDTPDVSDSWSLILPSIFGNTGVDIVPDKT<br>SASFTTPETETIEFDRINFDDNLSKEGSNPIGSSTTVQNVAKP

CpSPN26	chr16	TGTTGCAAAGCCATCAATAACAGAAAAAATTTCGTCTACTACAGAATTGTTTAAAGAAGTTCCAACAACTGCTAAAACAACTACTACAACGACCAGCACTACTACGCCTAAACCTACTACTA<br>CTTCTACGACTACGACGCCTAAACCTACTACTACAACTACACAACTAAAAAACCTACTACTACTACCACTACCACAACATCGACTCCAACCACTCCTAAACCTAAAATCACCACCACCACTAC<br>TACTACTAGAAAACCTACTACGACTACTACTACGTACTACTACTACTACCACTACTTCTAAGCCACGAACGACTGCCTCAACTACAAAATTAACTACAACTTCTAAACCAACTGCTAAAATTAC<br>TTCGAAACCTACCACAGCTAAACCTATTTCTACCACTCAACGACAGGTTGCAACTTCAACTTCGAAAAACACAGAAATACACATGCAACTTCTACAACAGAAAAAAACTAGCCCTGTATAT<br>GAGAAGATCAGTACGTTTGTTCCAATTTCTACAGCAGCCTATGTAACTGAGAGATCTACACGAAAACCTACTCAAACGACTAAATTCGTCAGCACATCGGAAAGTACTACACAACCGCCAG<br>TATCGTCAATATCAAAGCTTAAAGAATCAACTACTTTAGAGGCAGAAACGCAACCAACTATTACAACTTACAAACCAACGACTAGCACACCAGCACCAGTTTCATCTACTGTTAAAAGCGG<br>TGAACTTGAAAAAGATGATGCTAGTACCATAACCACTCAATCTGTATCACCTTCAACTGCCATCTCATCAACAGAATTTATGTCTGTTGAAAATCAGCCACCGCAAATGATCAATGATAATAT<br>TTCAATTTCAGAAGACAATGATATTAAAGTCAATGAACCCATTATTCCTTTGTTTGATGTTGCACAAAGTATCAGTCAGATTGCATCAGACCTTGGAAGTGCCAACTACCAACCTATCCCAAC<br>AACAAGTGGTTTATTAGACACTACACACGCTATGAACATGGATTTAGAAAGCAAAGAAAGTTTAGAATTGGACATCCCATCGGAGTCTAAAAACGAGTCTCAAAATAAAACGGCTGATAT<br>TCAAAACGAAAATGATAATTTCGTCAAAATATCTACGATTAACCCCCATCAACAAAATAATAATCCGCCACTGAAAGTCAAAGAAACAATAAATAAAGTAAGCAACCCGGTATCAAAGACTA<br>CCGTTGGACTAGAAACTACTACCTCAACATCAACTGAAACACCTATGGATCCTATTCTATCAGAATCTATGGATGATTTGTTATCTCAAGTTGTGAATCAGGCTCCTCAATCCATTGTTGATAG<br>TGAAAATAAACTTCAAGAGTCCAATGTAATATCGACAGCCGAAAATTTTGTAGACACAACTACAGTTGTTATTACTACTAAAACCGAGATTATTTCTCAAGAATCTACGAAAAATGACATGT<br>CTTCAACAACCGAGCTGGCGCTGGTTGATACAACCATTAATCAAAATTTAATAAAAAATGAAACAAATGACGATGTCACTGAAAAAGGAGATAATAAGAAAGATACAATAACACAAACAG<br>AAAACACAGTTAATGCCAATAGTGTTTCTGCAACACAAAATGATTTAACAAAAATCTCTTCCTCCAATAGCACTGAGAAACCATCAAATCTTATAAATTCCCACGGGATTCAAACTCTTAGT<br>GTTGACAAACAAAATAATGTAATTGCCGTTATAAATGCACCCGGATTGCCTGTGAAAGAACAAATTACACCATCAAGCACTGAAACTGTATCACCACAAGAGGTTTCACATAAAGCAGGCT<br>CAGAAAATCAAAATAAAATAAATGATAAAGTTAGAGATGACCATAAAGAAGAAACGGTTTCTCTTCCAAAGGTAGATGAATTCAAAAAGAAGATTCAGAAAGTCAATATTGACTACAAAG<br>AAATCGCCAATGAAAGAAATAACTCTTGGAAGCTTATCTCAACTGTTGCACCGCCGAAAGCAAATGAGATACCGAAAGATAAATTTAAAACTGACGGCAATAACCCAGAAAGCCCTGAT<br>AAAGATATAGTTTTGGACGTATCTAAGGAAAATCAAGGACTGGAGGTGACCACAAAAGACTTAAGCGATGATGTCGCGCAATTTACTGAACTTTGCAATGAGCTCGCTTTCCGGTATTGG<br>AATGCTATCGCTGAAACTATTCCAAATAAGAGGAGTTTTATTTTGTCCCCATTTTCTATCACATCAATGTTGGCTATGATGTTCATGGGAGCAAGAGGCGCAACTTCGGGTGAAATGAACGA<br>AATACTGAAACTTGATGATATGGTCACTTTTAATCCCCACTTTACCCTAAAAAATATTTCCGATTCTATAGAAACGACACCTGAGTCTGGAGTTGCTATCTCCGCGTTTATAAGAGAACTTTAC<br>AGCGACCGAAATAAAGGCCAAATTCTTACTTTTTACAAGGAGCGGGCTCAGCATTTCTACAATGGTCATGTTGAAGAAATCAACTTCAAATTGATTAGCGATATTATAAGAAGAAGGACG<br>AATCTGTTGGTAAAGAGGTACACTTGGGGAAAAATAATTGAGTACATGAAAGCGAACACAATTACTATGCAACCTCCGCTTGCTGCGTTTTCAACAAATATTTTTGAGACTGACTGCACG<br>GGCTCATCTGTAGAAGGCAGAGATGGTGAAATGTACTTCGTAGTCTCACCCAACGTGCGTCAACGTCGCCTCGTCCCTGTGCCAGCCGTTCTCTACAAGAAAGGATTCCTCGCTGGCTAT<br>GATCCAGTCCTCGACGCCACTGCTGCAGCCCTCGGCAACACTAACTCAATCATCAGCACACTCTTCCTCATGCCAGGCCAGCAAGGCAACGTTGTACACGCCGAAGATCTAGAGCTACTA<br>GAAAAACGATTGCTAGGCTCCAACCCAAGTACACCAGCGTGGAATCGCCTTCTCAGAACGTTATTACCACGAATTGGATTGGAACTTCAAATCCCGCGCTTCTCTCACAAGTCAATCTTTA<br>ATGTATCTTCGACTCTTAAAAAGATGGGATTGAAAGACTTGTTCGATGCCGAGCACGCTGATTTAGGAGGTCTGAATGGCCCGTCTAAGGATCTTTACTTGTCGGACATGGTGCAAGCGA<br>CGACGTTTGTTACTTGTGGCGAAGGAATTATTGGTGAGCAACATCATATAGAAGAGTATCCTGAGAGTATTGAATTGGCCAGAAGGCGTAGAACGTCTCGATGGGCGACGGGATGGGAT<br>GAGCCGAGGGATTACCAGAGAGCATTCCATGATCCGCATGACGTTGGAGATGCCATGAATCTACCACTGCATTTGCGCCCAAGACAAGCTCGTCTCCCAGCGAGGAACGCCCAGCCAGC<br>AAGATTAAAGTTTGACCGGCCGTTCCTTTACTTCGTAAGACACAATCCCTCTGGCATGATACTCTACGTCGGCCGGTACAATCCACGGCTTTTGCCTTGA	SITEKISSTTELFKEVPTTAKTTTTTTSTTTPKPTTTSTTTTPKP<br>TTTTTQLKNLLLLPLPQHRLQPLLNLKSPPPLLLLENLLRLLLR<br>TTTTTTTTSKPRTTASTTKLTTTSKPTAKITSKPTTAKPISTTQR<br>QVATSTSKNTEIHMQLLQQKKTSPVYEKISTFVPISTAAYVTE<br>RSTRKPTQTTKFVSTSESTTQPPVSSISKLKESTTLEAETQPTI<br>TTYKPTTSTPAPVSSTVKSGELEKDDASTITTQSVSPSTAISST<br>EFMSVENQPPQMINDNISISEDNDIKVNEPIIPLFDVAQSIS<br>QIASDLGSANYQPIPTTSGLLDTTHAMNMDLESKESLELDIP<br>SESKNESQNKTADIQNENDNFVKISTINPHQQNNNPPLKV<br>KETINKVSNPVSKTTVGLETTTSTSTETPMDPILSESMDDLL<br>SQVVNQAPQSIVDSENKLQESNVISTAENFVDTTTVVITTKT<br>EIISQESTKNDMSSTTELALVDTTINQNLIKNETNDDVTEKG<br>DNKKDTITQTENTVNANSVSATQNDLTKISSSNSTEKPSNLI<br>NSHGIQTLSVDKQNNVIAVINAPGLPVKEQITPSSTETVSPQ<br>EVSHKAGSENQNKINDKVRDDHKEETVSLPKVDEFKKKIQK<br>VNIDYKEIANERNNSWKLISTVAPPKANEIPKDKFKTDGNN<br>PESPDKDIVLDVSKENQGLEVTTKDLSDDVAQFTELCNELA<br>FRYWNAIAETIPNKRSFILSPFSITSMLAMMFMGARGATSG<br>EMNEILKLDDMVTFNPHFTLKNISDSIETTPESGVAISAFIRE<br>LYSDRNKGQILTFYKERAQHFYNGHVEEINFKLISDIIRRRTN<br>LLVKRYTWGKIIEYMKANTITMQPPLAAFSTNIFETDCTGSS<br>VEGRDGEMYFVVSPNVRQRRLVPVPAVLYKKGFLAGYDPV<br>LDATAAALGNTNSIISTLFLMPGQQGNVVHAEDLELLEKRLL<br>GSNPSTPAWNRLLRTLLPRIGLELQIPRFSHKSIFNVSSTLKK<br>MGLKDLFDAEHADLGGLNGPSKDLYLSDMVQATTFVTCGE<br>GIIGEQHHIEEYPESIELARRRRTSRWATGWDEPRDYQRAF<br>HDPHDVGDAMNLPLHLRPRQARLPARNAQPARLKFDRPF<br>LYFVRHNPSGMILYVGRYNPRLLP

Note: *The underlined part of amino acid sequences represents predicted signal peptides.
